# THE CONCISE GUIDE TO PHARMACOLOGY 2019/20: Catalytic receptors

**DOI:** 10.1111/bph.14751

**Published:** 2019-11-11

**Authors:** Stephen PH Alexander, Doriano Fabbro, Eamonn Kelly, Alistair Mathie, John A Peters, Emma L Veale, Jane F Armstrong, Elena Faccenda, Simon D Harding, Adam J Pawson, Joanna L Sharman, Christopher Southan, Jamie A Davies, CGTP Collaborators, Clare Bryant, Richard W Farndale, Adrian Hobbs, Gavin E Jarvis, David MacEwan, Tom P Monie, Scott Waldman

**Affiliations:** ^1^ School of Life Sciences University of Nottingham Medical School Nottingham NG7 2UH UK; ^2^ PIQUR Therapeutics Basel 4057 Switzerland; ^3^ School of Physiology, Pharmacology and Neuroscience University of Bristol Bristol BS8 1TD UK; ^4^ Medway School of Pharmacy The Universities of Greenwich and Kent at Medway Anson Building, Central Avenue, Chatham Maritime Chatham Kent ME4 4TB UK; ^5^ Neuroscience Division, Medical Education Institute, Ninewells Hospital and Medical School University of Dundee Dundee DD1 9SY UK; ^6^ Centre for Discovery Brain Sciences University of Edinburgh Edinburgh EH8 9XD UK; ^7^ Cambridge UK; ^8^ London UK; ^9^ Liverpool UK; ^10^ Philadelphia USA

## Abstract

The Concise Guide to PHARMACOLOGY 2019/20 is the fourth in this series of biennial publications. The Concise Guide provides concise overviews of the key properties of nearly 1800 human drug targets with an emphasis on selective pharmacology (where available), plus links to the open access knowledgebase source of drug targets and their ligands (http://www.guidetopharmacology.org), which provides more detailed views of target and ligand properties. Although the Concise Guide represents approximately 400 pages, the material presented is substantially reduced compared to information and links presented on the website. It provides a permanent, citable, point‐in‐time record that will survive database updates. The full contents of this section can be found at http://onlinelibrary.wiley.com/doi/10.1111/bph.14751. Catalytic receptors are one of the six major pharmacological targets into which the Guide is divided, with the others being: G protein‐coupled receptors, ion channels, nuclear hormone receptors, enzymes and transporters. These are presented with nomenclature guidance and summary information on the best available pharmacological tools, alongside key references and suggestions for further reading. The landscape format of the Concise Guide is designed to facilitate comparison of related targets from material contemporary to mid‐2019, and supersedes data presented in the 2017/18, 2015/16 and 2013/14 Concise Guides and previous Guides to Receptors and Channels. It is produced in close conjunction with the International Union of Basic and Clinical Pharmacology Committee on Receptor Nomenclature and Drug Classification (NC‐IUPHAR), therefore, providing official IUPHAR classification and nomenclature for human drug targets, where appropriate.

1

### Conflict of interest

The authors state that there are no conflicts of interest to disclose.

### Overview

Catalytic receptors are cell‐surface proteins, usually dimeric in nature, which encompass ligand binding and functional domains in one polypeptide chain. The ligand binding domain is placed on the extracellular surface of the plasma membrane and separated from the functional domain by a single transmembrane‐spanning domain of 20‐25 hydrophobic amino acids. The functional domain on the intracellular face of the plasma membrane has catalytic activity, or interacts with particular enzymes, giving the superfamily of receptors its name. Endogenous agonists of the catalytic receptor superfamily are peptides or proteins, the binding of which may induce dimerization of the receptor, which is the functional version of the receptor.

Amongst the catalytic receptors, particular subfamilies may be readily identified dependent on the function of the enzymatic portion of the receptor. The smallest group is the particulate guanylyl cyclases of the natriuretic peptide receptor family. The most widely recognized group is probably the receptor tyrosine kinase (RTK) family, epitomized by the neurotrophin receptor family, where a crucial initial step is the activation of a signalling cascade by autophosphorylation of the receptor on intracellular tyrosine residue(s) catalyzed by enzyme activity intrinsic to the receptor. A third group is the extrinsic protein tyrosine kinase receptors, where the catalytic activity resides in a separate protein from the binding site. Examples of this group include the GDNF and ErbB receptor families, where one, catalytically silent, member of the heterodimer is activated upon binding the ligand, causing the second member of the heterodimer, lacking ligand binding capacity, to initiate signaling through tyrosine phosphorylation. A fourth group, the receptor threonine/serine kinase (RTSK) family, exemplified by TGF‐β and BMP receptors, has intrinsic serine/threonine protein kinase activity in the heterodimeric functional unit. A fifth group is the receptor tyrosine phosphatases (RTP), which appear to lack cognate ligands, but may be triggered by events such as cell:cell contact and have identified roles in the skeletal, hematopoietic and immune systems.

A further group of catalytic receptors for the Guide is the integrins, which have roles in cell:cell communication, often associated with signaling in the blood.

### 1.1 Family structure

S248 Cytokine receptor family

S249 IL‐2 receptor family

S251 IL‐3 receptor family

S252 IL‐6 receptor family

S254 IL‐12 receptor family

S255 Prolactin receptor family

S256 Interferon receptor family

S257 IL‐10 receptor family

S258 Immunoglobulin‐like family of IL‐1 receptors

S259 IL‐17 receptor family

S259 GDNF receptor family

S260 Integrins

S264 Pattern recognition receptors

S264 Toll‐like receptor family

S266 NOD‐like receptor family

S268 RIG‐I‐like receptor family

S269 Receptor guanylyl cyclase (RGC) family

S269 Transmembrane guanylyl cyclases

S270 Nitric oxide (NO)‐sensitive (soluble) guanylyl cyclase

– http://www.guidetopharmacology.org/GRAC/FamilyDisplayForward?familyId=699

– http://www.guidetopharmacology.org/GRAC/FamilyDisplayForward?familyId=686

S271 Receptor tyrosine kinases (RTKs)

S272 Type I RTKs: ErbB (epidermal growth factor) receptor family

S273 Type II RTKs: Insulin receptor family

S274 Type III RTKs: PDGFR, CSFR, Kit, FLT3 receptor family

S275 Type IV RTKs: VEGF (vascular endothelial growth factor) receptor family

S275 Type V RTKs: FGF (fibroblast growth factor) receptor family

S276 Type VI RTKs: PTK7/CCK4

S277 Type VII RTKs: Neurotrophin receptor/ Trk family

S278 Type VIII RTKs: ROR family

S278 Type IX RTKs: MuSK

S279 Type X RTKs: HGF (hepatocyte growth factor) receptor family

S279 Type XI RTKs: TAM (TYRO3‐, AXL‐ and MER‐TK) receptor family

S280 Type XII RTKs: TIE family of angiopoietin receptors

S280 Type XIII RTKs: Ephrin receptor family

S281 Type XIV RTKs: RET

S282 Type XV RTKs: RYK

S282 Type XVI RTKs: DDR (collagen receptor) family

S283 Type XVII RTKs: ROS receptors

S283 Type XVIII RTKs: LMR family

S284 Type XIX RTKs: Leukocyte tyrosine kinase (LTK) receptor family

S284 Type XX RTKs: STYK1

– http://www.guidetopharmacology.org/GRAC/FamilyDisplayForward?familyId=687

S286 Receptor serine/threonine kinase (RSTK) family

S286 Type I receptor serine/threonine kinases

S287 Type II receptor serine/threonine kinases

S287 Type III receptor serine/threonine kinases

S287 RSTK functional heteromers

S289 Receptor tyrosine phosphatase (RTP) family

S291 Tumour necrosis factor (TNF) receptor family

## http://www.guidetopharmacology.org/GRAC/FamilyDisplayForward?familyId=301

### 1 Overview

Cytokines are not a clearly defined group of agents, other than having an impact on immune signalling pathways, although many cytokines have effects on other systems, such as in development. A feature of some cytokines, which allows them to be distinguished from hormones, is that they may be produced by “non‐secretory” cells, for example, endothelial cells. Within the cytokine receptor family, some subfamilies may be identified, which are described elsewhere in the Guide to PHARMACOLOGY, receptors for the http://www.guidetopharmacology.org/GRAC/FamilyDisplayForward?familyId=334, the http://www.guidetopharmacology.org/GRAC/FamilyDisplayForward?familyId=303 family and the http://www.guidetopharmacology.org/GRAC/FamilyDisplayForward?familyId=14. Within this group of records are described Type I cytokine receptors, typified by interleukin receptors, and Type II cytokine receptors, exemplified by interferon receptors. These receptors possess a conserved extracellular region, known as the cytokine receptor homology domain (CHD), along with a range of other structural modules, including extracellular immunoglobulin (Ig)‐like and fibronectin type III (FBNIII)‐like domains, a transmembrane domain, and intracellular homology domains. An unusual feature of this group of agents is the existence of soluble and decoy receptors. These bind cytokines without allowing signalling to occur. A further attribute is the production of endogenous antagonist molecules, which bind to the receptors selectively and prevent signalling. A commonality of these families of receptors is the ligand‐induced homo‐ or hetero‐oligomerisation, which results in the recruitment of intracellular protein partners to evoke cellular responses, particularly in inflammatory or haematopoietic signalling. Although not an exclusive signalling pathway, a common feature of the majority of cytokine receptors is activation of the JAK/STAT pathway. This cascade is based around the protein tyrosine kinase activity of the Janus kinases (JAK), which phosphorylate the receptor and thereby facilitate the recruitment of signal transducers and activators of transcription (STATs). The activated homo‐ or heterodimeric STATs function principally as transcription factors in the nucleus. **Type I cytokine receptors** are characterized by two pairs of conserved cysteines linked via disulfide bonds and a C‐terminal WSXWS motif within their CHD. Type I receptors are commonly classified into five groups, based on sequence and structual homology of the receptor and its cytokine ligand, which is potentially more reflective of evolutionary relationships than an earlier scheme based on the use of common signal transducing chains within a receptor complex.

**Type II cytokine receptors** also have two pairs of conserved cysteines but with a different arrangement to Type I and also lack the WSXWS motif.

## http://www.guidetopharmacology.org/GRAC/FamilyDisplayForward?familyId=305

### 1 Overview

The IL‐2 receptor family consists of one or more ligand‐selective subunits, and a common γ chain (γc): *IL2RG*, http://www.uniprot.org/uniprot/P31785), though IL‐4 and IL‐7 receptors can form complexes with other receptor chains. Receptors of this family associate with Jak1 and Jak3, primarily activating Stat5, although certain family members can also activate Stat1, Stat3, or Stat6. Ro264550 has been described as a selective IL‐2 receptor antagonist, which binds to IL‐2 [211].

**Table:** 

Nomenclature	http://www.guidetopharmacology.org/GRAC/ObjectDisplayForward?objectId=2297	http://www.guidetopharmacology.org/GRAC/ObjectDisplayForward?objectId=2298	http://www.guidetopharmacology.org/GRAC/ObjectDisplayForward?objectId=2299	http://www.guidetopharmacology.org/GRAC/ObjectDisplayForward?objectId=2300	http://www.guidetopharmacology.org/GRAC/ObjectDisplayForward?objectId=2301
Subunits	http://www.guidetopharmacology.org/GRAC/ObjectDisplayForward?objectId=1695 (Ligand‐binding subunit), http://www.guidetopharmacology.org/GRAC/ObjectDisplayForward?objectId=1696 (Ligand‐binding subunit), http://www.guidetopharmacology.org/GRAC/ObjectDisplayForward?objectId=2303 (Other subunit)	http://www.guidetopharmacology.org/GRAC/ObjectDisplayForward?objectId=1697 (Ligand‐binding subunit), http://www.guidetopharmacology.org/GRAC/ObjectDisplayForward?objectId=2303 (Other subunit)	http://www.guidetopharmacology.org/GRAC/ObjectDisplayForward?objectId=1697 (Ligand‐binding subunit), http://www.guidetopharmacology.org/GRAC/ObjectDisplayForward?objectId=1700 (Other subunit)	http://www.guidetopharmacology.org/GRAC/ObjectDisplayForward?objectId=1698 (Ligand‐binding subunit), http://www.guidetopharmacology.org/GRAC/ObjectDisplayForward?objectId=2303 (Other subunit)	http://www.guidetopharmacology.org/GRAC/ObjectDisplayForward?objectId=1699 (Ligand‐binding subunit), http://www.guidetopharmacology.org/GRAC/ObjectDisplayForward?objectId=2303 (Other subunit)
Endogenous agonists	http://www.guidetopharmacology.org/GRAC/LigandDisplayForward?ligandId=4985 (https://www.genenames.org/data/gene‐symbol‐report/#!/hgnc_id/HGNC:6001, http://www.uniprot.org/uniprot/P60568)	http://www.guidetopharmacology.org/GRAC/LigandDisplayForward?ligandId=4996 (https://www.genenames.org/data/gene‐symbol‐report/#!/hgnc_id/HGNC:6014, http://www.uniprot.org/uniprot/P05112)	http://www.guidetopharmacology.org/GRAC/LigandDisplayForward?ligandId=4980 (https://www.genenames.org/data/gene‐symbol‐report/#!/hgnc_id/HGNC:5973, http://www.uniprot.org/uniprot/P35225), http://www.guidetopharmacology.org/GRAC/LigandDisplayForward?ligandId=4996 (https://www.genenames.org/data/gene‐symbol‐report/#!/hgnc_id/HGNC:6014, http://www.uniprot.org/uniprot/P05112)	http://www.guidetopharmacology.org/GRAC/LigandDisplayForward?ligandId=4999 (https://www.genenames.org/data/gene‐symbol‐report/#!/hgnc_id/HGNC:6023, http://www.uniprot.org/uniprot/P13232)	http://www.guidetopharmacology.org/GRAC/LigandDisplayForward?ligandId=5000 (https://www.genenames.org/data/gene‐symbol‐report/#!/hgnc_id/HGNC:6029, http://www.uniprot.org/uniprot/P15248)
Endogenous antagonists	http://www.guidetopharmacology.org/GRAC/LigandDisplayForward?ligandId=5878 (https://www.genenames.org/data/gene‐symbol‐report/#!/hgnc_id/HGNC:6000, http://www.uniprot.org/uniprot/P18510)	–	–	–	–
Selective antagonists	http://www.guidetopharmacology.org/GRAC/LigandDisplayForward?ligandId=4861 [http://www.ncbi.nlm.nih.gov/pubmed/8940020?dopt=AbstractPlus]	–	–	–	–

**Table:** 

Nomenclature	http://www.guidetopharmacology.org/GRAC/ObjectDisplayForward?objectId=1701	http://www.guidetopharmacology.org/GRAC/ObjectDisplayForward?objectId=2302	http://www.guidetopharmacology.org/GRAC/ObjectDisplayForward?objectId=2304	http://www.guidetopharmacology.org/GRAC/ObjectDisplayForward?objectId=2305
HGNC, UniProt	https://www.genenames.org/data/gene‐symbol‐report/#!/hgnc_id/HGNC:5975, http://www.uniprot.org/uniprot/Q14627	–	–	–
Subunits	–	http://www.guidetopharmacology.org/GRAC/ObjectDisplayForward?objectId=1696 (Ligand‐binding subunit), http://www.guidetopharmacology.org/GRAC/ObjectDisplayForward?objectId=1702 (Ligand‐binding subunit), http://www.guidetopharmacology.org/GRAC/ObjectDisplayForward?objectId=2303 (Other subunit)	http://www.guidetopharmacology.org/GRAC/ObjectDisplayForward?objectId=1703 (Ligand‐binding subunit), http://www.guidetopharmacology.org/GRAC/ObjectDisplayForward?objectId=2303 (Other subunit)	http://www.guidetopharmacology.org/GRAC/ObjectDisplayForward?objectId=1698 (Ligand‐binding subunit), http://www.guidetopharmacology.org/GRAC/ObjectDisplayForward?objectId=1704 (Other subunit)
Endogenous agonists	–	http://www.guidetopharmacology.org/GRAC/LigandDisplayForward?ligandId=4981 (https://www.genenames.org/data/gene‐symbol‐report/#!/hgnc_id/HGNC:5977, http://www.uniprot.org/uniprot/P40933) [http://www.ncbi.nlm.nih.gov/pubmed/19710453?dopt=AbstractPlus]	http://www.guidetopharmacology.org/GRAC/LigandDisplayForward?ligandId=4987 (https://www.genenames.org/data/gene‐symbol‐report/#!/hgnc_id/HGNC:6005, http://www.uniprot.org/uniprot/Q9HBE4)	http://www.guidetopharmacology.org/GRAC/LigandDisplayForward?ligandId=5083 (https://www.genenames.org/data/gene‐symbol‐report/#!/hgnc_id/HGNC:30743, http://www.uniprot.org/uniprot/Q969D9)
Comments	Decoy receptor that binds http://www.guidetopharmacology.org/GRAC/LigandDisplayForward?ligandId=4980 (https://www.genenames.org/data/gene‐symbol‐report/#!/hgnc_id/HGNC:5973, http://www.uniprot.org/uniprot/P35225) as a monomer.	–	–	–

### 2 Subunits

**Table:** 

Nomenclature	http://www.guidetopharmacology.org/GRAC/ObjectDisplayForward?objectId=1695	http://www.guidetopharmacology.org/GRAC/ObjectDisplayForward?objectId=1696	http://www.guidetopharmacology.org/GRAC/ObjectDisplayForward?objectId=2303	http://www.guidetopharmacology.org/GRAC/ObjectDisplayForward?objectId=1697	http://www.guidetopharmacology.org/GRAC/ObjectDisplayForward?objectId=1698
HGNC, UniProt	https://www.genenames.org/data/gene‐symbol‐report/#!/hgnc_id/HGNC:6008, http://www.uniprot.org/uniprot/P01589	https://www.genenames.org/data/gene‐symbol‐report/#!/hgnc_id/HGNC:6009, http://www.uniprot.org/uniprot/P14784	https://www.genenames.org/data/gene‐symbol‐report/#!/hgnc_id/HGNC:6010, http://www.uniprot.org/uniprot/P31785	https://www.genenames.org/data/gene‐symbol‐report/#!/hgnc_id/HGNC:6015, http://www.uniprot.org/uniprot/P24394	https://www.genenames.org/data/gene‐symbol‐report/#!/hgnc_id/HGNC:6024, http://www.uniprot.org/uniprot/P16871
Antibodies	http://www.guidetopharmacology.org/GRAC/LigandDisplayForward?ligandId=6880 (Binding) (p*K*_d_ *>* 8) [182], http://www.guidetopharmacology.org/GRAC/LigandDisplayForward?ligandId=6879 (Binding)	–	–	http://www.guidetopharmacology.org/GRAC/LigandDisplayForward?ligandId=7574 (Binding) (pIC_50_ 11.1) [146]	–

**Table:** 

Nomenclature	http://www.guidetopharmacology.org/GRAC/ObjectDisplayForward?objectId=1699	http://www.guidetopharmacology.org/GRAC/ObjectDisplayForward?objectId=1700	http://www.guidetopharmacology.org/GRAC/ObjectDisplayForward?objectId=1702	http://www.guidetopharmacology.org/GRAC/ObjectDisplayForward?objectId=1703	http://www.guidetopharmacology.org/GRAC/ObjectDisplayForward?objectId=1704
HGNC, UniProt	https://www.genenames.org/data/gene‐symbol‐report/#!/hgnc_id/HGNC:6030, http://www.uniprot.org/uniprot/Q01113	https://www.genenames.org/data/gene‐symbol‐report/#!/hgnc_id/HGNC:5974, http://www.uniprot.org/uniprot/P78552	https://www.genenames.org/data/gene‐symbol‐report/#!/hgnc_id/HGNC:5978, http://www.uniprot.org/uniprot/Q13261	https://www.genenames.org/data/gene‐symbol‐report/#!/hgnc_id/HGNC:6006, http://www.uniprot.org/uniprot/Q9HBE5	https://www.genenames.org/data/gene‐symbol‐report/#!/hgnc_id/HGNC:14281, http://www.uniprot.org/uniprot/Q9HC73

### 3 Further reading on IL‐2 receptor family

Leonard WJ *et al*. (2019) The γc Family of Cytokines: Basic Biology to Therapeutic Ramifications *Immunity* **50**: 832‐850

## http://www.guidetopharmacology.org/GRAC/FamilyDisplayForward?familyId=306

### 1 Overview

The IL‐3 receptor family signal through a receptor complex comprising of a ligand‐specific α subunit and a common β chain (*CSF2RB*, http://www.uniprot.org/uniprot/P32927), which is associated with Jak2 and signals primarily through Stat5.

**Table:** 

Nomenclature	http://www.guidetopharmacology.org/GRAC/ObjectDisplayForward?objectId=2307	http://www.guidetopharmacology.org/GRAC/ObjectDisplayForward?objectId=2308	http://www.guidetopharmacology.org/GRAC/ObjectDisplayForward?objectId=2309
Subunits	http://www.guidetopharmacology.org/GRAC/ObjectDisplayForward?objectId=1705 (Ligand‐binding subunit), http://www.guidetopharmacology.org/GRAC/ObjectDisplayForward?objectId=2306 (Other subunit)	http://www.guidetopharmacology.org/GRAC/ObjectDisplayForward?objectId=1706 (Ligand‐binding subunit), http://www.guidetopharmacology.org/GRAC/ObjectDisplayForward?objectId=2306 (Other subunit)	http://www.guidetopharmacology.org/GRAC/ObjectDisplayForward?objectId=1707 (Ligand‐binding subunit), http://www.guidetopharmacology.org/GRAC/ObjectDisplayForward?objectId=2306 (Other subunit)
Endogenous agonists	http://www.guidetopharmacology.org/GRAC/LigandDisplayForward?ligandId=4994 (https://www.genenames.org/data/gene‐symbol‐report/#!/hgnc_id/HGNC:6011, http://www.uniprot.org/uniprot/P08700)	http://www.guidetopharmacology.org/GRAC/LigandDisplayForward?ligandId=4997 (https://www.genenames.org/data/gene‐symbol‐report/#!/hgnc_id/HGNC:6016, http://www.uniprot.org/uniprot/P05113)	http://www.guidetopharmacology.org/GRAC/LigandDisplayForward?ligandId=4934 (https://www.genenames.org/data/gene‐symbol‐report/#!/hgnc_id/HGNC:2438, http://www.uniprot.org/uniprot/P09919), http://www.guidetopharmacology.org/GRAC/LigandDisplayForward?ligandId=4942 (https://www.genenames.org/data/gene‐symbol‐report/#!/hgnc_id/HGNC:2434, http://www.uniprot.org/uniprot/P04141)
Selective antagonists	–	http://www.guidetopharmacology.org/GRAC/LigandDisplayForward?ligandId=5090 [http://www.ncbi.nlm.nih.gov/pubmed/12469943?dopt=AbstractPlus]	–

### 2 Subunits

**Table:** 

Nomenclature	http://www.guidetopharmacology.org/GRAC/ObjectDisplayForward?objectId=1705	http://www.guidetopharmacology.org/GRAC/ObjectDisplayForward?objectId=1706	http://www.guidetopharmacology.org/GRAC/ObjectDisplayForward?objectId=1707	http://www.guidetopharmacology.org/GRAC/ObjectDisplayForward?objectId=2306
HGNC, UniProt	https://www.genenames.org/data/gene‐symbol‐report/#!/hgnc_id/HGNC:6012, http://www.uniprot.org/uniprot/P26951	https://www.genenames.org/data/gene‐symbol‐report/#!/hgnc_id/HGNC:6017, http://www.uniprot.org/uniprot/Q01344	https://www.genenames.org/data/gene‐symbol‐report/#!/hgnc_id/HGNC:2435, http://www.uniprot.org/uniprot/P15509	https://www.genenames.org/data/gene‐symbol‐report/#!/hgnc_id/HGNC:2436, http://www.uniprot.org/uniprot/P32927
Endogenous agonists	http://www.guidetopharmacology.org/GRAC/LigandDisplayForward?ligandId=4994 (https://www.genenames.org/data/gene‐symbol‐report/#!/hgnc_id/HGNC:6011, http://www.uniprot.org/uniprot/P08700)	http://www.guidetopharmacology.org/GRAC/LigandDisplayForward?ligandId=4997 (https://www.genenames.org/data/gene‐symbol‐report/#!/hgnc_id/HGNC:6016, http://www.uniprot.org/uniprot/P05113)	http://www.guidetopharmacology.org/GRAC/LigandDisplayForward?ligandId=4942 (https://www.genenames.org/data/gene‐symbol‐report/#!/hgnc_id/HGNC:2434, http://www.uniprot.org/uniprot/P04141)	–
Antibodies	–	http://www.guidetopharmacology.org/GRAC/LigandDisplayForward?ligandId=7674 (Binding) (p*K*_d_ 8.7) [115]	http://www.guidetopharmacology.org/GRAC/LigandDisplayForward?ligandId=7785 (Binding) (pIC50 9.9) [33]	–

## http://www.guidetopharmacology.org/GRAC/FamilyDisplayForward?familyId=307

### Overview

The IL‐6 receptor family signal through a ternary receptor complex consisting of the cognate receptor and either the IL‐6 signal transducer gp130 (*IL6ST*, http://www.uniprot.org/uniprot/P40189) or the oncostatin M‐specific receptor, β subunit (*OSMR*, http://www.uniprot.org/uniprot/Q99650), which then activates the JAK/STAT, Ras/Raf/MAPK and PI 3‐kinase/PKB signalling modules. Unusually amongst the cytokine receptors, the CNTF receptor is a glycerophosphatidylinositol‐linked protein.

**Table:** 

Nomenclature	http://www.guidetopharmacology.org/GRAC/ObjectDisplayForward?objectId=2310	http://www.guidetopharmacology.org/GRAC/ObjectDisplayForward?objectId=2311	http://www.guidetopharmacology.org/GRAC/ObjectDisplayForward?objectId=2316	http://www.guidetopharmacology.org/GRAC/ObjectDisplayForward?objectId=2312	http://www.guidetopharmacology.org/GRAC/ObjectDisplayForward?objectId=2313	http://www.guidetopharmacology.org/GRAC/ObjectDisplayForward?objectId=2314	http://www.guidetopharmacology.org/GRAC/ObjectDisplayForward?objectId=2315
Subunits	http://www.guidetopharmacology.org/GRAC/ObjectDisplayForward?objectId=1708 (Ligand‐binding subunit), http://www.guidetopharmacology.org/GRAC/ObjectDisplayForward?objectId=2317 (Other subunit)	http://www.guidetopharmacology.org/GRAC/ObjectDisplayForward?objectId=1709 (Ligand‐binding subunit), http://www.guidetopharmacology.org/GRAC/ObjectDisplayForward?objectId=2317 (Other subunit)	http://www.guidetopharmacology.org/GRAC/ObjectDisplayForward?objectId=2318 (Ligand‐binding subunit), http://www.guidetopharmacology.org/GRAC/ObjectDisplayForward?objectId=2317 (Other subunit)	http://www.guidetopharmacology.org/GRAC/ObjectDisplayForward?objectId=1710 (Ligand‐binding subunit), http://www.guidetopharmacology.org/GRAC/ObjectDisplayForward?objectId=1714 (Other subunit)	http://www.guidetopharmacology.org/GRAC/ObjectDisplayForward?objectId=1711 (Ligand‐binding subunit), http://www.guidetopharmacology.org/GRAC/ObjectDisplayForward?objectId=1713 (Other subunit), http://www.guidetopharmacology.org/GRAC/ObjectDisplayForward?objectId=2317	http://www.guidetopharmacology.org/GRAC/ObjectDisplayForward?objectId=1713 (Ligand‐binding subunit), http://www.guidetopharmacology.org/GRAC/ObjectDisplayForward?objectId=2317 (Other subunit)	http://www.guidetopharmacology.org/GRAC/ObjectDisplayForward?objectId=1714 (Ligand‐binding subunit), http://www.guidetopharmacology.org/GRAC/ObjectDisplayForward?objectId=2317 (Other subunit)
Endogenous agonists	http://www.guidetopharmacology.org/GRAC/LigandDisplayForward?ligandId=4998 (https://www.genenames.org/data/gene‐symbol‐report/#!/hgnc_id/HGNC:6018, http://www.uniprot.org/uniprot/P05231) (Murine NIH/3T3 fibroblasts with human IL6R exhibited a single class of binding sites for 125I‐labeled recombinant human interleukin‐6 (125I‐rhIL‐6) (Kd = 440 pM, 20,000 receptors per cell).) [http://www.ncbi.nlm.nih.gov/pubmed/1995637?dopt=AbstractPlus]	http://www.guidetopharmacology.org/GRAC/LigandDisplayForward?ligandId=4976 (https://www.genenames.org/data/gene‐symbol‐report/#!/hgnc_id/HGNC:5966, http://www.uniprot.org/uniprot/P20809)	http://www.guidetopharmacology.org/GRAC/LigandDisplayForward?ligandId=6151 (https://www.genenames.org/data/gene‐symbol‐report/#!/hgnc_id/HGNC:3129 https://www.genenames.org/data/gene‐symbol‐report/#!/hgnc_id/HGNC:19157, http://www.uniprot.org/uniprot/Q14213 http://www.uniprot.org/uniprot/Q14213)	http://www.guidetopharmacology.org/GRAC/LigandDisplayForward?ligandId=4995 (https://www.genenames.org/data/gene‐symbol‐report/#!/hgnc_id/HGNC:19372, http://www.uniprot.org/uniprot/Q6EBC2)	http://www.guidetopharmacology.org/GRAC/LigandDisplayForward?ligandId=6150 (https://www.genenames.org/data/gene‐symbol‐report/#!/hgnc_id/HGNC:17412 https://www.genenames.org/data/gene‐symbol‐report/#!/hgnc_id/HGNC:2364, http://www.uniprot.org/uniprot/O75462 http://www.uniprot.org/uniprot/Q9UBD9), http://www.guidetopharmacology.org/GRAC/LigandDisplayForward?ligandId=4897 (https://www.genenames.org/data/gene‐symbol‐report/#!/hgnc_id/HGNC:2169, http://www.uniprot.org/uniprot/P26441)	http://www.guidetopharmacology.org/GRAC/LigandDisplayForward?ligandId=5016 (https://www.genenames.org/data/gene‐symbol‐report/#!/hgnc_id/HGNC:6596, http://www.uniprot.org/uniprot/P15018), http://www.guidetopharmacology.org/GRAC/LigandDisplayForward?ligandId=4906 (https://www.genenames.org/data/gene‐symbol‐report/#!/hgnc_id/HGNC:2499, http://www.uniprot.org/uniprot/Q16619), http://www.guidetopharmacology.org/GRAC/LigandDisplayForward?ligandId=5035 (https://www.genenames.org/data/gene‐symbol‐report/#!/hgnc_id/HGNC:8506, http://www.uniprot.org/uniprot/P13725)	http://www.guidetopharmacology.org/GRAC/LigandDisplayForward?ligandId=5035(https://www.genenames.org/data/gene‐symbol‐report/#!/hgnc_id/HGNC:8506, http://www.uniprot.org/uniprot/P13725)
Agonists	–	http://www.guidetopharmacology.org/GRAC/LigandDisplayForward?ligandId=6971 [http://www.ncbi.nlm.nih.gov/pubmed/11033834?dopt=AbstractPlus, http://www.ncbi.nlm.nih.gov/pubmed/18020592?dopt=AbstractPlus]	–	–	–	–	–
Antibodies	http://www.guidetopharmacology.org/GRAC/LigandDisplayForward?ligandId=8363 (Binding) (p*K*_d_ 12.7) [http://www.ncbi.nlm.nih.gov/pubmed/25994180?dopt=AbstractPlus], http://www.guidetopharmacology.org/GRAC/LigandDisplayForward?ligandId=9093 (Binding) (p*K*_d_ 8.9) [97], http://www.guidetopharmacology.org/GRAC/LigandDisplayForward?ligandId=6881 (Binding) (p*K*_d_ 8.6)	–	–	–	–	–	–

### Subunits

**Table:** 

Nomenclature	http://www.guidetopharmacology.org/GRAC/ObjectDisplayForward?objectId=1708	http://www.guidetopharmacology.org/GRAC/ObjectDisplayForward?objectId=2317
Systematic nomenclature	interleukin 6 receptor	interleukin 6 signal transducer
Common abbreviation	IL6R	IL6ST
HGNC, UniProt	https://www.genenames.org/data/gene‐symbol‐report/#!/hgnc_id/HGNC:6019, http://www.uniprot.org/uniprot/P08887	https://www.genenames.org/data/gene‐symbol‐report/#!/hgnc_id/HGNC:6021, http://www.uniprot.org/uniprot/P40189
Endogenous agonists	http://www.guidetopharmacology.org/GRAC/LigandDisplayForward?ligandId=4998 (https://www.genenames.org/data/gene‐symbol‐report/#!/hgnc_id/HGNC:6018, http://www.uniprot.org/uniprot/P05231) (IL6R) expressed stably in murine NIH/3T3 fibroblasts.exhibited a single class of binding sites for 125I‐labeled recombinant human interleukin‐6 (125I‐rhIL‐6) (Kd = 440 pM, 20,000 receptors per cell).) [http://www.ncbi.nlm.nih.gov/pubmed/1995637?dopt=AbstractPlus]	–
Antibodies	http://www.guidetopharmacology.org/GRAC/LigandDisplayForward?ligandId=7999 (Binding) (p*K*_d_ 10.6–11.1) [205]	–

**Table:** 

Nomenclature	http://www.guidetopharmacology.org/GRAC/ObjectDisplayForward?objectId=1709	http://www.guidetopharmacology.org/GRAC/ObjectDisplayForward?objectId=2318	http://www.guidetopharmacology.org/GRAC/ObjectDisplayForward?objectId=1710	http://www.guidetopharmacology.org/GRAC/ObjectDisplayForward?objectId=1711	http://www.guidetopharmacology.org/GRAC/ObjectDisplayForward?objectId=1712	http://www.guidetopharmacology.org/GRAC/ObjectDisplayForward?objectId=1713	http://www.guidetopharmacology.org/GRAC/ObjectDisplayForward?objectId=1714
HGNC, UniProt	https://www.genenames.org/data/gene‐symbol‐report/#!/hgnc_id/HGNC:5967, http://www.uniprot.org/uniprot/Q14626	https://www.genenames.org/data/gene‐symbol‐report/#!/hgnc_id/HGNC:17290, http://www.uniprot.org/uniprot/Q6UWB1	https://www.genenames.org/data/gene‐symbol‐report/#!/hgnc_id/HGNC:18969, http://www.uniprot.org/uniprot/Q8NI17	https://www.genenames.org/data/gene‐symbol‐report/#!/hgnc_id/HGNC:2170, http://www.uniprot.org/uniprot/P26992	https://www.genenames.org/data/gene‐symbol‐report/#!/hgnc_id/HGNC:6554, http://www.uniprot.org/uniprot/P48357	https://www.genenames.org/data/gene‐symbol‐report/#!/hgnc_id/HGNC:6597, http://www.uniprot.org/uniprot/P42702	https://www.genenames.org/data/gene‐symbol‐report/#!/hgnc_id/HGNC:8507, http://www.uniprot.org/uniprot/Q99650
Endogenous agonists	–	–	–	–	http://www.guidetopharmacology.org/GRAC/LigandDisplayForward?ligandId=5015 (https://www.genenames.org/data/gene‐symbol‐report/#!/hgnc_id/HGNC:6553, http://www.uniprot.org/uniprot/P41159) [http://www.ncbi.nlm.nih.gov/pubmed/24743494?dopt=AbstractPlus] – Mouse	–	–

### Further reading on IL‐6 receptor family

Ho LJ *et al*. (2015) Biological effects of interleukin‐6: Clinical applications in autoimmune diseases and cancers. *Biochem. Pharmacol*. **97**: 16‐26 https://www.ncbi.nlm.nih.gov/pubmed/26080005?dopt=AbstractPlus

Kang S *et al*. (2019) Targeting Interleukin‐6 Signaling in Clinic *Immunity* **50**: 1007‐1023

Murakami M *et al*. (2019) Pleiotropy and Specificity: Insights from the Interleukin 6 Family of Cytokines *Immunity* **50**: 812‐831

Rothaug M *et al*. (2016) The role of interleukin‐6 signaling in nervous tissue. *Biochim. Biophys. Acta* **1863**: 1218‐27 https://www.ncbi.nlm.nih.gov/pubmed/27016501?dopt=AbstractPlus

## http://www.guidetopharmacology.org/GRAC/FamilyDisplayForward?familyId=308

### Overview

IL‐12 receptors are a subfamily of the IL‐6 receptor family. IL12RB1 is shared between receptors for IL‐12 and IL‐23; the functional agonist at IL‐12 receptors is a heterodimer of IL‐12A/IL‐12B, while that for IL‐23 receptors is a heterodimer of IL‐12B/IL‐23A.

**Table:** 

Nomenclature	http://www.guidetopharmacology.org/GRAC/ObjectDisplayForward?objectId=2292	http://www.guidetopharmacology.org/GRAC/ObjectDisplayForward?objectId=2293
Subunits	http://www.guidetopharmacology.org/GRAC/ObjectDisplayForward?objectId=1715 (Ligand‐binding subunit), http://www.guidetopharmacology.org/GRAC/ObjectDisplayForward?objectId=1716 (Other subunit)	http://www.guidetopharmacology.org/GRAC/ObjectDisplayForward?objectId=1717 (Ligand‐binding subunit), http://www.guidetopharmacology.org/GRAC/ObjectDisplayForward?objectId=1715 (Ligand‐binding subunit)
Endogenous agonists	http://www.guidetopharmacology.org/GRAC/LigandDisplayForward?ligandId=4977 (https://www.genenames.org/data/gene‐symbol‐report/#!/hgnc_id/HGNC:5969 https://www.genenames.org/data/gene‐symbol‐report/#!/hgnc_id/HGNC:5970, http://www.uniprot.org/uniprot/P29459 http://www.uniprot.org/uniprot/P29460)	http://www.guidetopharmacology.org/GRAC/LigandDisplayForward?ligandId=4978 (https://www.genenames.org/data/gene‐symbol‐report/#!/hgnc_id/HGNC:5970 https://www.genenames.org/data/gene‐symbol‐report/#!/hgnc_id/HGNC:15488, http://www.uniprot.org/uniprot/P29460)

### Subunits

**Table:** 

Nomenclature	http://www.guidetopharmacology.org/GRAC/ObjectDisplayForward?objectId=1715	http://www.guidetopharmacology.org/GRAC/ObjectDisplayForward?objectId=1716	http://www.guidetopharmacology.org/GRAC/ObjectDisplayForward?objectId=1717
HGNC, UniProt	https://www.genenames.org/data/gene‐symbol‐report/#!/hgnc_id/HGNC:5971, http://www.uniprot.org/uniprot/P42701	https://www.genenames.org/data/gene‐symbol‐report/#!/hgnc_id/HGNC:5972, http://www.uniprot.org/uniprot/Q99665	https://www.genenames.org/data/gene‐symbol‐report/#!/hgnc_id/HGNC:19100, http://www.uniprot.org/uniprot/Q5VWK5

### Further reading on IL‐12 receptor family

Wojno EDT *et al*. (2019) The Immunobiology of the Interleukin‐12 Family: Room for Discovery *Immunity* **50**: 851‐870

## http://www.guidetopharmacology.org/GRAC/FamilyDisplayForward?familyId=309

### Overview

Prolactin family receptors form homodimers in the presence of their respective ligands, associate exclusively with Jak2 and signal via Stat5.

**Table:** 

Nomenclature	http://www.guidetopharmacology.org/GRAC/ObjectDisplayForward?objectId=1718	http://www.guidetopharmacology.org/GRAC/ObjectDisplayForward?objectId=1719	http://www.guidetopharmacology.org/GRAC/ObjectDisplayForward?objectId=1720	http://www.guidetopharmacology.org/GRAC/ObjectDisplayForward?objectId=1721	http://www.guidetopharmacology.org/GRAC/ObjectDisplayForward?objectId=1722
HGNC, UniProt	https://www.genenames.org/data/gene‐symbol‐report/#!/hgnc_id/HGNC:3416, http://www.uniprot.org/uniprot/P19235	https://www.genenames.org/data/gene‐symbol‐report/#!/hgnc_id/HGNC:2439, http://www.uniprot.org/uniprot/Q99062	https://www.genenames.org/data/gene‐symbol‐report/#!/hgnc_id/HGNC:4263, http://www.uniprot.org/uniprot/P10912	https://www.genenames.org/data/gene‐symbol‐report/#!/hgnc_id/HGNC:9446, http://www.uniprot.org/uniprot/P16471	https://www.genenames.org/data/gene‐symbol‐report/#!/hgnc_id/HGNC:7217, http://www.uniprot.org/uniprot/P40238
Endogenous agonists	http://www.guidetopharmacology.org/GRAC/LigandDisplayForward?ligandId=4921 (https://www.genenames.org/data/gene‐symbol‐report/#!/hgnc_id/HGNC:3415, http://www.uniprot.org/uniprot/P01588) [http://www.ncbi.nlm.nih.gov/pubmed/16982323?dopt=AbstractPlus]	http://www.guidetopharmacology.org/GRAC/LigandDisplayForward?ligandId=4934 (https://www.genenames.org/data/gene‐symbol‐report/#!/hgnc_id/HGNC:2438, http://www.uniprot.org/uniprot/P09919)	http://www.guidetopharmacology.org/GRAC/LigandDisplayForward?ligandId=4943 (https://www.genenames.org/data/gene‐symbol‐report/#!/hgnc_id/HGNC:4261, http://www.uniprot.org/uniprot/P01241), http://www.guidetopharmacology.org/GRAC/LigandDisplayForward?ligandId=4944 (https://www.genenames.org/data/gene‐symbol‐report/#!/hgnc_id/HGNC:4262, http://www.uniprot.org/uniprot/P01242)	http://www.guidetopharmacology.org/GRAC/LigandDisplayForward?ligandId=5049 (https://www.genenames.org/data/gene‐symbol‐report/#!/hgnc_id/HGNC:9445, http://www.uniprot.org/uniprot/P01236) [http://www.ncbi.nlm.nih.gov/pubmed/4362846?dopt=AbstractPlus] – Mouse, http://www.guidetopharmacology.org/GRAC/LigandDisplayForward?ligandId=4893 (https://www.genenames.org/data/gene‐symbol‐report/#!/hgnc_id/HGNC:2440 https://www.genenames.org/data/gene‐symbol‐report/#!/hgnc_id/HGNC:2441, http://www.uniprot.org/uniprot/P01243), http://www.guidetopharmacology.org/GRAC/LigandDisplayForward?ligandId=4894 (https://www.genenames.org/data/gene‐symbol‐report/#!/hgnc_id/HGNC:2442, http://www.uniprot.org/uniprot/Q14406)	http://www.guidetopharmacology.org/GRAC/LigandDisplayForward?ligandId=5063 (https://www.genenames.org/data/gene‐symbol‐report/#!/hgnc_id/HGNC:11795, http://www.uniprot.org/uniprot/P40225)
Agonists	http://www.guidetopharmacology.org/GRAC/LigandDisplayForward?ligandId=7447 [http://www.ncbi.nlm.nih.gov/pubmed/16982323?dopt=AbstractPlus]	http://www.guidetopharmacology.org/GRAC/LigandDisplayForward?ligandId=6969	–	–	http://www.guidetopharmacology.org/GRAC/LigandDisplayForward?ligandId=6974
Selective agonists	–	–	–	–	http://www.guidetopharmacology.org/GRAC/LigandDisplayForward?ligandId=6961 [http://www.ncbi.nlm.nih.gov/pubmed/18783949?dopt=AbstractPlus]
Antagonists	–	–	http://www.guidetopharmacology.org/GRAC/LigandDisplayForward?ligandId=7485 [http://www.ncbi.nlm.nih.gov/pubmed/10770982?dopt=AbstractPlus]	–	–

### Further reading on Prolactin receptor family

Cabrera‐Reyes EA *et al*. (2017) Prolactin function and putative expression in the brain. *Endocrine* **57**: 199‐213 https://www.ncbi.nlm.nih.gov/pubmed/28634745?dopt=AbstractPlus

Goffin V. (2017) Prolactin receptor targeting in breast and prostate cancers: New insights into an old challenge. *Pharmacol. Ther*. **179**: 111‐126 https://www.ncbi.nlm.nih.gov/pubmed/28549597?dopt=AbstractPlus

## http://www.guidetopharmacology.org/GRAC/FamilyDisplayForward?familyId=310

### Overview

The interferon receptor family includes receptors for type I (α, β κ and ω) and type II (γ) interferons. There are at least 13 different genes encoding IFN‐α subunits in a cluster on human chromosome 9p22: α1 (*IFNA1*, http://www.uniprot.org/uniprot/P01562), α2 (*IFNA2*, http://www.uniprot.org/uniprot/P01563), α4 (*IFNA4*, http://www.uniprot.org/uniprot/P05014), α5 (*IFNA5*, http://www.uniprot.org/uniprot/P01569), α6 (*IFNA6*, http://www.uniprot.org/uniprot/P05013), α7 (*IFNA7*, http://www.uniprot.org/uniprot/P01567), α8 (*IFNA8*, http://www.uniprot.org/uniprot/P32881), α10 (*IFNA10*, http://www.uniprot.org/uniprot/P01566), α13 (*IFNA13*, http://www.uniprot.org/uniprot/P01562), α14 (*IFNA14*, http://www.uniprot.org/uniprot/P01570), α16 (*IFNA16*, http://www.uniprot.org/uniprot/P05015), α17 (*IFNA17*, http://www.uniprot.org/uniprot/P01571) and α21 (*IFNA21*, http://www.uniprot.org/uniprot/P01568).

**Table:** 

Nomenclature	http://www.guidetopharmacology.org/GRAC/ObjectDisplayForward?objectId=1898	http://www.guidetopharmacology.org/GRAC/ObjectDisplayForward?objectId=1899
Subunits	http://www.guidetopharmacology.org/GRAC/ObjectDisplayForward?objectId=1723 (Ligand‐binding subunit), http://www.guidetopharmacology.org/GRAC/ObjectDisplayForward?objectId=1724 (Other subunit)	http://www.guidetopharmacology.org/GRAC/ObjectDisplayForward?objectId=1725 (Ligand‐binding subunit), http://www.guidetopharmacology.org/GRAC/ObjectDisplayForward?objectId=1726 (Other subunit)
Endogenous agonists	http://www.guidetopharmacology.org/GRAC/LigandDisplayForward?ligandId=4955 (https://www.genenames.org/data/gene‐symbol‐report/#!/hgnc_id/HGNC:5417 https://www.genenames.org/data/gene‐symbol‐report/#!/hgnc_id/HGNC:5419, http://www.uniprot.org/uniprot/P01562), http://www.guidetopharmacology.org/GRAC/LigandDisplayForward?ligandId=4956 (https://www.genenames.org/data/gene‐symbol‐report/#!/hgnc_id/HGNC:5418, http://www.uniprot.org/uniprot/P01566), http://www.guidetopharmacology.org/GRAC/LigandDisplayForward?ligandId=4957 (https://www.genenames.org/data/gene‐symbol‐report/#!/hgnc_id/HGNC:5420, http://www.uniprot.org/uniprot/P01570), http://www.guidetopharmacology.org/GRAC/LigandDisplayForward?ligandId=4958 (https://www.genenames.org/data/gene‐symbol‐report/#!/hgnc_id/HGNC:5421, http://www.uniprot.org/uniprot/P05015), http://www.guidetopharmacology.org/GRAC/LigandDisplayForward?ligandId=4959 (https://www.genenames.org/data/gene‐symbol‐report/#!/hgnc_id/HGNC:5422, http://www.uniprot.org/uniprot/P01571), http://www.guidetopharmacology.org/GRAC/LigandDisplayForward?ligandId=4960 (https://www.genenames.org/data/gene‐symbol‐report/#!/hgnc_id/HGNC:5423, http://www.uniprot.org/uniprot/P01563), http://www.guidetopharmacology.org/GRAC/LigandDisplayForward?ligandId=4961 (https://www.genenames.org/data/gene‐symbol‐report/#!/hgnc_id/HGNC:5424, http://www.uniprot.org/uniprot/P01568), http://www.guidetopharmacology.org/GRAC/LigandDisplayForward?ligandId=4962 (https://www.genenames.org/data/gene‐symbol‐report/#!/hgnc_id/HGNC:5425, http://www.uniprot.org/uniprot/P05014), http://www.guidetopharmacology.org/GRAC/LigandDisplayForward?ligandId=4963 (https://www.genenames.org/data/gene‐symbol‐report/#!/hgnc_id/HGNC:5426, http://www.uniprot.org/uniprot/P01569), http://www.guidetopharmacology.org/GRAC/LigandDisplayForward?ligandId=4964 (https://www.genenames.org/data/gene‐symbol‐report/#!/hgnc_id/HGNC:5427, http://www.uniprot.org/uniprot/P05013), http://www.guidetopharmacology.org/GRAC/LigandDisplayForward?ligandId=4965 (https://www.genenames.org/data/gene‐symbol‐report/#!/hgnc_id/HGNC:5428, http://www.uniprot.org/uniprot/P01567), http://www.guidetopharmacology.org/GRAC/LigandDisplayForward?ligandId=4966 (https://www.genenames.org/data/gene‐symbol‐report/#!/hgnc_id/HGNC:5429, http://www.uniprot.org/uniprot/P32881), http://www.guidetopharmacology.org/GRAC/LigandDisplayForward?ligandId=4967 (https://www.genenames.org/data/gene‐symbol‐report/#!/hgnc_id/HGNC:5434, http://www.uniprot.org/uniprot/P01574), http://www.guidetopharmacology.org/GRAC/LigandDisplayForward?ligandId=4969 (https://www.genenames.org/data/gene‐symbol‐report/#!/hgnc_id/HGNC:21714, http://www.uniprot.org/uniprot/Q9P0W0), http://www.guidetopharmacology.org/GRAC/LigandDisplayForward?ligandId=4970 (https://www.genenames.org/data/gene‐symbol‐report/#!/hgnc_id/HGNC:5448, http://www.uniprot.org/uniprot/P05000)	http://www.guidetopharmacology.org/GRAC/LigandDisplayForward?ligandId=4968 (https://www.genenames.org/data/gene‐symbol‐report/#!/hgnc_id/HGNC:5438, http://www.uniprot.org/uniprot/P01579)
Selective agonists	http://www.guidetopharmacology.org/GRAC/LigandDisplayForward?ligandId=7462 [http://www.ncbi.nlm.nih.gov/pubmed/10607680?dopt=AbstractPlus]	–

### Subunits

**Table:** 

Nomenclature	http://www.guidetopharmacology.org/GRAC/ObjectDisplayForward?objectId=1723	http://www.guidetopharmacology.org/GRAC/ObjectDisplayForward?objectId=1724	http://www.guidetopharmacology.org/GRAC/ObjectDisplayForward?objectId=1725	http://www.guidetopharmacology.org/GRAC/ObjectDisplayForward?objectId=1726
HGNC, UniProt	https://www.genenames.org/data/gene‐symbol‐report/#!/hgnc_id/HGNC:5432, http://www.uniprot.org/uniprot/P17181	https://www.genenames.org/data/gene‐symbol‐report/#!/hgnc_id/HGNC:5433, http://www.uniprot.org/uniprot/P48551	https://www.genenames.org/data/gene‐symbol‐report/#!/hgnc_id/HGNC:5439, http://www.uniprot.org/uniprot/P15260	https://www.genenames.org/data/gene‐symbol‐report/#!/hgnc_id/HGNC:5440, http://www.uniprot.org/uniprot/P38484
Endogenous agonists	http://www.guidetopharmacology.org/GRAC/LigandDisplayForward?ligandId=4967 (https://www.genenames.org/data/gene‐symbol‐report/#!/hgnc_id/HGNC:5434, http://www.uniprot.org/uniprot/P01574) [http://www.ncbi.nlm.nih.gov/pubmed/29311663?dopt=AbstractPlus]	–	–	–
Selective agonists	http://www.guidetopharmacology.org/GRAC/LigandDisplayForward?ligandId=7462 [http://www.ncbi.nlm.nih.gov/pubmed/10607680?dopt=AbstractPlus]	–	–	–
Antibodies	http://www.guidetopharmacology.org/GRAC/LigandDisplayForward?ligandId=8258 (Binding) (p*K*_d_ >10) [26]	–	–	–

### Further reading on Interferon receptor family

Kotenko SV *et al*. (2017) Contribution of type III interferons to antiviral immunity: location, location, location. *J. Biol. Chem*. **292**: 7295‐7303 https://www.ncbi.nlm.nih.gov/pubmed/28289095?dopt=AbstractPlus

Lazear HM *et al*. (2019) Shared and Distinct Functions of Type I and Type III Interferons *Immunity* **50**: 907‐923

Ng CT *et al*. (2016) Alpha and Beta Type 1 Interferon Signaling: Passage for Diverse Biologic Outcomes. *Cell* **164**: 349‐52 https://www.ncbi.nlm.nih.gov/pubmed/26824652?dopt=AbstractPlus

Schreiber G. (2017) The molecular basis for differential type I interferon signaling. *J. Biol. Chem*. **292**: 7285‐7294 https://www.ncbi.nlm.nih.gov/pubmed/28289098?dopt=AbstractPlus

## http://www.guidetopharmacology.org/GRAC/FamilyDisplayForward?familyId=311

### Overview

The IL‐10 family of receptors are heterodimeric combinations of family members: IL10RA/IL10RB responds to IL‐10; IL20RA/IL20RB responds to IL‐19, IL‐20 and IL‐24; IL22RA1/IL20RB responds to IL‐20 and IL‐24; IL22RA1/IL10RB responds to IL‐22; IFNLR1(previouly known as IL28RA)/IL10RB responds to IFN‐λ1, ‐λ2 and ‐λ3 (previouly known as IL‐29, IL‐28A and IL‐28B respectively).

**Table:** 

Nomenclature	http://www.guidetopharmacology.org/GRAC/ObjectDisplayForward?objectId=1900	http://www.guidetopharmacology.org/GRAC/ObjectDisplayForward?objectId=1901	http://www.guidetopharmacology.org/GRAC/ObjectDisplayForward?objectId=1902	http://www.guidetopharmacology.org/GRAC/ObjectDisplayForward?objectId=1903	http://www.guidetopharmacology.org/GRAC/ObjectDisplayForward?objectId=1732	http://www.guidetopharmacology.org/GRAC/ObjectDisplayForward?objectId=1904
HGNC, UniProt	–	–	–	–	https://www.genenames.org/data/gene‐symbol‐report/#!/hgnc_id/HGNC:14901, http://www.uniprot.org/uniprot/Q969J5	–
Subunits	http://www.guidetopharmacology.org/GRAC/ObjectDisplayForward?objectId=1727 (Ligand‐binding subunit), http://www.guidetopharmacology.org/GRAC/ObjectDisplayForward?objectId=1728 (Other subunit)	http://www.guidetopharmacology.org/GRAC/ObjectDisplayForward?objectId=1729 (Ligand‐binding subunit), http://www.guidetopharmacology.org/GRAC/ObjectDisplayForward?objectId=1730 (Other subunit)	http://www.guidetopharmacology.org/GRAC/ObjectDisplayForward?objectId=1731 (Ligand‐binding subunit), http://www.guidetopharmacology.org/GRAC/ObjectDisplayForward?objectId=1730 (Ligand‐binding subunit)	http://www.guidetopharmacology.org/GRAC/ObjectDisplayForward?objectId=1731 (Ligand‐binding subunit), http://www.guidetopharmacology.org/GRAC/ObjectDisplayForward?objectId=1728 (Ligand‐binding subunit)	–	http://www.guidetopharmacology.org/GRAC/ObjectDisplayForward?objectId=1733 (Ligand‐binding subunit), http://www.guidetopharmacology.org/GRAC/ObjectDisplayForward?objectId=1728 (Other subunit)
Endogenous agonists	http://www.guidetopharmacology.org/GRAC/LigandDisplayForward?ligandId=4975 (https://www.genenames.org/data/gene‐symbol‐report/#!/hgnc_id/HGNC:5962, http://www.uniprot.org/uniprot/P22301)	http://www.guidetopharmacology.org/GRAC/LigandDisplayForward?ligandId=4984 (https://www.genenames.org/data/gene‐symbol‐report/#!/hgnc_id/HGNC:5990, http://www.uniprot.org/uniprot/Q9UHD0), http://www.guidetopharmacology.org/GRAC/LigandDisplayForward?ligandId=4986 (https://www.genenames.org/data/gene‐symbol‐report/#!/hgnc_id/HGNC:6002, http://www.uniprot.org/uniprot/Q9NYY1), http://www.guidetopharmacology.org/GRAC/LigandDisplayForward?ligandId=4990 (https://www.genenames.org/data/gene‐symbol‐report/#!/hgnc_id/HGNC:11346, http://www.uniprot.org/uniprot/Q13007)	http://www.guidetopharmacology.org/GRAC/LigandDisplayForward?ligandId=4986 (https://www.genenames.org/data/gene‐symbol‐report/#!/hgnc_id/HGNC:6002, http://www.uniprot.org/uniprot/Q9NYY1), http://www.guidetopharmacology.org/GRAC/LigandDisplayForward?ligandId=4990 (https://www.genenames.org/data/gene‐symbol‐report/#!/hgnc_id/HGNC:11346, http://www.uniprot.org/uniprot/Q13007)	http://www.guidetopharmacology.org/GRAC/LigandDisplayForward?ligandId=4988 (https://www.genenames.org/data/gene‐symbol‐report/#!/hgnc_id/HGNC:14900, http://www.uniprot.org/uniprot/Q9GZX6)	–	http://www.guidetopharmacology.org/GRAC/LigandDisplayForward?ligandId=4993 (https://www.genenames.org/data/gene‐symbol‐report/#!/hgnc_id/HGNC:18363, http://www.uniprot.org/uniprot/Q8IU54), http://www.guidetopharmacology.org/GRAC/LigandDisplayForward?ligandId=4991 (https://www.genenames.org/data/gene‐symbol‐report/#!/hgnc_id/HGNC:18364, http://www.uniprot.org/uniprot/Q8IZJ0), http://www.guidetopharmacology.org/GRAC/LigandDisplayForward?ligandId=4992 (https://www.genenames.org/data/gene‐symbol‐report/#!/hgnc_id/HGNC:18365, http://www.uniprot.org/uniprot/Q8IZI9)
Comments	–	–	–	–	Soluble decoy receptor that binds http://www.guidetopharmacology.org/GRAC/LigandDisplayForward?ligandId=4988 (https://www.genenames.org/data/gene‐symbol‐report/#!/hgnc_id/HGNC:14900, http://www.uniprot.org/uniprot/Q9GZX6) as a monomer.	–

### Subunits

**Table:** 

Nomenclature	http://www.guidetopharmacology.org/GRAC/ObjectDisplayForward?objectId=1727	http://www.guidetopharmacology.org/GRAC/ObjectDisplayForward?objectId=1728	http://www.guidetopharmacology.org/GRAC/ObjectDisplayForward?objectId=1729	http://www.guidetopharmacology.org/GRAC/ObjectDisplayForward?objectId=1730	http://www.guidetopharmacology.org/GRAC/ObjectDisplayForward?objectId=1731	http://www.guidetopharmacology.org/GRAC/ObjectDisplayForward?objectId=1733
HGNC, UniProt	https://www.genenames.org/data/gene‐symbol‐report/#!/hgnc_id/HGNC:5964, http://www.uniprot.org/uniprot/Q13651	https://www.genenames.org/data/gene‐symbol‐report/#!/hgnc_id/HGNC:5965, http://www.uniprot.org/uniprot/Q08334	https://www.genenames.org/data/gene‐symbol‐report/#!/hgnc_id/HGNC:6003, http://www.uniprot.org/uniprot/Q9UHF4	https://www.genenames.org/data/gene‐symbol‐report/#!/hgnc_id/HGNC:6004, http://www.uniprot.org/uniprot/Q6UXL0	https://www.genenames.org/data/gene‐symbol‐report/#!/hgnc_id/HGNC:13700, http://www.uniprot.org/uniprot/Q8N6P7	https://www.genenames.org/data/gene‐symbol‐report/#!/hgnc_id/HGNC:18584, http://www.uniprot.org/uniprot/Q8IU57

### Further reading on IL‐10 receptor family

Felix J *et al*. (2017) Mechanisms of immunomodulation by mammalian and viral decoy receptors: insights from structures. *Nat. Rev. Immunol*. **17**: 112‐129 https://www.ncbi.nlm.nih.gov/pubmed/28028310?dopt=AbstractPlus

Ouyang W *et al*. (2019) IL‐10 Family Cytokines IL‐10 and IL‐22: from Basic Science to Clinical Translation *Immunity* **50**: 871‐891

## http://www.guidetopharmacology.org/GRAC/FamilyDisplayForward?familyId=312

### Overview

The immunoglobulin‐like family of IL‐1 receptors are heterodimeric receptors made up of a cognate receptor subunit and an IL‐1 receptor accessory protein, https://www.genenames.org/data/gene‐symbol‐report/#!/hgnc_id/HGNC:5995 (http://www.uniprot.org/uniprot/Q9NPH3, also known as C3orf13, IL‐1RAcP, IL1R3). They are characterised by extracellular immunoglobulin‐like domains and an intracellular Toll/Interleukin‐1R (TIR) domain.

**Table:** 

Nomenclature	http://www.guidetopharmacology.org/GRAC/ObjectDisplayForward?objectId=1905	http://www.guidetopharmacology.org/GRAC/ObjectDisplayForward?objectId=1906	http://www.guidetopharmacology.org/GRAC/ObjectDisplayForward?objectId=1907	http://www.guidetopharmacology.org/GRAC/ObjectDisplayForward?objectId=2321	http://www.guidetopharmacology.org/GRAC/ObjectDisplayForward?objectId=1908
Subunits	http://www.guidetopharmacology.org/GRAC/ObjectDisplayForward?objectId=1734 (Ligand‐binding subunit), http://www.guidetopharmacology.org/GRAC/ObjectDisplayForward?objectId=1897 (Other subunit)	http://www.guidetopharmacology.org/GRAC/ObjectDisplayForward?objectId=1735 (Ligand‐binding subunit), http://www.guidetopharmacology.org/GRAC/ObjectDisplayForward?objectId=1897 (Other subunit)	http://www.guidetopharmacology.org/GRAC/ObjectDisplayForward?objectId=1736 (Ligand‐binding subunit), http://www.guidetopharmacology.org/GRAC/ObjectDisplayForward?objectId=1897 (Other subunit)	http://www.guidetopharmacology.org/GRAC/ObjectDisplayForward?objectId=2319 (Ligand‐binding subunit), http://www.guidetopharmacology.org/GRAC/ObjectDisplayForward?objectId=1897 (Other subunit)	http://www.guidetopharmacology.org/GRAC/ObjectDisplayForward?objectId=1737 (Ligand‐binding subunit), http://www.guidetopharmacology.org/GRAC/ObjectDisplayForward?objectId=2320 (Other subunit)
Inhibitors	http://www.guidetopharmacology.org/GRAC/LigandDisplayForward?ligandId=6972 (p*K*_d_ 7.8) [http://www.ncbi.nlm.nih.gov/pubmed/1834644?dopt=AbstractPlus]	–	–	–	–
Endogenous agonists	http://www.guidetopharmacology.org/GRAC/LigandDisplayForward?ligandId=4973 (https://www.genenames.org/data/gene‐symbol‐report/#!/hgnc_id/HGNC:5991, http://www.uniprot.org/uniprot/P01583), http://www.guidetopharmacology.org/GRAC/LigandDisplayForward?ligandId=4974 (https://www.genenames.org/data/gene‐symbol‐report/#!/hgnc_id/HGNC:5992, http://www.uniprot.org/uniprot/P01584)	http://www.guidetopharmacology.org/GRAC/LigandDisplayForward?ligandId=5880 (https://www.genenames.org/data/gene‐symbol‐report/#!/hgnc_id/HGNC:16028, http://www.uniprot.org/uniprot/O95760)	http://www.guidetopharmacology.org/GRAC/LigandDisplayForward?ligandId=5881 (https://www.genenames.org/data/gene‐symbol‐report/#!/hgnc_id/HGNC:15562, http://www.uniprot.org/uniprot/Q9UHA7), http://www.guidetopharmacology.org/GRAC/LigandDisplayForward?ligandId=5882 (https://www.genenames.org/data/gene‐symbol‐report/#!/hgnc_id/HGNC:15564, http://www.uniprot.org/uniprot/Q9NZH7), http://www.guidetopharmacology.org/GRAC/LigandDisplayForward?ligandId=5883 (https://www.genenames.org/data/gene‐symbol‐report/#!/hgnc_id/HGNC:15741, http://www.uniprot.org/uniprot/Q9NZH8)	–	http://www.guidetopharmacology.org/GRAC/LigandDisplayForward?ligandId=4983 (https://www.genenames.org/data/gene‐symbol‐report/#!/hgnc_id/HGNC:5986, http://www.uniprot.org/uniprot/Q14116), http://www.guidetopharmacology.org/GRAC/LigandDisplayForward?ligandId=6149 (https://www.genenames.org/data/gene‐symbol‐report/#!/hgnc_id/HGNC:15563, http://www.uniprot.org/uniprot/Q9NZH6)
Endogenous antagonists	http://www.guidetopharmacology.org/GRAC/LigandDisplayForward?ligandId=5878 (https://www.genenames.org/data/gene‐symbol‐report/#!/hgnc_id/HGNC:6000, http://www.uniprot.org/uniprot/P18510)	–	http://www.guidetopharmacology.org/GRAC/LigandDisplayForward?ligandId=5879 (https://www.genenames.org/data/gene‐symbol‐report/#!/hgnc_id/HGNC:15561, http://www.uniprot.org/uniprot/Q9UBH0)	–	–
Selective antagonists	http://www.guidetopharmacology.org/GRAC/LigandDisplayForward?ligandId=4861 [http://www.ncbi.nlm.nih.gov/pubmed/8940020?dopt=AbstractPlus]	–	–	–	–
Comments	–	–	http://www.guidetopharmacology.org/GRAC/LigandDisplayForward?ligandId=5879 (https://www.genenames.org/data/gene‐symbol‐report/#!/hgnc_id/HGNC:15561, http://www.uniprot.org/uniprot/Q9UBH0) is a highly specific antagonist of the response to http://www.guidetopharmacology.org/GRAC/LigandDisplayForward?ligandId=5883 (https://www.genenames.org/data/gene‐symbol‐report/#!/hgnc_id/HGNC:15741, http://www.uniprot.org/uniprot/Q9NZH8).	Decoy receptor that binds http://www.guidetopharmacology.org/GRAC/LigandDisplayForward?ligandId=4973 (https://www.genenames.org/data/gene‐symbol‐report/#!/hgnc_id/HGNC:5991, http://www.uniprot.org/uniprot/P01583), http://www.guidetopharmacology.org/GRAC/LigandDisplayForward?ligandId=4974 (https://www.genenames.org/data/gene‐symbol‐report/#!/hgnc_id/HGNC:5992, http://www.uniprot.org/uniprot/P01584) and http://www.guidetopharmacology.org/GRAC/LigandDisplayForward?ligandId=5878 (https://www.genenames.org/data/gene‐symbol‐report/#!/hgnc_id/HGNC:6000, http://www.uniprot.org/uniprot/P18510).	–

### Subunits

**Table:** 

Nomenclature	http://www.guidetopharmacology.org/GRAC/ObjectDisplayForward?objectId=1734	http://www.guidetopharmacology.org/GRAC/ObjectDisplayForward?objectId=2319	http://www.guidetopharmacology.org/GRAC/ObjectDisplayForward?objectId=1735	http://www.guidetopharmacology.org/GRAC/ObjectDisplayForward?objectId=1736	http://www.guidetopharmacology.org/GRAC/ObjectDisplayForward?objectId=1737
HGNC, UniProt	https://www.genenames.org/data/gene‐symbol‐report/#!/hgnc_id/HGNC:5993, http://www.uniprot.org/uniprot/P14778	https://www.genenames.org/data/gene‐symbol‐report/#!/hgnc_id/HGNC:5994, http://www.uniprot.org/uniprot/P27930	https://www.genenames.org/data/gene‐symbol‐report/#!/hgnc_id/HGNC:5998, http://www.uniprot.org/uniprot/Q01638	https://www.genenames.org/data/gene‐symbol‐report/#!/hgnc_id/HGNC:5999, http://www.uniprot.org/uniprot/Q9HB29	https://www.genenames.org/data/gene‐symbol‐report/#!/hgnc_id/HGNC:5988, http://www.uniprot.org/uniprot/Q13478

### Further reading on Immunoglobulin‐like family of IL‐1 receptors

Afonina IS *et al*. (2015) Proteolytic Processing of Interleukin‐1 Family Cytokines: Variations on a Common Theme. *Immunity* **42**: 991‐1004 https://www.ncbi.nlm.nih.gov/pubmed/26084020?dopt=AbstractPlus

Mantovani A *et al*. (2019) Interleukin‐1 and Related Cytokines in the Regulation of Inflammation and *Immunity Immunity* **50**: 778‐795

## http://www.guidetopharmacology.org/GRAC/FamilyDisplayForward?familyId=313

### Overview

The IL17 cytokine family consists of six ligands (IL‐17A‐F), which signal through five receptors (IL‐17RA‐E).

**Table:** 

Nomenclature	http://www.guidetopharmacology.org/GRAC/ObjectDisplayForward?objectId=2294	http://www.guidetopharmacology.org/GRAC/ObjectDisplayForward?objectId=2295	http://www.guidetopharmacology.org/GRAC/ObjectDisplayForward?objectId=2296
Subunits	http://www.guidetopharmacology.org/GRAC/ObjectDisplayForward?objectId=1738 (Ligand‐binding subunit), http://www.guidetopharmacology.org/GRAC/ObjectDisplayForward?objectId=1740 (Other subunit)	http://www.guidetopharmacology.org/GRAC/ObjectDisplayForward?objectId=1739 (Ligand‐binding subunit), http://www.guidetopharmacology.org/GRAC/ObjectDisplayForward?objectId=1738 (Other subunit)	http://www.guidetopharmacology.org/GRAC/ObjectDisplayForward?objectId=1742 (Ligand‐binding subunit), http://www.guidetopharmacology.org/GRAC/ObjectDisplayForward?objectId=1738 (Other subunit)
Endogenous agonists	http://www.guidetopharmacology.org/GRAC/LigandDisplayForward?ligandId=4982 (https://www.genenames.org/data/gene‐symbol‐report/#!/hgnc_id/HGNC:5981, http://www.uniprot.org/uniprot/Q16552), http://www.guidetopharmacology.org/GRAC/LigandDisplayForward?ligandId=5874 (https://www.genenames.org/data/gene‐symbol‐report/#!/hgnc_id/HGNC:5981 https://www.genenames.org/data/gene‐symbol‐report/#!/hgnc_id/HGNC:16404, http://www.uniprot.org/uniprot/Q16552 http://www.uniprot.org/uniprot/Q96PD4), http://www.guidetopharmacology.org/GRAC/LigandDisplayForward?ligandId=5873 (https://www.genenames.org/data/gene‐symbol‐report/#!/hgnc_id/HGNC:16404, http://www.uniprot.org/uniprot/Q96PD4)	http://www.guidetopharmacology.org/GRAC/LigandDisplayForward?ligandId=5877 (https://www.genenames.org/data/gene‐symbol‐report/#!/hgnc_id/HGNC:5982, http://www.uniprot.org/uniprot/Q9UHF5), http://www.guidetopharmacology.org/GRAC/LigandDisplayForward?ligandId=5876 (https://www.genenames.org/data/gene‐symbol‐report/#!/hgnc_id/HGNC:13765, http://www.uniprot.org/uniprot/Q9H293)	http://www.guidetopharmacology.org/GRAC/LigandDisplayForward?ligandId=5875 (https://www.genenames.org/data/gene‐symbol‐report/#!/hgnc_id/HGNC:5983, http://www.uniprot.org/uniprot/Q9P0M4)

### Subunits

**Table:** 

Nomenclature	http://www.guidetopharmacology.org/GRAC/ObjectDisplayForward?objectId=1738	http://www.guidetopharmacology.org/GRAC/ObjectDisplayForward?objectId=1739	http://www.guidetopharmacology.org/GRAC/ObjectDisplayForward?objectId=1740	http://www.guidetopharmacology.org/GRAC/ObjectDisplayForward?objectId=1741	http://www.guidetopharmacology.org/GRAC/ObjectDisplayForward?objectId=1742
HGNC, UniProt	https://www.genenames.org/data/gene‐symbol‐report/#!/hgnc_id/HGNC:5985, http://www.uniprot.org/uniprot/Q96F46	https://www.genenames.org/data/gene‐symbol‐report/#!/hgnc_id/HGNC:18015, http://www.uniprot.org/uniprot/Q9NRM6	https://www.genenames.org/data/gene‐symbol‐report/#!/hgnc_id/HGNC:18358, http://www.uniprot.org/uniprot/Q8NAC3	https://www.genenames.org/data/gene‐symbol‐report/#!/hgnc_id/HGNC:17616, http://www.uniprot.org/uniprot/Q8NFM7	https://www.genenames.org/data/gene‐symbol‐report/#!/hgnc_id/HGNC:18439, http://www.uniprot.org/uniprot/Q8NFR9
Antibodies	http://www.guidetopharmacology.org/GRAC/LigandDisplayForward?ligandId=7540 (Binding) (p*K*_d_ 9.2) [213]	–	–	–	–
Comments	–	–	–	The endogenous agonist for this receptor is unknown.	–

### Further reading on IL‐17 receptor family

Beringer A *et al*. (2016) IL‐17 in Chronic Inflammation: From Discovery to Targeting. *Trends Mol Med* **22**: 230‐241 https://www.ncbi.nlm.nih.gov/pubmed/26837266?dopt=AbstractPlus

Lubberts E. (2015) The IL‐23‐IL‐17 axis in inflammatory arthritis. *Nat Rev Rheumatol* **11**: 415‐29 https://www.ncbi.nlm.nih.gov/pubmed/25907700?dopt=AbstractPlus

McGeachy MJ *et al*. (2019) The IL‐17 Family of Cytokines in Health and Disease *Immunity* **50**: 892‐906

## http://www.guidetopharmacology.org/GRAC/FamilyDisplayForward?familyId=314

### Overview

GDNF family receptors (provisional nomenclature) are extrinsic tyrosine kinase receptors. Ligand binding to the extracellular domain of the glycosylphosphatidylinositol‐linked cell‐surface receptors (tabulated below) activates a transmembrane tyrosine kinase enzyme, https://www.genenames.org/data/gene‐symbol‐report/#!/hgnc_id/HGNC:9967 (see http://www.guidetopharmacology.org/GRAC/FamilyDisplayForward?familyId=304). The endogenous ligands are typically dimeric, linked through disulphide bridges: glial cell‐derived neurotrophic factor http://www.guidetopharmacology.org/GRAC/LigandDisplayForward?ligandId=4940 (https://www.genenames.org/data/gene‐symbol‐report/#!/hgnc_id/HGNC:4232, http://www.uniprot.org/uniprot/P39905) (211 aa); http://www.guidetopharmacology.org/GRAC/LigandDisplayForward?ligandId=5032 (https://www.genenames.org/data/gene‐symbol‐report/#!/hgnc_id/HGNC:8007, http://www.uniprot.org/uniprot/Q99748) (197 aa); http://www.guidetopharmacology.org/GRAC/LigandDisplayForward?ligandId=4871 (https://www.genenames.org/data/gene‐symbol‐report/#!/hgnc_id/HGNC:727, http://www.uniprot.org/uniprot/Q5T4W7) (237 aa) and http://www.guidetopharmacology.org/GRAC/LigandDisplayForward?ligandId=5045 (https://www.genenames.org/data/gene‐symbol‐report/#!/hgnc_id/HGNC:9579, http://www.uniprot.org/uniprot/O60542) (PSPN, 156 aa).

**Table:** 

Nomenclature	http://www.guidetopharmacology.org/GRAC/ObjectDisplayForward?objectId=1743	http://www.guidetopharmacology.org/GRAC/ObjectDisplayForward?objectId=1744	http://www.guidetopharmacology.org/GRAC/ObjectDisplayForward?objectId=1745	http://www.guidetopharmacology.org/GRAC/ObjectDisplayForward?objectId=1746
Common abbreviation	GFRα1	GFRα2	GFRα3	GFRα4
HGNC, UniProt	https://www.genenames.org/data/gene‐symbol‐report/#!/hgnc_id/HGNC:4243, http://www.uniprot.org/uniprot/P56159	https://www.genenames.org/data/gene‐symbol‐report/#!/hgnc_id/HGNC:4244, http://www.uniprot.org/uniprot/O00451	https://www.genenames.org/data/gene‐symbol‐report/#!/hgnc_id/HGNC:4245, http://www.uniprot.org/uniprot/O60609	https://www.genenames.org/data/gene‐symbol‐report/#!/hgnc_id/HGNC:13821, http://www.uniprot.org/uniprot/Q9GZZ7
Potency order	http://www.guidetopharmacology.org/GRAC/LigandDisplayForward?ligandId=4940 (https://www.genenames.org/data/gene‐symbol‐report/#!/hgnc_id/HGNC:4232, http://www.uniprot.org/uniprot/P39905) > http://www.guidetopharmacology.org/GRAC/LigandDisplayForward?ligandId=5032 (https://www.genenames.org/data/gene‐symbol‐report/#!/hgnc_id/HGNC:8007, http://www.uniprot.org/uniprot/Q99748) > http://www.guidetopharmacology.org/GRAC/LigandDisplayForward?ligandId=4871 (https://www.genenames.org/data/gene‐symbol‐report/#!/hgnc_id/HGNC:727, http://www.uniprot.org/uniprot/Q5T4W7)	http://www.guidetopharmacology.org/GRAC/LigandDisplayForward?ligandId=5032 (https://www.genenames.org/data/gene‐symbol‐report/#!/hgnc_id/HGNC:8007, http://www.uniprot.org/uniprot/Q99748) > http://www.guidetopharmacology.org/GRAC/LigandDisplayForward?ligandId=4940 (https://www.genenames.org/data/gene‐symbol‐report/#!/hgnc_id/HGNC:4232, http://www.uniprot.org/uniprot/P39905)	http://www.guidetopharmacology.org/GRAC/LigandDisplayForward?ligandId=4871 (https://www.genenames.org/data/gene‐symbol‐report/#!/hgnc_id/HGNC:727, http://www.uniprot.org/uniprot/Q5T4W7)	http://www.guidetopharmacology.org/GRAC/LigandDisplayForward?ligandId=5045 (https://www.genenames.org/data/gene‐symbol‐report/#!/hgnc_id/HGNC:9579, http://www.uniprot.org/uniprot/O60542)
Labelled ligands	http://www.guidetopharmacology.org/GRAC/LigandDisplayForward?ligandId=4851 (p*K*_d_ 10.2–11.5) [http://www.ncbi.nlm.nih.gov/pubmed/9192898?dopt=AbstractPlus, http://www.ncbi.nlm.nih.gov/pubmed/8657309?dopt=AbstractPlus]	–	–	–

### Comments

Inhibitors of other receptor tyrosine kinases, such as http://www.guidetopharmacology.org/GRAC/LigandDisplayForward?ligandId=5056, which inhibits VEGF receptor function, may also inhibit Ret function [http://www.ncbi.nlm.nih.gov/pubmed/17032739?dopt=AbstractPlus]. Mutations of RET and GDNF genes may be involved in Hirschsprung&apos;s disease, which is characterized by the absence of intramural ganglion cells in the hindgut, often resulting in intestinal obstruction.

### Further reading on GDNF receptor family

Allen SJ *et al*. (2013) GDNF, NGF and BDNF as therapeutic options for neurodegeneration. *Pharmacol. Ther*. **138**: 155‐75 https://www.ncbi.nlm.nih.gov/pubmed/23348013?dopt=AbstractPlus

Ibáñez CF *et al*. (2017) Biology of GDNF and its receptors ‐ Relevance for disorders of the central nervous system. *Neurobiol. Dis*. **97**: 80‐89 https://www.ncbi.nlm.nih.gov/pubmed/26829643?dopt=AbstractPlus

Merighi A. (2016) Targeting the glial‐derived neurotrophic factor and related molecules for controlling normal and pathologic pain. *Expert Opin. Ther. Targets* **20**: 193‐208 https://www.ncbi.nlm.nih.gov/pubmed/26863504?dopt=AbstractPlus

## http://www.guidetopharmacology.org/GRAC/FamilyDisplayForward?familyId=760

### Overview

Integrins are unusual signalling proteins that function to signal both from the extracellular environment into the cell, but also from the cytoplasm to the external of the cell. The intracellular signalling cascades associated with integrin activation focus on protein kinase activities, such as focal adhesion kinase and Src. Based on this association between extracellular signals and intracellular protein kinase activity, we have chosen to include integrins in the ‘Catalytic receptors’ section of the database until more stringent criteria from NC‐IUPHAR allows precise definition of their classification.

Integrins are heterodimeric entities, composed of α and β subunits, each 1TM proteins, which bind components of the extracellular matrix or counter‐receptors expressed on other cells. One class of integrin contains an inserted domain (I) in its α subunit, and if present (in α1, α2, α10, α11, αD, αE, αL, αM and αX), this I domain contains the ligand binding site. All β subunits possess a similar I‐like domain, which has the capacity to bind ligand, often recognising the RGD motif. The presence of an a subunit I domain precludes ligand binding through the β subunit. Integrins provide a link between ligand and the actin cytoskeleton (through typically short intracellular domains). Integrins bind several divalent cations, including a Mg^2+^ ion in the I or I‐like domain that is essential for ligand binding. Other cation binding sites may regulate integrin activity or stabilise the 3D structure. Integrins regulate the activity of particular protein kinases, including focal adhesion kinase and integrin‐linked kinase. Cellular activation regulates integrin ligand affinity via inside‐out signalling and ligand binding to integrins can regulate cellular activity *via* outsidein signalling.

Several drugs that target integrins are in clinical use including: (1) http://www.guidetopharmacology.org/GRAC/LigandDisplayForward?ligandId=6584 (αIIbβ3) for short term prevention of coronary thrombosis, (2) http://www.guidetopharmacology.org/GRAC/LigandDisplayForward?ligandId=7437 (α4β7) to reduce gastrointestinal inflammation, and (3) http://www.guidetopharmacology.org/GRAC/LigandDisplayForward?ligandId=6591 (α4β1) in some cases of severe multiple sclerosis.

**Table:** 

Nomenclature	http://www.guidetopharmacology.org/GRAC/ObjectDisplayForward?objectId=2577	http://www.guidetopharmacology.org/GRAC/ObjectDisplayForward?objectId=2578	http://www.guidetopharmacology.org/GRAC/ObjectDisplayForward?objectId=2579	http://www.guidetopharmacology.org/GRAC/ObjectDisplayForward?objectId=2580
Subunits	http://www.guidetopharmacology.org/GRAC/ObjectDisplayForward?objectId=2455, http://www.guidetopharmacology.org/GRAC/ObjectDisplayForward?objectId=2437	http://www.guidetopharmacology.org/GRAC/ObjectDisplayForward?objectId=2455, http://www.guidetopharmacology.org/GRAC/ObjectDisplayForward?objectId=2440	http://www.guidetopharmacology.org/GRAC/ObjectDisplayForward?objectId=2457, http://www.guidetopharmacology.org/GRAC/ObjectDisplayForward?objectId=2441	http://www.guidetopharmacology.org/GRAC/ObjectDisplayForward?objectId=2455, http://www.guidetopharmacology.org/GRAC/ObjectDisplayForward?objectId=2443
Ligands	collagen, laminin	collagen, laminin, thrombospondin	http://www.guidetopharmacology.org/GRAC/LigandDisplayForward?ligandId=6749 (https://www.genenames.org/data/gene‐symbol‐report/#!/hgnc_id/HGNC:3661 https://www.genenames.org/data/gene‐symbol‐report/#!/hgnc_id/HGNC:3662 https://www.genenames.org/data/gene‐symbol‐report/#!/hgnc_id/HGNC:3694, http://www.uniprot.org/uniprot/P02671 http://www.uniprot.org/uniprot/P02675 http://www.uniprot.org/uniprot/P02679), http://www.guidetopharmacology.org/GRAC/LigandDisplayForward?ligandId=6754 (https://www.genenames.org/data/gene‐symbol‐report/#!/hgnc_id/HGNC:3778, http://www.uniprot.org/uniprot/P02751), http://www.guidetopharmacology.org/GRAC/LigandDisplayForward?ligandId=6755 (https://www.genenames.org/data/gene‐symbol‐report/#!/hgnc_id/HGNC:12726, http://www.uniprot.org/uniprot/P04275), http://www.guidetopharmacology.org/GRAC/LigandDisplayForward?ligandId=6746 (https://www.genenames.org/data/gene‐symbol‐report/#!/hgnc_id/HGNC:12724, http://www.uniprot.org/uniprot/P04004), thrombospondin	http://www.guidetopharmacology.org/GRAC/LigandDisplayForward?ligandId=6754 (https://www.genenames.org/data/gene‐symbol‐report/#!/hgnc_id/HGNC:3778, http://www.uniprot.org/uniprot/P02751), http://www.guidetopharmacology.org/GRAC/LigandDisplayForward?ligandId=6758 (https://www.genenames.org/data/gene‐symbol‐report/#!/hgnc_id/HGNC:12663, http://www.uniprot.org/uniprot/P19320), http://www.guidetopharmacology.org/GRAC/LigandDisplayForward?ligandId=6753 (https://www.genenames.org/data/gene‐symbol‐report/#!/hgnc_id/HGNC:11255, http://www.uniprot.org/uniprot/P10451), thrombospondin
Inhibitors	http://www.guidetopharmacology.org/GRAC/LigandDisplayForward?ligandId=6581 (pIC_50_ 9.1) [http://www.ncbi.nlm.nih.gov/pubmed/12727812?dopt=AbstractPlus]	http://www.guidetopharmacology.org/GRAC/LigandDisplayForward?ligandId=6582 (pIC_50_ 7.9) [http://www.ncbi.nlm.nih.gov/pubmed/19141632?dopt=AbstractPlus]	http://www.guidetopharmacology.org/GRAC/LigandDisplayForward?ligandId=6586 (pIC_50_ 9.4) [http://www.ncbi.nlm.nih.gov/pubmed/23644213?dopt=AbstractPlus], http://www.guidetopharmacology.org/GRAC/LigandDisplayForward?ligandId=6588 (p*K*_i_ 8.4) [http://www.ncbi.nlm.nih.gov/pubmed/7955174?dopt=AbstractPlus, http://www.ncbi.nlm.nih.gov/pubmed/8485125?dopt=AbstractPlus], http://www.guidetopharmacology.org/GRAC/LigandDisplayForward?ligandId=6587 (pIC_50_ 7.4) [http://www.ncbi.nlm.nih.gov/pubmed/7966149?dopt=AbstractPlus], http://www.guidetopharmacology.org/GRAC/LigandDisplayForward?ligandId=6585 (pIC_50_ 6.2–6.8) [http://www.ncbi.nlm.nih.gov/pubmed/10999999?dopt=AbstractPlus]	http://www.guidetopharmacology.org/GRAC/LigandDisplayForward?ligandId=6589 (pIC_50_ 8.3–9) [http://www.ncbi.nlm.nih.gov/pubmed/10072689?dopt=AbstractPlus], http://www.guidetopharmacology.org/GRAC/LigandDisplayForward?ligandId=6590
Antibodies	–	–	http://www.guidetopharmacology.org/GRAC/LigandDisplayForward?ligandId=6584 (Binding) [35]	http://www.guidetopharmacology.org/GRAC/LigandDisplayForward?ligandId=6591 (Inhibition) [http://www.ncbi.nlm.nih.gov/pubmed/15293871?dopt=AbstractPlus]
Comments	–	–	–	http://www.guidetopharmacology.org/GRAC/LigandDisplayForward?ligandId=6759 is used as a probe at this receptor.

**Table:** 

Nomenclature	http://www.guidetopharmacology.org/GRAC/ObjectDisplayForward?objectId=2770	http://www.guidetopharmacology.org/GRAC/ObjectDisplayForward?objectId=2581	http://www.guidetopharmacology.org/GRAC/ObjectDisplayForward?objectId=2867	http://www.guidetopharmacology.org/GRAC/ObjectDisplayForward?objectId=2868
Subunits	http://www.guidetopharmacology.org/GRAC/ObjectDisplayForward?objectId=2443, http://www.guidetopharmacology.org/GRAC/ObjectDisplayForward?objectId=2461	http://www.guidetopharmacology.org/GRAC/ObjectDisplayForward?objectId=2455, http://www.guidetopharmacology.org/GRAC/ObjectDisplayForward?objectId=2444	http://www.guidetopharmacology.org/GRAC/ObjectDisplayForward?objectId=2455, http://www.guidetopharmacology.org/GRAC/ObjectDisplayForward?objectId=2445	http://www.guidetopharmacology.org/GRAC/ObjectDisplayForward?objectId=2455, http://www.guidetopharmacology.org/GRAC/ObjectDisplayForward?objectId=2438
Ligands	–	http://www.guidetopharmacology.org/GRAC/LigandDisplayForward?ligandId=6754 (https://www.genenames.org/data/gene‐symbol‐report/#!/hgnc_id/HGNC:3778, http://www.uniprot.org/uniprot/P02751)	laminin	collagen
Antibodies	http://www.guidetopharmacology.org/GRAC/LigandDisplayForward?ligandId=7437 (Antagonist) (pIC_50_ 8.3) [179]	http://www.guidetopharmacology.org/GRAC/LigandDisplayForward?ligandId=10236 (Binding) (p*K*_d_ 9.5) [http://www.ncbi.nlm.nih.gov/pubmed/17786386?dopt=AbstractPlus, http://www.ncbi.nlm.nih.gov/pubmed/18042290?dopt=AbstractPlus]	–	–

**Table:** 

Nomenclature	http://www.guidetopharmacology.org/GRAC/ObjectDisplayForward?objectId=2869	http://www.guidetopharmacology.org/GRAC/ObjectDisplayForward?objectId=2799	http://www.guidetopharmacology.org/GRAC/ObjectDisplayForward?objectId=2582	http://www.guidetopharmacology.org/GRAC/ObjectDisplayForward?objectId=2583
Subunits	http://www.guidetopharmacology.org/GRAC/ObjectDisplayForward?objectId=2455, http://www.guidetopharmacology.org/GRAC/ObjectDisplayForward?objectId=2439	http://www.guidetopharmacology.org/GRAC/ObjectDisplayForward?objectId=2450, http://www.guidetopharmacology.org/GRAC/ObjectDisplayForward?objectId=2461	http://www.guidetopharmacology.org/GRAC/ObjectDisplayForward?objectId=2456, http://www.guidetopharmacology.org/GRAC/ObjectDisplayForward?objectId=2451	http://www.guidetopharmacology.org/GRAC/ObjectDisplayForward?objectId=2457, http://www.guidetopharmacology.org/GRAC/ObjectDisplayForward?objectId=2453
Ligands	collagen	E‐cadherin	http://www.guidetopharmacology.org/GRAC/LigandDisplayForward?ligandId=6757 (https://www.genenames.org/data/gene‐symbol‐report/#!/hgnc_id/HGNC:5344, http://www.uniprot.org/uniprot/P05362), http://www.guidetopharmacology.org/GRAC/LigandDisplayForward?ligandId=6756 (https://www.genenames.org/data/gene‐symbol‐report/#!/hgnc_id/HGNC:5345, http://www.uniprot.org/uniprot/P13598)	http://www.guidetopharmacology.org/GRAC/LigandDisplayForward?ligandId=6746 (https://www.genenames.org/data/gene‐symbol‐report/#!/hgnc_id/HGNC:12724, http://www.uniprot.org/uniprot/P04004), http://www.guidetopharmacology.org/GRAC/LigandDisplayForward?ligandId=6754 (https://www.genenames.org/data/gene‐symbol‐report/#!/hgnc_id/HGNC:3778, http://www.uniprot.org/uniprot/P02751), http://www.guidetopharmacology.org/GRAC/LigandDisplayForward?ligandId=6749 (https://www.genenames.org/data/gene‐symbol‐report/#!/hgnc_id/HGNC:3661 https://www.genenames.org/data/gene‐symbol‐report/#!/hgnc_id/HGNC:3662 https://www.genenames.org/data/gene‐symbol‐report/#!/hgnc_id/HGNC:3694, http://www.uniprot.org/uniprot/P02671 http://www.uniprot.org/uniprot/P02675 http://www.uniprot.org/uniprot/P02679), http://www.guidetopharmacology.org/GRAC/LigandDisplayForward?ligandId=6753 (https://www.genenames.org/data/gene‐symbol‐report/#!/hgnc_id/HGNC:11255, http://www.uniprot.org/uniprot/P10451), http://www.guidetopharmacology.org/GRAC/LigandDisplayForward?ligandId=6755 (https://www.genenames.org/data/gene‐symbol‐report/#!/hgnc_id/HGNC:12726, http://www.uniprot.org/uniprot/P04275), thrombospondin, tenascin
Activators	–	–	–	http://www.guidetopharmacology.org/GRAC/LigandDisplayForward?ligandId=9284 (p*K*_d_ 7.9) [http://www.ncbi.nlm.nih.gov/pubmed/20508901?dopt=AbstractPlus]
Inhibitors	–	–	http://www.guidetopharmacology.org/GRAC/LigandDisplayForward?ligandId=6592 (pIC_50_ 7.4–7.5) [http://www.ncbi.nlm.nih.gov/pubmed/11052808?dopt=AbstractPlus]	http://www.guidetopharmacology.org/GRAC/LigandDisplayForward?ligandId=6594 (pIC_50_ 11.7) [http://www.ncbi.nlm.nih.gov/pubmed/15634795?dopt=AbstractPlus], http://www.guidetopharmacology.org/GRAC/LigandDisplayForward?ligandId=6595 (pIC_50_ 11.6) [http://www.ncbi.nlm.nih.gov/pubmed/15634795?dopt=AbstractPlus], http://www.guidetopharmacology.org/GRAC/LigandDisplayForward?ligandId=6597 (pIC_50_ 8.5) [http://www.ncbi.nlm.nih.gov/pubmed/11855984?dopt=AbstractPlus]
Antibodies	–	–	–	http://www.guidetopharmacology.org/GRAC/LigandDisplayForward?ligandId=6596 (Binding) (p*K*_d_ 6.3) [237]

**Table:** 

Nomenclature	http://www.guidetopharmacology.org/GRAC/ObjectDisplayForward?objectId=2437	http://www.guidetopharmacology.org/GRAC/ObjectDisplayForward?objectId=2440	http://www.guidetopharmacology.org/GRAC/ObjectDisplayForward?objectId=2441	http://www.guidetopharmacology.org/GRAC/ObjectDisplayForward?objectId=2442	http://www.guidetopharmacology.org/GRAC/ObjectDisplayForward?objectId=2443	http://www.guidetopharmacology.org/GRAC/ObjectDisplayForward?objectId=2444
HGNC, UniProt	https://www.genenames.org/data/gene‐symbol‐report/#!/hgnc_id/HGNC:6134, http://www.uniprot.org/uniprot/P56199	https://www.genenames.org/data/gene‐symbol‐report/#!/hgnc_id/HGNC:6137, http://www.uniprot.org/uniprot/P08514	https://www.genenames.org/data/gene‐symbol‐report/#!/hgnc_id/HGNC:6138, http://www.uniprot.org/uniprot/P17301	https://www.genenames.org/data/gene‐symbol‐report/#!/hgnc_id/HGNC:6139, http://www.uniprot.org/uniprot/P26006	https://www.genenames.org/data/gene‐symbol‐report/#!/hgnc_id/HGNC:6140, http://www.uniprot.org/uniprot/P13612	https://www.genenames.org/data/gene‐symbol‐report/#!/hgnc_id/HGNC:6141, http://www.uniprot.org/uniprot/P08648
Ligands	–	–	–	http://www.guidetopharmacology.org/GRAC/LigandDisplayForward?ligandId=8849 (Binding) (pIC_50_ 7.2) [http://www.ncbi.nlm.nih.gov/pubmed/19055415?dopt=AbstractPlus]	–	–
Antibodies	–	–	–	–	http://www.guidetopharmacology.org/GRAC/LigandDisplayForward?ligandId=6591 (Inhibition) [http://www.ncbi.nlm.nih.gov/pubmed/15293871?dopt=AbstractPlus]	http://www.guidetopharmacology.org/GRAC/LigandDisplayForward?ligandId=10236 (Binding) (p*K*_d_ 9.5) [http://www.ncbi.nlm.nih.gov/pubmed/17786386?dopt=AbstractPlus, http://www.ncbi.nlm.nih.gov/pubmed/18042290?dopt=AbstractPlus]

**Table:** 

Nomenclature	http://www.guidetopharmacology.org/GRAC/ObjectDisplayForward?objectId=2445	http://www.guidetopharmacology.org/GRAC/ObjectDisplayForward?objectId=2446	http://www.guidetopharmacology.org/GRAC/ObjectDisplayForward?objectId=2447	http://www.guidetopharmacology.org/GRAC/ObjectDisplayForward?objectId=2448	http://www.guidetopharmacology.org/GRAC/ObjectDisplayForward?objectId=2438	http://www.guidetopharmacology.org/GRAC/ObjectDisplayForward?objectId=2439	http://www.guidetopharmacology.org/GRAC/ObjectDisplayForward?objectId=2449
HGNC, UniProt	https://www.genenames.org/data/gene‐symbol‐report/#!/hgnc_id/HGNC:6142, http://www.uniprot.org/uniprot/P23229	https://www.genenames.org/data/gene‐symbol‐report/#!/hgnc_id/HGNC:6143, http://www.uniprot.org/uniprot/Q13683	https://www.genenames.org/data/gene‐symbol‐report/#!/hgnc_id/HGNC:6144, http://www.uniprot.org/uniprot/P53708	https://www.genenames.org/data/gene‐symbol‐report/#!/hgnc_id/HGNC:6145, http://www.uniprot.org/uniprot/Q13797	https://www.genenames.org/data/gene‐symbol‐report/#!/hgnc_id/HGNC:6135, http://www.uniprot.org/uniprot/O75578	https://www.genenames.org/data/gene‐symbol‐report/#!/hgnc_id/HGNC:6136, http://www.uniprot.org/uniprot/Q9UKX5	https://www.genenames.org/data/gene‐symbol‐report/#!/hgnc_id/HGNC:6146, http://www.uniprot.org/uniprot/Q13349

**Table:** 

Nomenclature	https://www.guidetopharmacology.org/GRAC/ObjectDisplayForward?objectId=2450	https://www.guidetopharmacology.org/GRAC/ObjectDisplayForward?objectId=2451	https://www.guidetopharmacology.org/GRAC/ObjectDisplayForward?objectId=2452	https://www.guidetopharmacology.org/GRAC/ObjectDisplayForward?objectId=2453	https://www.guidetopharmacology.org/GRAC/ObjectDisplayForward?objectId=2454	https://www.guidetopharmacology.org/GRAC/ObjectDisplayForward?objectId=2455
HGNC, UniProt	https://www.genenames.org/data/gene‐symbol‐report/#!/hgnc_id/HGNC:6147, http://www.uniprot.org/uniprot/P38570	https://www.genenames.org/data/gene‐symbol‐report/#!/hgnc_id/HGNC:6148, http://www.uniprot.org/uniprot/P20701	https://www.genenames.org/data/gene‐symbol‐report/#!/hgnc_id/HGNC:6149, http://www.uniprot.org/uniprot/P11215	https://www.genenames.org/data/gene‐symbol‐report/#!/hgnc_id/HGNC:6150, http://www.uniprot.org/uniprot/P06756	https://www.genenames.org/data/gene‐symbol‐report/#!/hgnc_id/HGNC:6152, http://www.uniprot.org/uniprot/P20702	https://www.genenames.org/data/gene‐symbol‐report/#!/hgnc_id/HGNC:6153, http://www.uniprot.org/uniprot/P05556
Antagonists	–	http://www.guidetopharmacology.org/GRAC/LigandDisplayForward?ligandId=7533 (Inhibition) [18, http://www.ncbi.nlm.nih.gov/pubmed/24900456?dopt=AbstractPlus]	–	http://www.guidetopharmacology.org/GRAC/LigandDisplayForward?ligandId=9689 (pIC_50_ 7.1) [http://www.ncbi.nlm.nih.gov/pubmed/14561098?dopt=AbstractPlus]	–	–
Antibodies	–	http://www.guidetopharmacology.org/GRAC/LigandDisplayForward?ligandId=6593 (Binding) (p*K*_d_ 11.4) [100]	–	–	–	–

**Table:** 

Nomenclature	http://www.guidetopharmacology.org/GRAC/ObjectDisplayForward?objectId=2456	http://www.guidetopharmacology.org/GRAC/ObjectDisplayForward?objectId=2457	http://www.guidetopharmacology.org/GRAC/ObjectDisplayForward?objectId=2458	http://www.guidetopharmacology.org/GRAC/ObjectDisplayForward?objectId=2459	http://www.guidetopharmacology.org/GRAC/ObjectDisplayForward?objectId=2460	http://www.guidetopharmacology.org/GRAC/ObjectDisplayForward?objectId=2461	http://www.guidetopharmacology.org/GRAC/ObjectDisplayForward?objectId=2462
HGNC, UniProt	https://www.genenames.org/data/gene‐symbol‐report/#!/hgnc_id/HGNC:6155, http://www.uniprot.org/uniprot/P05107	https://www.genenames.org/data/gene‐symbol‐report/#!/hgnc_id/HGNC:6156, http://www.uniprot.org/uniprot/P05106	https://www.genenames.org/data/gene‐symbol‐report/#!/hgnc_id/HGNC:6158, http://www.uniprot.org/uniprot/P16144	https://www.genenames.org/data/gene‐symbol‐report/#!/hgnc_id/HGNC:6160, http://www.uniprot.org/uniprot/P18084	https://www.genenames.org/data/gene‐symbol‐report/#!/hgnc_id/HGNC:6161, http://www.uniprot.org/uniprot/P18564	https://www.genenames.org/data/gene‐symbol‐report/#!/hgnc_id/HGNC:6162, http://www.uniprot.org/uniprot/P26010	https://www.genenames.org/data/gene‐symbol‐report/#!/hgnc_id/HGNC:6163, http://www.uniprot.org/uniprot/P26012

### Comments: Integrin ligands

**Collagen** is the most abundant protein in metazoa, rich in glycine and proline residues, made up of cross‐linked triple helical structures, generated primarily by fibroblasts. Extensive post‐translational processing is conducted by prolyl and lysyl hydroxylases, as well as transglutaminases. Over 40 genes for collagen‐α subunits have been identified in the human genome. The collagen‐binding integrins α1β1, α2β1, α10β1 and α11β1 recognise a range of triple‐helical peptide motifs including GFOGER (O = hydroxyproline), a synthetic peptide derived from the primary sequence of collagen I (http://www.guidetopharmacology.org/GRAC/LigandDisplayForward?ligandId=4898 (http://www.guidetopharmacology.org/GRAC/LigandDisplayForward?ligandId=2197, http://www.uniprot.org/uniprot/P02452)) and collagen II (http://www.guidetopharmacology.org/GRAC/LigandDisplayForward?ligandId=4899 (http://www.guidetopharmacology.org/GRAC/LigandDisplayForward?ligandId=2200, http://www.uniprot.org/uniprot/P02458)).

**Laminin** is an extracellular glycoprotein composed of α, β and γ chains, for which five, four and three genes, respectively, are identified in the human genome. It binds to α1β1, α2β1, α3,β1, α7β1 and α6β4 integrins10.

http://www.guidetopharmacology.org/GRAC/LigandDisplayForward?ligandId=6749 (https://www.genenames.org/data/gene‐symbol‐report/#!/hgnc_id/HGNC:3661 https://www.genenames.org/data/gene‐symbol‐report/#!/hgnc_id/HGNC:3662 https://www.genenames.org/data/gene‐symbol‐report/#!/hgnc_id/HGNC:3694, http://www.uniprot.org/uniprot/P02671 http://www.uniprot.org/uniprot/P02675 http://www.uniprot.org/uniprot/P02679) is a glycosylated hexamer composed of two α (https://www.genenames.org/data/gene‐symbol‐report/#!/hgnc_id/HGNC:3661, http://www.uniprot.org/uniprot/P02671), two β (https://www.genenames.org/data/gene‐symbol‐report/#!/hgnc_id/HGNC:3662, http://www.uniprot.org/uniprot/P02675) and two γ (https://www.genenames.org/data/gene‐symbol‐report/#!/hgnc_id/HGNC:3694, http://www.uniprot.org/uniprot/P02679,) subunits, linked by disulphide bridges. It is found in plasma and alpha granules of platelets. It forms cross‐links between activated platelets mediating aggregation by binding αIIbβ3; proteolysis by thrombin cleaves short peptides termed fibrinopeptides to generate fibrin, which polymerises as part of the blood coagulation cascade.

http://www.guidetopharmacology.org/GRAC/LigandDisplayForward?ligandId=6754 (https://www.genenames.org/data/gene‐symbol‐report/#!/hgnc_id/HGNC:3778, http://www.uniprot.org/uniprot/P02751) is a disulphide‐linked homodimer found as two major forms; a soluble dimeric form found in the plasma and a tissue version that is polymeric, which is secreted into the extracellular matrix by fibroblasts. Splice variation of the gene product (https://www.genenames.org/data/gene‐symbol‐report/#!/hgnc_id/HGNC:3778, http://www.uniprot.org/uniprot/P02751) generates multiple isoforms.

http://www.guidetopharmacology.org/GRAC/LigandDisplayForward?ligandId=6746 (https://www.genenames.org/data/gene‐symbol‐report/#!/hgnc_id/HGNC:12724, http://www.uniprot.org/uniprot/P04004) is a serum glycoprotein and extracellular matrix protein which is found either as a monomer or, following proteolysis, a disulphide ‐linked dimer.

http://www.guidetopharmacology.org/GRAC/LigandDisplayForward?ligandId=6753 (https://www.genenames.org/data/gene‐symbol‐report/#!/hgnc_id/HGNC:11255, http://www.uniprot.org/uniprot/P10451) forms an integral part of the mineralized matrix in bone, where it undergoes extensive posttranslation processing, including proteolysis and phosphorylation.

http://www.guidetopharmacology.org/GRAC/LigandDisplayForward?ligandId=6755 (https://www.genenames.org/data/gene‐symbol‐report/#!/hgnc_id/HGNC:12726, http://www.uniprot.org/uniprot/P04275) is a glycoprotein synthesised in vascular endothelial cells as a disulphide‐linked homodimer, but multimerises further in plasma and is deposited on vessel wall collagen as a high molecular weight multimer. It is responsible for capturing platelets under arterial shear flow (*via* GPIb) and in thrombus propagation (via integrin αIIbβ3).

### Further reading on Integrins

Clemetson KJ. (2017) The origins of major platelet receptor nomenclature. *Platelets* **28**: 40‐42 https://www.ncbi.nlm.nih.gov/pubmed/27715379?dopt=AbstractPlus

Emsley J *et al*. (2000) Structural basis of collagen recognition by integrin alpha2beta1. *Cell* **101**: 47‐56 https://www.ncbi.nlm.nih.gov/pubmed/10778855?dopt=AbstractPlus

Hamidi H *et al*. (2016) The complexity of integrins in cancer and new scopes for therapeutic targeting. *Br. J. Cancer* **115**: 1017‐1023 https://www.ncbi.nlm.nih.gov/pubmed/27685444?dopt=AbstractPlus

Horton ER *et al*. (2016) The integrin adhesome network at a glance. *J. Cell. Sci*. **129**: 4159‐4163 https://www.ncbi.nlm.nih.gov/pubmed/27799358?dopt=AbstractPlus

Ley K *et al*. (2016) Integrin‐based therapeutics: biological basis, clinical use and new drugs. *Nat Rev Drug Discov* **15**: 173‐83 https://www.ncbi.nlm.nih.gov/pubmed/26822833?dopt=AbstractPlus

Manninen A *et al*. (2017) A proteomics view on integrin‐mediated adhesions. *Proteomics* **17**: https://www.ncbi.nlm.nih.gov/pubmed/27723259?dopt=AbstractPlus

Raab‐Westphal S *et al*. (2017) Integrins as Therapeutic Targets: Successes and Cancers. *Cancers (Basel)* **9**: https://www.ncbi.nlm.nih.gov/pubmed/28832494?dopt=AbstractPlus

## http://www.guidetopharmacology.org/GRAC/FamilyDisplayForward?familyId=302

### Overview

Pattern Recognition Receptors (PRRs, [http://www.ncbi.nlm.nih.gov/pubmed/20303872?dopt=AbstractPlus]) (**nomenclature as agreed by NC‐IUPHAR sub‐committee on Pattern Recognition Receptors**, [http://www.ncbi.nlm.nih.gov/pubmed/25829385?dopt=AbstractPlus]) participate in the innate immune response to microbial agents, the stimulation of which leads to activation of intracellular enzymes and regulation of gene transcription. PRRs express multiple leucine‐rich regions to bind a range of microbially‐derived ligands, termed PAMPs or pathogen‐associated molecular patterns or endogenous ligands, termed DAMPS or damage‐associated molecular patterns. These include peptides, carbohydrates, peptidoglycans, lipoproteins, lipopolysaccharides, and nucleic acids. PRRs include both cell‐surface and intracellular proteins. PRRs may be divided into signalling‐associated members, identified here, and endocytic members, the function of which appears to be to recognise particular microbial motifs for subsequent cell attachment, internalisation and destruction. Some are involved in inflammasome formation, and modulation of IL‐1β cleavage and secretion, and others in the initiation of the type I interferon response.

PRRs included in the Guide To PHARMACOLOGY are:

**Catalytic PRRs** (see links below this overview)

Toll‐like receptors (TLRs)

Nucleotide‐binding oligomerization domain, leucine‐rich repeat containing receptors (NLRs, also known as NOD (Nucleotide oligomerisation domain)‐like receptors)

RIG‐I‐like receptors (RLRs)

http://www.guidetopharmacology.org/GRAC/ObjectDisplayForward?objectId=1620 and http://www.guidetopharmacology.org/GRAC/ObjectDisplayForward?objectId=1621

**Non‐catalytic PRRs**

http://www.guidetopharmacology.org/GRAC/FamilyDisplayForward?familyId=942 (ALRs)

http://www.guidetopharmacology.org/GRAC/FamilyDisplayForward?familyId=945

http://www.guidetopharmacology.org/GRAC/FamilyDisplayForward?familyId=929

http://www.guidetopharmacology.org/GRAC/ObjectDisplayForward?objectId=2843 (RAGE)

## http://www.guidetopharmacology.org/GRAC/FamilyDisplayForward?familyId=316

### Overview

Members of the toll‐like family of receptors (nomenclature recommended by the NC‐IUPHAR subcommittee on pattern recognition receptors, [http://www.ncbi.nlm.nih.gov/pubmed/25829385?dopt=AbstractPlus]) share significant homology with the interleukin‐1 receptor family and appear to require dimerization either as homo‐ or heterodimers for functional activity. Heterodimerization appears to influence the potency of ligand binding substantially (*e.g*. TLR1/2 and TLR2/6, [http://www.ncbi.nlm.nih.gov/pubmed/11431423?dopt=AbstractPlus, http://www.ncbi.nlm.nih.gov/pubmed/12077222?dopt=AbstractPlus]). TLR1, TLR2, TLR4, TLR5, TLR6 and TLR11 are cell‐surface proteins, while other members are associated with intracellular organelles, signalling through the MyD88‐dependent pathways (with the exception of TLR3). As well as responding to exogenous infectious agents, it has been suggested that selected members of the family may be activated by endogenous ligands, such as http://www.guidetopharmacology.org/GRAC/LigandDisplayForward?ligandId=4953 (https://www.genenames.org/data/gene‐symbol‐report/#!/hgnc_id/HGNC:5261, http://www.uniprot.org/uniprot/P10809) [http://www.ncbi.nlm.nih.gov/pubmed/10623794?dopt=AbstractPlus].

**Table:** 

Nomenclature	http://www.guidetopharmacology.org/GRAC/ObjectDisplayForward?objectId=1751	http://www.guidetopharmacology.org/GRAC/ObjectDisplayForward?objectId=1752	http://www.guidetopharmacology.org/GRAC/ObjectDisplayForward?objectId=1753	http://www.guidetopharmacology.org/GRAC/ObjectDisplayForward?objectId=1754	http://www.guidetopharmacology.org/GRAC/ObjectDisplayForward?objectId=1755
HGNC, UniProt	https://www.genenames.org/data/gene‐symbol‐report/#!/hgnc_id/HGNC:11847, http://www.uniprot.org/uniprot/Q15399	https://www.genenames.org/data/gene‐symbol‐report/#!/hgnc_id/HGNC:11848, http://www.uniprot.org/uniprot/O60603	https://www.genenames.org/data/gene‐symbol‐report/#!/hgnc_id/HGNC:11849, http://www.uniprot.org/uniprot/O15455	https://www.genenames.org/data/gene‐symbol‐report/#!/hgnc_id/HGNC:11850, http://www.uniprot.org/uniprot/O00206	https://www.genenames.org/data/gene‐symbol‐report/#!/hgnc_id/HGNC:11851, http://www.uniprot.org/uniprot/O60602
Agonists	–	http://www.guidetopharmacology.org/GRAC/LigandDisplayForward?ligandId=8888 [http://www.ncbi.nlm.nih.gov/pubmed/23098072?dopt=AbstractPlus], http://www.guidetopharmacology.org/GRAC/LigandDisplayForward?ligandId=5044 [http://www.ncbi.nlm.nih.gov/pubmed/10364168?dopt=AbstractPlus, http://www.ncbi.nlm.nih.gov/pubmed/10384090?dopt=AbstractPlus]	http://www.guidetopharmacology.org/GRAC/LigandDisplayForward?ligandId=5047 [http://www.ncbi.nlm.nih.gov/pubmed/11607032?dopt=AbstractPlus]	http://www.guidetopharmacology.org/GRAC/LigandDisplayForward?ligandId=5019 [http://www.ncbi.nlm.nih.gov/pubmed/9851930?dopt=AbstractPlus], http://www.guidetopharmacology.org/GRAC/LigandDisplayForward?ligandId=2770 [http://www.ncbi.nlm.nih.gov/pubmed/10644670?dopt=AbstractPlus] – Mouse	http://www.guidetopharmacology.org/GRAC/LigandDisplayForward?ligandId=4931 [http://www.ncbi.nlm.nih.gov/pubmed/11323673?dopt=AbstractPlus]
Selective antagonists	–	–	–	http://www.guidetopharmacology.org/GRAC/LigandDisplayForward?ligandId=9036 [http://www.ncbi.nlm.nih.gov/pubmed/16373689?dopt=AbstractPlus]	–
Comments	Functions as a heterodimer with TLR2 in detection of triacylated lipoproteins. Activated by the synthetic analogue http://www.guidetopharmacology.org/GRAC/LigandDisplayForward?ligandId=8558.	Functions as a heterodimer with either TLR1 or TLR6 in the detection of triacylated and diacylated lipopeptides respectively. TLR1/2 and 2/6 heterodimers can be activated by the synthetic lipopeptides http://www.guidetopharmacology.org/GRAC/LigandDisplayForward?ligandId=8558 and http://www.guidetopharmacology.org/GRAC/LigandDisplayForward?ligandId=8560 respectively. There is some debate in the field as to whether or not peptidoglycan is a direct agonist of TLR2, or whether the early studies reporting this contained contaminating lipoproteins.	Involved in endosomal detection of dsRNA; pro‐inflammatory.	http://www.guidetopharmacology.org/GRAC/LigandDisplayForward?ligandId=4919 (E5564) is a lipid A analogue, which has been described as a TLR4 antagonist [http://www.ncbi.nlm.nih.gov/pubmed/9820516?dopt=AbstractPlus]. TLR4 signals in conjunction with the co‐factor http://www.guidetopharmacology.org/GRAC/ObjectDisplayForward?objectId=2890.	Involved in the detection of bacterial flagellin; pro‐inflammatory.

**Table:** 

Nomenclature	http://www.guidetopharmacology.org/GRAC/ObjectDisplayForward?objectId=1756	http://www.guidetopharmacology.org/GRAC/ObjectDisplayForward?objectId=1757	http://www.guidetopharmacology.org/GRAC/ObjectDisplayForward?objectId=1758	http://www.guidetopharmacology.org/GRAC/ObjectDisplayForward?objectId=1759	http://www.guidetopharmacology.org/GRAC/ObjectDisplayForward?objectId=1760	http://www.guidetopharmacology.org/GRAC/ObjectDisplayForward?objectId=1761
HGNC, UniProt	https://www.genenames.org/data/gene‐symbol‐report/#!/hgnc_id/HGNC:16711, http://www.uniprot.org/uniprot/Q9Y2C9	https://www.genenames.org/data/gene‐symbol‐report/#!/hgnc_id/HGNC:15631, http://www.uniprot.org/uniprot/Q9NYK1	https://www.genenames.org/data/gene‐symbol‐report/#!/hgnc_id/HGNC:15632, http://www.uniprot.org/uniprot/Q9NR97	https://www.genenames.org/data/gene‐symbol‐report/#!/hgnc_id/HGNC:15633, http://www.uniprot.org/uniprot/Q9NR96	https://www.genenames.org/data/gene‐symbol‐report/#!/hgnc_id/HGNC:15634, http://www.uniprot.org/uniprot/Q9BXR5	–
Agonists	–	http://www.guidetopharmacology.org/GRAC/LigandDisplayForward?ligandId=5051 [http://www.ncbi.nlm.nih.gov/pubmed/11812998?dopt=AbstractPlus, http://www.ncbi.nlm.nih.gov/pubmed/12032557?dopt=AbstractPlus, 121], http://www.guidetopharmacology.org/GRAC/LigandDisplayForward?ligandId=5003 [121], http://www.guidetopharmacology.org/GRAC/LigandDisplayForward?ligandId=5018 [http://www.ncbi.nlm.nih.gov/pubmed/14579267?dopt=AbstractPlus]	http://www.guidetopharmacology.org/GRAC/LigandDisplayForward?ligandId=5051 [http://www.ncbi.nlm.nih.gov/pubmed/11812998?dopt=AbstractPlus, http://www.ncbi.nlm.nih.gov/pubmed/12032557?dopt=AbstractPlus, 121]	–	–	–
Antagonists	–	http://www.guidetopharmacology.org/GRAC/LigandDisplayForward?ligandId=7198 (pIC_50_ 5.6) [http://www.ncbi.nlm.nih.gov/pubmed/24342772?dopt=AbstractPlus]	–	http://www.guidetopharmacology.org/GRAC/LigandDisplayForward?ligandId=7198 (pIC_50_ 7.1) [http://www.ncbi.nlm.nih.gov/pubmed/24342772?dopt=AbstractPlus]	–	–
Comments	Functions as a heterodimer with TLR2. Involved in the pro‐inflammatory response to diacylated bacterial lipopeptides.	Activated by imidazoquinoline derivatives and RNA oligoribonucleotides. Involved in endosomal detection of ssRNA; pro‐inflammatory.	Activated by imidazoquinoline derivatives and RNA oligoribonucleotides. Endosomal detection of ssRNA; pro‐inflammatory.	Toll‐like receptor 9 interacts with unmethylated CpG dinucleotides from bacterial DNA [http://www.ncbi.nlm.nih.gov/pubmed/11130078?dopt=AbstractPlus]. Activated by CpG rich DNA sequences; pro‐inflammatory.	TLR10 is the only pattern‐recognition receptor without known ligand specificity and biological function. Evidence suggests it plays a modulatory role with predominantly inhibitory (anti‐inflammatory) actions [http://www.ncbi.nlm.nih.gov/pubmed/25288745?dopt=AbstractPlus]. Murine TLR10 has a retroviral insertion that makes it non‐functional.	Found in mouse

### Further reading on Toll‐like receptor family

Anthoney N *et al*. (2018) Toll and Toll‐like receptor signalling in development. *Development* **145**: https://www.ncbi.nlm.nih.gov/pubmed/29695493?dopt=AbstractPlus

Bryant CE *et al*. (2015) International Union of Basic and Clinical Pharmacology. XCVI. Pattern recognition receptors in health and disease. *Pharmacol. Rev*. **67**: 462‐504 https://www.ncbi.nlm.nih.gov/pubmed/25829385?dopt=AbstractPlus

Franz KM *et al*. (2017) Innate Immune Receptors as Competitive Determinants of Cell Fate. *Mol. Cell* **66**: 750‐760 https://www.ncbi.nlm.nih.gov/pubmed/28622520?dopt=AbstractPlus

Joosten LA *et al*. (2016) Toll‐like receptors and chronic inflammation in rheumatic diseases: new developments. *Nat Rev Rheumatol* **12**: 344‐57 https://www.ncbi.nlm.nih.gov/pubmed/27170508?dopt=AbstractPlus

Nunes KP *et al*. (2018) Targeting toll‐like receptor 4 signalling pathways: can therapeutics pay the toll for hypertension? *Br. J. Pharmacol*. https://www.ncbi.nlm.nih.gov/pubmed/29981161?dopt=AbstractPlus

Zhang Z *et al*. (2017) Toward a structural understanding of nucleic acid‐sensing Toll‐like receptors in the innate immune system. *FEBS Lett*. **591**: 3167‐3181 https://www.ncbi.nlm.nih.gov/pubmed/28686285?dopt=AbstractPlus

## http://www.guidetopharmacology.org/GRAC/FamilyDisplayForward?familyId=317

### Overview

The nucleotide‐binding oligomerization domain, leucine‐rich repeat (NLR) family of receptors (nomenclature recommended by the NC‐IUPHAR subcommittee on pattern recognition receptors [http://www.ncbi.nlm.nih.gov/pubmed/25829385?dopt=AbstractPlus]) share a common domain organisation. This consists of an N‐terminal effector domain, a central nucleotidebinding and oligomerization domain (NOD; also referred to as a NACHT domain), and C‐terminal leucine‐rich repeats (LRR) which have regulatory and ligand recognition functions. The type of effector domain has resulted in the division of NLR family members into two major sub‐families, NLRC and NLRP, along with three smaller sub‐families NLRA, NLRB and NLRX [http://www.ncbi.nlm.nih.gov/pubmed/18341998?dopt=AbstractPlus]. NLRC members express an N‐terminal caspase recruitment domain (CARD) and NLRP members an N‐terminal Pyrin domain (PYD).

Upon activation the NLRC family members NOD1 (NLRC1) and NOD2 (NLRC2) recruit a serine/threonine kinase http://www.guidetopharmacology.org/GRAC/ObjectDisplayForward?objectId=2190 (receptor interacting serine/threonine kinase 2, http://www.uniprot.org/uniprot/O43353, also known as CARD3, CARDIAK, RICK, RIP2) leading to signalling through NF*κ*B and MAP kinase. Activation of NLRC4 (previously known as IPAF) and members of the NLRP3 family, including NLRP1 and NLRP3, leads to formation of a large multiprotein complex known as the inflammasome. In addition to NLR proteins other key members of the inflammasome include the adaptor protein ASC (apoptosis‐associated speck‐like protein containing a CARD, also known as *PYCARD*, CARD5, TMS1, http://www.uniprot.org/uniprot/Q9ULZ3) and inflammatory caspases. The inflammasome activates the pro‐inflammatory cytokines http://www.guidetopharmacology.org/GRAC/LigandDisplayForward?ligandId=4974 (https://www.genenames.org/data/gene‐symbol‐report/#!/hgnc_id/HGNC:5992, http://www.uniprot.org/uniprot/P01584) and http://www.guidetopharmacology.org/GRAC/LigandDisplayForward?ligandId=4983 (https://www.genenames.org/data/gene‐symbol‐report/#!/hgnc_id/HGNC:5986, http://www.uniprot.org/uniprot/Q14116) [http://www.ncbi.nlm.nih.gov/pubmed/25829385?dopt=AbstractPlus, http://www.ncbi.nlm.nih.gov/pubmed/21219188?dopt=AbstractPlus]

**Table:** 

Nomenclature	http://www.guidetopharmacology.org/GRAC/ObjectDisplayForward?objectId=1762	http://www.guidetopharmacology.org/GRAC/ObjectDisplayForward?objectId=1763	http://www.guidetopharmacology.org/GRAC/ObjectDisplayForward?objectId=1764	http://www.guidetopharmacology.org/GRAC/ObjectDisplayForward?objectId=1782	http://www.guidetopharmacology.org/GRAC/ObjectDisplayForward?objectId=1765	http://www.guidetopharmacology.org/GRAC/ObjectDisplayForward?objectId=1766	http://www.guidetopharmacology.org/GRAC/ObjectDisplayForward?objectId=1767
Common abbreviation	NOD1	NOD2	–	–	–	–	–
HGNC, UniProt	https://www.genenames.org/data/gene‐symbol‐report/#!/hgnc_id/HGNC:16390, http://www.uniprot.org/uniprot/Q9Y239	https://www.genenames.org/data/gene‐symbol‐report/#!/hgnc_id/HGNC:5331, http://www.uniprot.org/uniprot/Q9HC29	https://www.genenames.org/data/gene‐symbol‐report/#!/hgnc_id/HGNC:29889, http://www.uniprot.org/uniprot/Q7RTR2	https://www.genenames.org/data/gene‐symbol‐report/#!/hgnc_id/HGNC:16412, http://www.uniprot.org/uniprot/Q9NPP4	https://www.genenames.org/data/gene‐symbol‐report/#!/hgnc_id/HGNC:29933, http://www.uniprot.org/uniprot/Q86WI3	https://www.genenames.org/data/gene‐symbol‐report/#!/hgnc_id/HGNC:29890, http://www.uniprot.org/uniprot/Q86UT6	https://www.genenames.org/data/gene‐symbol‐report/#!/hgnc_id/HGNC:7067, http://www.uniprot.org/uniprot/P33076
Agonists	http://www.guidetopharmacology.org/GRAC/LigandDisplayForward?ligandId=5021	http://www.guidetopharmacology.org/GRAC/LigandDisplayForward?ligandId=5024	–	–	–	–	–
Comments	–	NOD2 has also been reported to be activated by ssRNA [http://www.ncbi.nlm.nih.gov/pubmed/19701189?dopt=AbstractPlus] although this has not been widely reproduced.	–	NLRC4 forms an inflammasome with the NAIP proteins following recognition of bacterial flagellin and type III secretion system rod proteins by the NAIPs.	–	–	–

**Table:** 

Nomenclature	http://www.guidetopharmacology.org/GRAC/ObjectDisplayForward?objectId=1768	http://www.guidetopharmacology.org/GRAC/ObjectDisplayForward?objectId=1769	http://www.guidetopharmacology.org/GRAC/ObjectDisplayForward?objectId=1770
HGNC, UniProt	https://www.genenames.org/data/gene‐symbol‐report/#!/hgnc_id/HGNC:14374, http://www.uniprot.org/uniprot/Q9C000	https://www.genenames.org/data/gene‐symbol‐report/#!/hgnc_id/HGNC:22948, http://www.uniprot.org/uniprot/Q9NX02	https://www.genenames.org/data/gene‐symbol‐report/#!/hgnc_id/HGNC:16400, http://www.uniprot.org/uniprot/Q96P20
Inhibitors	–	–	http://www.guidetopharmacology.org/GRAC/LigandDisplayForward?ligandId=8228 (pIC_50_ > 8) [http://www.ncbi.nlm.nih.gov/pubmed/25686105?dopt=AbstractPlus]
Agonists	http://www.guidetopharmacology.org/GRAC/LigandDisplayForward?ligandId=5024	–	–
Comments	NLRP1 has 3 murine orthologues which lack the N‐terminal Pyrin domain. Murine NLRP1b (http://www.ensembl.org/Mus_musculus/Gene/Summary?db=core;g=ENSMUSG00000070390;r=11:71153102‐71230733) is the best characterised, responding to Anthrax Lethal Toxin.	Along with NLRP7, NLRP2 is the product of a primate‐specific gene duplication.	NLRP3 has been shown to be activated following disruption of cellular haemostasis by a wide‐variety of exogenous and endogenous molecules. The identity of the precise agonist that interacts with NLRP3 remains enigmatic. Efflux of potassium ions appears to be a common event for NLRP3 activating molecules. In addition to MCC950 [http://www.ncbi.nlm.nih.gov/pubmed/25686105?dopt=AbstractPlus] other small molecules including http://www.guidetopharmacology.org/GRAC/LigandDisplayForward?ligandId=10057 [http://www.ncbi.nlm.nih.gov/pubmed/29021150?dopt=AbstractPlus], β‐hydroxybutyrate [http://www.ncbi.nlm.nih.gov/pubmed/25686106?dopt=AbstractPlus], and various boron containing compounds [http://www.ncbi.nlm.nih.gov/pubmed/28943355?dopt=AbstractPlus] modulate NLRP3.

**Table:** 

Nomenclature	http://www.guidetopharmacology.org/GRAC/ObjectDisplayForward?objectId=1771	http://www.guidetopharmacology.org/GRAC/ObjectDisplayForward?objectId=1772	http://www.guidetopharmacology.org/GRAC/ObjectDisplayForward?objectId=1773	http://www.guidetopharmacology.org/GRAC/ObjectDisplayForward?objectId=1774	http://www.guidetopharmacology.org/GRAC/ObjectDisplayForward?objectId=1775	http://www.guidetopharmacology.org/GRAC/ObjectDisplayForward?objectId=1776
HGNC, UniProt	https://www.genenames.org/data/gene‐symbol‐report/#!/hgnc_id/HGNC:22943, http://www.uniprot.org/uniprot/Q96MN2	https://www.genenames.org/data/gene‐symbol‐report/#!/hgnc_id/HGNC:21269, http://www.uniprot.org/uniprot/P59047	https://www.genenames.org/data/gene‐symbol‐report/#!/hgnc_id/HGNC:22944, http://www.uniprot.org/uniprot/P59044	https://www.genenames.org/data/gene‐symbol‐report/#!/hgnc_id/HGNC:22947, http://www.uniprot.org/uniprot/Q8WX94	https://www.genenames.org/data/gene‐symbol‐report/#!/hgnc_id/HGNC:22940, http://www.uniprot.org/uniprot/Q86W28	https://www.genenames.org/data/gene‐symbol‐report/#!/hgnc_id/HGNC:22941, http://www.uniprot.org/uniprot/Q7RTR0
Comments	Expanded in the mouse resulting in 7 orthologues.	–	–	Absent in mouse. Along with NLRP2 the product of a primate‐specific gene duplication.	Absent in mouse	This receptor has three murine orthologues.

**Table:** 

Nomenclature	http://www.guidetopharmacology.org/GRAC/ObjectDisplayForward?objectId=1777	http://www.guidetopharmacology.org/GRAC/ObjectDisplayForward?objectId=1778	http://www.guidetopharmacology.org/GRAC/ObjectDisplayForward?objectId=1779	http://www.guidetopharmacology.org/GRAC/ObjectDisplayForward?objectId=1780	http://www.guidetopharmacology.org/GRAC/ObjectDisplayForward?objectId=1781
HGNC, UniProt	https://www.genenames.org/data/gene‐symbol‐report/#!/hgnc_id/HGNC:21464, http://www.uniprot.org/uniprot/Q86W26	https://www.genenames.org/data/gene‐symbol‐report/#!/hgnc_id/HGNC:22945, http://www.uniprot.org/uniprot/P59045	https://www.genenames.org/data/gene‐symbol‐report/#!/hgnc_id/HGNC:22938, http://www.uniprot.org/uniprot/P59046	https://www.genenames.org/data/gene‐symbol‐report/#!/hgnc_id/HGNC:22937, http://www.uniprot.org/uniprot/Q86W25	https://www.genenames.org/data/gene‐symbol‐report/#!/hgnc_id/HGNC:22939, http://www.uniprot.org/uniprot/Q86W24
Comments	NLRP10 lacks the LRR region.	Absent in mouse	–	Absent in mouse	–

### Comments

NLRP3 has also been reported to respond to hostderived products, known as danger‐associated molecular patterns, or DAMPs, including http://www.guidetopharmacology.org/GRAC/LigandDisplayForward?ligandId=4731 [http://www.ncbi.nlm.nih.gov/pubmed/16407889?dopt=AbstractPlus], http://www.guidetopharmacology.org/GRAC/LigandDisplayForward?ligandId=1713, http://www.guidetopharmacology.org/GRAC/LigandDisplayForward?ligandId=4719, http://www.guidetopharmacology.org/GRAC/LigandDisplayForward?ligandId=4954 and http://www.guidetopharmacology.org/GRAC/LigandDisplayForward?ligandId=4865 (https://www.genenames.org/data/gene‐symbol‐report/#!/hgnc_id/HGNC:620, http://www.uniprot.org/uniprot/P05067) [http://www.ncbi.nlm.nih.gov/pubmed/20303873?dopt=AbstractPlus].

Loss‐of‐function mutations of NLRP3 are associated with cold autoinflammatory and Muckle‐Wells syndromes.

This family also includes http://www.guidetopharmacology.org/GRAC/ObjectDisplayForward?objectId=2793 (https://www.genenames.org/data/gene‐symbol‐report/#!/hgnc_id/HGNC:7634, http://www.uniprot.org/uniprot/Q13075) which can be found in the ‘Inhibitors of apoptosis (IAP) protein family’ in the http://www.guidetopharmacology.org/GRAC/ReceptorFamiliesForward?type=OTHER section of the Guide.

### Further reading on NOD‐like receptor family

Broz P *et al*. (2016) Inflammasomes: mechanism of assembly, regulation and signalling. *Nat. Rev. Immunol*. **16**: 407‐20 https://www.ncbi.nlm.nih.gov/pubmed/27291964?dopt=AbstractPlus

Bryant CE *et al*. (2015) International Union of Basic and Clinical Pharmacology. XCVI. Pattern recognition receptors in health and disease. *Pharmacol. Rev*. **67**: 462‐504 https://www.ncbi.nlm.nih.gov/pubmed/25829385?dopt=AbstractPlus

Keestra‐Gounder AM *et al*. (2017) NOD1 and NOD2: Beyond Peptidoglycan Sensing. *Trends Immunol*. **38**: 758‐767 https://www.ncbi.nlm.nih.gov/pubmed/28823510?dopt=AbstractPlus

Lei‐Leston AC *et al*. (2017) Epithelial Cell Inflammasomes in Intestinal Immunity and Inflammation. *Front Immunol* **8**: 1168 https://www.ncbi.nlm.nih.gov/pubmed/28979266?dopt=AbstractPlus

Man SM. (2018) Inflammasomes in the gastrointestinal tract: infection, cancer and gut microbiota homeostasis. *Nat Rev Gastroenterol Hepatol* **15**: 721‐737 https://www.ncbi.nlm.nih.gov/pubmed/30185915?dopt=AbstractPlus

Mukherjee T *et al*. (2018) NOD1 and NOD2 in inflammation, immunity and disease. *Arch. Biochem. Biophys*. https://www.ncbi.nlm.nih.gov/pubmed/30578751?dopt=AbstractPlus

Nielsen AE *et al*. (2017) Synthetic agonists of NOD‐like, RIG‐I‐like, and C‐type lectin receptors for probing the inflammatory immune response. *Future Med Chem* **9**: 1345‐1360 https://www.ncbi.nlm.nih.gov/pubmed/28776416?dopt=AbstractPlus

## http://www.guidetopharmacology.org/GRAC/FamilyDisplayForward?familyId=940

### Overview

There are three human RIG‐I‐like receptors (RLRs) which are cytoplasmic pattern recognition receptors (PRRs) of the innate immune system. They detect non‐self cytosolic double‐stranded RNA species and and 5′‐triphosphate single‐stranded RNA from various sources and are essential for inducing production of type I interferons, such as IFNβ, type III interferons, and other anti‐pathogenic effectors [http://www.ncbi.nlm.nih.gov/pubmed/25081315?dopt=AbstractPlus, http://www.ncbi.nlm.nih.gov/pubmed/25829385?dopt=AbstractPlus]. They function as RNA helicases (EC 3.6.4.13) using the energy from ATP hydrolysis to unwind RNA.

**Table:** 

Nomenclature	http://www.guidetopharmacology.org/GRAC/ObjectDisplayForward?objectId=2920	http://www.guidetopharmacology.org/GRAC/ObjectDisplayForward?objectId=2921	http://www.guidetopharmacology.org/GRAC/ObjectDisplayForward?objectId=2922
Common abbreviation	RIG‐1	MDA5	LGP2
HGNC, UniProt	https://www.genenames.org/data/gene‐symbol‐report/#!/hgnc_id/HGNC:19102, http://www.uniprot.org/uniprot/O95786	https://www.genenames.org/data/gene‐symbol‐report/#!/hgnc_id/HGNC:18873, http://www.uniprot.org/uniprot/Q9BYX4	https://www.genenames.org/data/gene‐symbol‐report/#!/hgnc_id/HGNC:29517, http://www.uniprot.org/uniprot/Q96C10

### Further reading on RIG‐I‐like receptor family

Chow KT *et al*. (2018) RIG‐I and Other RNA Sensors in Antiviral Immunity. *Annu. Rev. Immunol*. **36**: 667–694 https://www.ncbi.nlm.nih.gov/pubmed/29677479?dopt=AbstractPlus

Kato H *et al*. (2015) RIG‐I‐like receptors and autoimmune diseases. *Curr. Opin. Immunol*. **37**: 40–5 https://www.ncbi.nlm.nih.gov/pubmed/26530735?dopt=AbstractPlus

Lässig C *et al*. (2017) Discrimination of cytosolic self and non‐self RNA by RIG‐I‐like receptors. *J. Biol. Chem*. **292**: 9000‐9009 https://www.ncbi.nlm.nih.gov/pubmed/28411239?dopt=AbstractPlus

Ma Z *et al*. (2018) Innate Sensing of DNA Virus Genomes. *Annu Rev Virol* **5**: 341–362 https://www.ncbi.nlm.nih.gov/pubmed/30265633?dopt=AbstractPlus

Against Emerging and Re‐Emerging Viral Infections. *Front Immunol* **9**: 1379 https://www.ncbi.nlm.nih.gov/pubmed/29973930?dopt=AbstractPlus

### Further reading on Pattern recognition receptors

Broz P *et al*. (2016) Inflammasomes: mechanism of assembly, regulation and signalling. *Nat. Rev. Immunol*. **16**: 407–20 https://www.ncbi.nlm.nih.gov/pubmed/27291964?dopt=AbstractPlus

Bryant CE *et al*. (2015) Advances in Toll‐like receptor biology: Modes of activation by diverse stimuli. *Crit. Rev. Biochem. Mol. Biol*. **50**: 359–79 https://www.ncbi.nlm.nih.gov/pubmed/25857820?dopt=AbstractPlus

Feerick CL *et al*. (2017) Understanding the regulation of pattern recognition receptors in inflammatory diseases ‐ a ‘Nod’ in the right direction. *Immunology* **150**: 237–247 https://www.ncbi.nlm.nih.gov/pubmed/27706808?dopt=AbstractPlus

Rathinam VA *et al*. (2016) Inflammasome Complexes: Emerging Mechanisms and Effector Functions. *Cell* **165**: 792–800 https://www.ncbi.nlm.nih.gov/pubmed/27153493?dopt=AbstractPlus

Unterholzner L. (2013) The interferon response to intracellular DNA: why so many receptors? *Immunobiology* **218**: 1312–21 https://www.ncbi.nlm.nih.gov/pubmed/23962476?dopt=AbstractPlus

Yin Q *et al*. (2015) Structural biology of innate immunity. *Annu. Rev. Immunol*. **33**: 393–416 https://www.ncbi.nlm.nih.gov/pubmed/25622194?dopt=AbstractPlus

## http://www.guidetopharmacology.org/GRAC/FamilyDisplayForward?familyId=1022

### Overview

The mammalian genome encodes transmembrane and soluble receptor guanylyl cyclases, both of which have enzyme activities which convert http://www.guidetopharmacology.org/GRAC/LigandDisplayForward?ligandId=1742 to the intracellular second messenger cyclic guanosine‐3′,5′‐monophosphate (http://www.guidetopharmacology.org/GRAC/LigandDisplayForward?ligandId=2347).

## http://www.guidetopharmacology.org/GRAC/FamilyDisplayForward?familyId=662

### Overview

Transmembrane guanylyl cyclases are homodimeric receptors activated by a diverse range of endogenous ligands. GC‐A, GC‐B and GC‐C are expressed predominantly in the cardiovascular system, skeletal system and intestinal epithelium, respectively. GC‐D and GC‐G are found in the olfactory neuropepithelium and Grüeneberg ganglion of rodents, respectively. GC‐E and GC‐F are expressed in retinal photoreceptors. Family members have conserved ligand‐binding, catalytic (guanylyl cyclase) and regulatory domains with the exception of NPR‐C which has an extracellular binding domain homologous to that of other NPRs, but with a truncated intracellular domain which appears to couple, *via* the G_i/o_ family of Gproteins, to activation of phospholipase C, inwardly‐rectifying potassium channels and inhibition of adenylyl cyclase activity [http://www.ncbi.nlm.nih.gov/pubmed/10364194?dopt=AbstractPlus].

**Table:** 

Nomenclature	http://www.guidetopharmacology.org/GRAC/ObjectDisplayForward?objectId=1747	http://www.guidetopharmacology.org/GRAC/ObjectDisplayForward?objectId=1748	http://www.guidetopharmacology.org/GRAC/ObjectDisplayForward?objectId=1750	http://www.guidetopharmacology.org/GRAC/ObjectDisplayForward?objectId=1749
Common abbreviation	GC‐A	GC‐B	GC‐C	NPR‐C
HGNC, UniProt	https://www.genenames.org/data/gene‐symbol‐report/#!/hgnc_id/HGNC:7943, http://www.uniprot.org/uniprot/P16066	https://www.genenames.org/data/gene‐symbol‐report/#!/hgnc_id/HGNC:7944, http://www.uniprot.org/uniprot/P20594	https://www.genenames.org/data/gene‐symbol‐report/#!/hgnc_id/HGNC:4688, http://www.uniprot.org/uniprot/P25092	https://www.genenames.org/data/gene‐symbol‐report/#!/hgnc_id/HGNC:7945, http://www.uniprot.org/uniprot/P17342
Potency order	http://www.guidetopharmacology.org/GRAC/LigandDisplayForward?ligandId=4869 (https://www.genenames.org/data/gene‐symbol‐report/#!/hgnc_id/HGNC:7939, http://www.uniprot.org/uniprot/P01160) = http://www.guidetopharmacology.org/GRAC/LigandDisplayForward?ligandId=4890 (https://www.genenames.org/data/gene‐symbol‐report/#!/hgnc_id/HGNC:7940, http://www.uniprot.org/uniprot/P16860) ≫ http://www.guidetopharmacology.org/GRAC/LigandDisplayForward?ligandId=4896 (https://www.genenames.org/data/gene‐symbol‐report/#!/hgnc_id/HGNC:7941, http://www.uniprot.org/uniprot/P23582) [http://www.ncbi.nlm.nih.gov/pubmed/1309330?dopt=AbstractPlus]	http://www.guidetopharmacology.org/GRAC/LigandDisplayForward?ligandId=4896 (https://www.genenames.org/data/gene‐symbol‐report/#!/hgnc_id/HGNC:7941, http://www.uniprot.org/uniprot/P23582) ≫ http://www.guidetopharmacology.org/GRAC/LigandDisplayForward?ligandId=4869 (https://www.genenames.org/data/gene‐symbol‐report/#!/hgnc_id/HGNC:7939, http://www.uniprot.org/uniprot/P01160) ≫ http://www.guidetopharmacology.org/GRAC/LigandDisplayForward?ligandId=4890 (https://www.genenames.org/data/gene‐symbol‐report/#!/hgnc_id/HGNC:7940, http://www.uniprot.org/uniprot/P16860) [http://www.ncbi.nlm.nih.gov/pubmed/1309330?dopt=AbstractPlus]	http://www.guidetopharmacology.org/GRAC/LigandDisplayForward?ligandId=5084 (https://www.genenames.org/data/gene‐symbol‐report/#!/hgnc_id/HGNC:4683, http://www.uniprot.org/uniprot/Q16661) > http://www.guidetopharmacology.org/GRAC/LigandDisplayForward?ligandId=4945 (https://www.genenames.org/data/gene‐symbol‐report/#!/hgnc_id/HGNC:4682, http://www.uniprot.org/uniprot/Q02747)	http://www.guidetopharmacology.org/GRAC/LigandDisplayForward?ligandId=4869 (https://www.genenames.org/data/gene‐symbol‐report/#!/hgnc_id/HGNC:7939, http://www.uniprot.org/uniprot/P01160) > http://www.guidetopharmacology.org/GRAC/LigandDisplayForward?ligandId=4896 (https://www.genenames.org/data/gene‐symbol‐report/#!/hgnc_id/HGNC:7941, http://www.uniprot.org/uniprot/P23582) ≥ http://www.guidetopharmacology.org/GRAC/LigandDisplayForward?ligandId=4890 (https://www.genenames.org/data/gene‐symbol‐report/#!/hgnc_id/HGNC:7940, http://www.uniprot.org/uniprot/P16860) [http://www.ncbi.nlm.nih.gov/pubmed/1309330?dopt=AbstractPlus]
Endogenous agonists	http://www.guidetopharmacology.org/GRAC/LigandDisplayForward?ligandId=4869 (https://www.genenames.org/data/gene‐symbol‐report/#!/hgnc_id/HGNC:7939, http://www.uniprot.org/uniprot/P01160) (Binding) [http://www.ncbi.nlm.nih.gov/pubmed/8700153?dopt=AbstractPlus], http://www.guidetopharmacology.org/GRAC/LigandDisplayForward?ligandId=4890 (https://www.genenames.org/data/gene‐symbol‐report/#!/hgnc_id/HGNC:7940, http://www.uniprot.org/uniprot/P16860) (Binding) [http://www.ncbi.nlm.nih.gov/pubmed/8700153?dopt=AbstractPlus], http://www.guidetopharmacology.org/GRAC/LigandDisplayForward?ligandId=10211 [http://www.ncbi.nlm.nih.gov/pubmed/19729120?dopt=AbstractPlus]	http://www.guidetopharmacology.org/GRAC/LigandDisplayForward?ligandId=4896 (https://www.genenames.org/data/gene‐symbol‐report/#!/hgnc_id/HGNC:7941, http://www.uniprot.org/uniprot/P23582) (Binding) [http://www.ncbi.nlm.nih.gov/pubmed/1309330?dopt=AbstractPlus]	http://www.guidetopharmacology.org/GRAC/LigandDisplayForward?ligandId=4945 (https://www.genenames.org/data/gene‐symbol‐report/#!/hgnc_id/HGNC:4682, http://www.uniprot.org/uniprot/Q02747) (Binding), http://www.guidetopharmacology.org/GRAC/LigandDisplayForward?ligandId=5084 (https://www.genenames.org/data/gene‐symbol‐report/#!/hgnc_id/HGNC:4683, http://www.uniprot.org/uniprot/Q16661) (Binding)	–
Selective agonists	http://www.guidetopharmacology.org/GRAC/LigandDisplayForward?ligandId=9070 [http://www.ncbi.nlm.nih.gov/pubmed/16778132?dopt=AbstractPlus], http://www.guidetopharmacology.org/GRAC/LigandDisplayForward?ligandId=9067 [http://www.ncbi.nlm.nih.gov/pubmed/23154072?dopt=AbstractPlus], http://www.guidetopharmacology.org/GRAC/LigandDisplayForward?ligandId=9066 [http://www.ncbi.nlm.nih.gov/pubmed/23272242?dopt=AbstractPlus], http://www.guidetopharmacology.org/GRAC/LigandDisplayForward?ligandId=5053 [http://www.ncbi.nlm.nih.gov/pubmed/8700153?dopt=AbstractPlus]	http://www.guidetopharmacology.org/GRAC/LigandDisplayForward?ligandId=9066 [http://www.ncbi.nlm.nih.gov/pubmed/23272242?dopt=AbstractPlus], http://www.guidetopharmacology.org/GRAC/LigandDisplayForward?ligandId=9068 [http://www.ncbi.nlm.nih.gov/pubmed/23200862?dopt=AbstractPlus]	http://www.guidetopharmacology.org/GRAC/LigandDisplayForward?ligandId=5017 [http://www.ncbi.nlm.nih.gov/pubmed/20863829?dopt=AbstractPlus, http://www.ncbi.nlm.nih.gov/pubmed/17694454?dopt=AbstractPlus], http://www.guidetopharmacology.org/GRAC/LigandDisplayForward?ligandId=4854 [http://www.ncbi.nlm.nih.gov/pubmed/20863829?dopt=AbstractPlus], plecanatide [195]	http://www.guidetopharmacology.org/GRAC/LigandDisplayForward?ligandId=4892 [http://www.ncbi.nlm.nih.gov/pubmed/2823385?dopt=AbstractPlus]
Endogenous antagonists	–	–	–	http://www.guidetopharmacology.org/GRAC/LigandDisplayForward?ligandId=5036 (https://www.genenames.org/data/gene‐symbol‐report/#!/hgnc_id/HGNC:29961, http://www.uniprot.org/uniprot/P61366) [http://www.ncbi.nlm.nih.gov/pubmed/17951249?dopt=AbstractPlus]
Selective antagonists	http://www.guidetopharmacology.org/GRAC/LigandDisplayForward?ligandId=4856 (p*K*_i_ 9.2–9.5) [http://www.ncbi.nlm.nih.gov/pubmed/1680722?dopt=AbstractPlus], http://www.guidetopharmacology.org/GRAC/LigandDisplayForward?ligandId=4852 (p*K*_i_ 7.5) [http://www.ncbi.nlm.nih.gov/pubmed/2542088?dopt=AbstractPlus], http://www.guidetopharmacology.org/GRAC/LigandDisplayForward?ligandId=4952 [http://www.ncbi.nlm.nih.gov/pubmed/1674870?dopt=AbstractPlus], http://www.guidetopharmacology.org/GRAC/LigandDisplayForward?ligandId=4866 [http://www.ncbi.nlm.nih.gov/pubmed/1849131?dopt=AbstractPlus, http://www.ncbi.nlm.nih.gov/pubmed/1826288?dopt=AbstractPlus]	http://www.guidetopharmacology.org/GRAC/LigandDisplayForward?ligandId=9163 (p*K*_d_ 7.8) [http://www.ncbi.nlm.nih.gov/pubmed/15652659?dopt=AbstractPlus], http://www.guidetopharmacology.org/GRAC/LigandDisplayForward?ligandId=4952 [http://www.ncbi.nlm.nih.gov/pubmed/1674870?dopt=AbstractPlus], http://www.guidetopharmacology.org/GRAC/LigandDisplayForward?ligandId=4853 [http://www.ncbi.nlm.nih.gov/pubmed/15652659?dopt=AbstractPlus], http://www.guidetopharmacology.org/GRAC/LigandDisplayForward?ligandId=9162 [http://www.ncbi.nlm.nih.gov/pubmed/24297249?dopt=AbstractPlus]	–	http://www.guidetopharmacology.org/GRAC/LigandDisplayForward?ligandId=4870 (Binding) (p*K*_i_ 9.3) [http://www.ncbi.nlm.nih.gov/pubmed/10987424?dopt=AbstractPlus], http://www.guidetopharmacology.org/GRAC/LigandDisplayForward?ligandId=5020 [http://www.ncbi.nlm.nih.gov/pubmed/15337698?dopt=AbstractPlus]
Labelled ligands	http://www.guidetopharmacology.org/GRAC/LigandDisplayForward?ligandId=4845 (Agonist)	http://www.guidetopharmacology.org/GRAC/LigandDisplayForward?ligandId=4847	http://www.guidetopharmacology.org/GRAC/LigandDisplayForward?ligandId=4850 (Agonist) [http://www.ncbi.nlm.nih.gov/pubmed/9122260?dopt=AbstractPlus]	http://www.guidetopharmacology.org/GRAC/LigandDisplayForward?ligandId=4845

**Table:** 

Nomenclature	http://www.guidetopharmacology.org/GRAC/ObjectDisplayForward?objectId=2898	http://www.guidetopharmacology.org/GRAC/ObjectDisplayForward?objectId=2031	http://www.guidetopharmacology.org/GRAC/ObjectDisplayForward?objectId=2899	http://www.guidetopharmacology.org/GRAC/ObjectDisplayForward?objectId=2900
Common abbreviation	GC‐D	GC‐E	GC‐F	GC‐G
HGNC, UniProt	–	https://www.genenames.org/data/gene‐symbol‐report/#!/hgnc_id/HGNC:4689, http://www.uniprot.org/uniprot/Q02846	https://www.genenames.org/data/gene‐symbol‐report/#!/hgnc_id/HGNC:4691, http://www.uniprot.org/uniprot/P51841	https://www.genenames.org/data/gene‐symbol‐report/#!/hgnc_id/HGNC:31863, –
Localisation	–	Retinal photoreceptors	Retinal photoreceptors	Grüneberg ganglion
Principal function(s)	–	Vision/phototransduction	Vision/phototransduction	Thermosensation
Endogenous ligands	–	–	–	Cold
Comments	Pseudogene in humans	–	–	Pseudogene in humans

### Comments

The polysaccharide obtained from fermentation of *Aureobasidium* species, http://www.guidetopharmacology.org/GRAC/LigandDisplayForward?ligandId=4952, acts as an antagonist at both GC‐A and GC‐B receptor s [http://www.ncbi.nlm.nih.gov/pubmed/1674870?dopt=AbstractPlus]. GC‐D and GC‐G have been reported to be activated intracellularly by http://www.guidetopharmacology.org/GRAC/LigandDisplayForward?ligandId=9164 (https://www.genenames.org/data/gene‐symbol‐report/#!/hgnc_id/HGNC:4678, http://www.uniprot.org/uniprot/P43080) and guanylyl cyclaseactivating protein (https://www.genenames.org/data/gene‐symbol‐report/#!/hgnc_id/HGNC:4679, https://www.uniprot.org/uniprot/Q9UMX6). GC‐D and GC‐G may be activated by atmospheric levels of CO_2_ through the formation of intracellular bicarbonate ions [http://www.ncbi.nlm.nih.gov/pubmed/20738256?dopt=AbstractPlus, http://www.ncbi.nlm.nih.gov/pubmed/17702944?dopt=AbstractPlus]. GC‐G may be activated at cooler temperatures (20‐25°C) through apparent stabilisation of the dimer [http://www.ncbi.nlm.nih.gov/pubmed/25452496?dopt=AbstractPlus].

## http://www.guidetopharmacology.org/GRAC/FamilyDisplayForward?familyId=939

### Overview

Nitric oxide (http://www.guidetopharmacology.org/GRAC/LigandDisplayForward?ligandId=2509)‐sensitive (soluble) guanylyl cyclase (GTP diphosphate‐lyase (cyclising)), http://www.genome.jp/kegg‐bin/search_brite?option=‐a&search_string=4.6.1.2, is a heterodimer comprising a β_1_ subunit and one of two alpha subunits (α_1_, α_2_) giving rise to two functionally indistinguishable isoforms, GC‐1 (α_1_β_1_) and GC‐2 (α_2_β_1_) [http://www.ncbi.nlm.nih.gov/pubmed/9742221?dopt=AbstractPlus, http://www.ncbi.nlm.nih.gov/pubmed/9742212?dopt=AbstractPlus]. A haem group is associated with the β subunit and is the target for the endogenous ligand http://www.guidetopharmacology.org/GRAC/LigandDisplayForward?ligandId=2509, and, potentially, carbon monoxide [http://www.ncbi.nlm.nih.gov/pubmed/9003762?dopt=AbstractPlus].

**Table:** 

Nomenclature	http://www.guidetopharmacology.org/GRAC/ObjectDisplayForward?objectId=1287	http://www.guidetopharmacology.org/GRAC/ObjectDisplayForward?objectId=2897
Common abbreviation	GC‐1	GC‐2
Subunits	http://www.guidetopharmacology.org/GRAC/ObjectDisplayForward?objectId=1290, http://www.guidetopharmacology.org/GRAC/ObjectDisplayForward?objectId=1288	http://www.guidetopharmacology.org/GRAC/ObjectDisplayForward?objectId=1290, http://www.guidetopharmacology.org/GRAC/ObjectDisplayForward?objectId=1289
EC number	http://www.genome.jp/dbget‐bin/www_bget?ec:4.6.1.2	http://www.genome.jp/dbget‐bin/www_bget?ec:4.6.1.2
Endogenous ligands	http://www.guidetopharmacology.org/GRAC/LigandDisplayForward?ligandId=2509	http://www.guidetopharmacology.org/GRAC/LigandDisplayForward?ligandId=2509
Selective activators	http://www.guidetopharmacology.org/GRAC/LigandDisplayForward?ligandId=9900 (pEC_50_ 6.6) [http://www.ncbi.nlm.nih.gov/pubmed/29643251?dopt=AbstractPlus], http://www.guidetopharmacology.org/GRAC/LigandDisplayForward?ligandId=5291 [http://www.ncbi.nlm.nih.gov/pubmed/9003762?dopt=AbstractPlus, http://www.ncbi.nlm.nih.gov/pubmed/7527671?dopt=AbstractPlus, http://www.ncbi.nlm.nih.gov/pubmed/9742221?dopt=AbstractPlus], http://www.guidetopharmacology.org/GRAC/LigandDisplayForward?ligandId=5168 [apo‐GC‐1] [http://www.ncbi.nlm.nih.gov/pubmed/12086987?dopt=AbstractPlus], http://www.guidetopharmacology.org/GRAC/LigandDisplayForward?ligandId=10213 [http://www.ncbi.nlm.nih.gov/pubmed/29859918?dopt=AbstractPlus], http://www.guidetopharmacology.org/GRAC/LigandDisplayForward?ligandId=5257 [http://www.ncbi.nlm.nih.gov/pubmed/11242081?dopt=AbstractPlus, http://www.ncbi.nlm.nih.gov/pubmed/19089334?dopt=AbstractPlus]	http://www.guidetopharmacology.org/GRAC/LigandDisplayForward?ligandId=5291 [http://www.ncbi.nlm.nih.gov/pubmed/7527671?dopt=AbstractPlus, http://www.ncbi.nlm.nih.gov/pubmed/9742221?dopt=AbstractPlus], http://www.guidetopharmacology.org/GRAC/LigandDisplayForward?ligandId=5168 [apo‐GC‐2] [http://www.ncbi.nlm.nih.gov/pubmed/12086987?dopt=AbstractPlus], http://www.guidetopharmacology.org/GRAC/LigandDisplayForward?ligandId=10213 [http://www.ncbi.nlm.nih.gov/pubmed/29859918?dopt=AbstractPlus], http://www.guidetopharmacology.org/GRAC/LigandDisplayForward?ligandId=5257 [http://www.ncbi.nlm.nih.gov/pubmed/11242081?dopt=AbstractPlus, http://www.ncbi.nlm.nih.gov/pubmed/19089334?dopt=AbstractPlus]
Selective inhibitors	http://www.guidetopharmacology.org/GRAC/LigandDisplayForward?ligandId=6554 (pIC_50_ 8.1) [http://www.ncbi.nlm.nih.gov/pubmed/9489619?dopt=AbstractPlus] – Bovine, http://www.guidetopharmacology.org/GRAC/LigandDisplayForward?ligandId=5234 (pIC_50_ 7.5) [http://www.ncbi.nlm.nih.gov/pubmed/7544433?dopt=AbstractPlus]	http://www.guidetopharmacology.org/GRAC/LigandDisplayForward?ligandId=5234

**Table:** 

Nomenclature	http://www.guidetopharmacology.org/GRAC/ObjectDisplayForward?objectId=1288	http://www.guidetopharmacology.org/GRAC/ObjectDisplayForward?objectId=1289	http://www.guidetopharmacology.org/GRAC/ObjectDisplayForward?objectId=1290	http://www.guidetopharmacology.org/GRAC/ObjectDisplayForward?objectId=1291
HGNC, UniProt	https://www.genenames.org/data/gene‐symbol‐report/#!/hgnc_id/HGNC:4685, http://www.uniprot.org/uniprot/Q02108	https://www.genenames.org/data/gene‐symbol‐report/#!/hgnc_id/HGNC:4684, http://www.uniprot.org/uniprot/P33402	https://www.genenames.org/data/gene‐symbol‐report/#!/hgnc_id/HGNC:4687, http://www.uniprot.org/uniprot/Q02153	https://www.genenames.org/data/gene‐symbol‐report/#!/hgnc_id/HGNC:4686, http://www.uniprot.org/uniprot/O75343

### Comments

http://www.guidetopharmacology.org/GRAC/LigandDisplayForward?ligandId=5234 also shows activity at other haem‐containing proteins [http://www.ncbi.nlm.nih.gov/pubmed/10419542?dopt=AbstractPlus], while http://www.guidetopharmacology.org/GRAC/LigandDisplayForward?ligandId=5291 may also inhibit cGMP‐hydrolysing phosphodiesterases [http://www.ncbi.nlm.nih.gov/pubmed/9855623?dopt=AbstractPlus, http://www.ncbi.nlm.nih.gov/pubmed/10369473?dopt=AbstractPlus].

### Further reading on Receptor guanylyl cyclase (RGC) family

Kuhn M. (2016) Molecular Physiology of Membrane Guanylyl Cyclase Receptors. *Physiol. Rev*. **96**: 751‐804 https://www.ncbi.nlm.nih.gov/pubmed/27030537?dopt=AbstractPlus

Papapetropoulos A *et al*. (2015) Extending the translational potential of targeting NO/cGMP‐regulated pathways in the CVS. *Br. J. Pharmacol*. **172**: 1397–414 https://www.ncbi.nlm.nih.gov/pubmed/25302549?dopt=AbstractPlus

Santhekadur PK *et al*. (2017) The multifaceted role of natriuretic peptides in metabolic syndrome. *Biomed. Pharmacother*. **92**: 826–835 https://www.ncbi.nlm.nih.gov/pubmed/28599248?dopt=AbstractPlus

Vanhoutte PM *et al*. (2016) Thirty Years of Saying NO: Sources, Fate, Actions, and Misfortunes of the Endothelium‐Derived Vasodilator Mediator. *Circ. Res*. **119**: 375‐96 https://www.ncbi.nlm.nih.gov/pubmed/27390338?dopt=AbstractPlus

Volpe M *et al*. (2016) The natriuretic peptides system in the pathophysiology of heart failure: from molecular basis to treatment. *Clin. Sci*. **130**: 57–77 https://www.ncbi.nlm.nih.gov/pubmed/26637405?dopt=AbstractPlus

Waldman SA *et al*. (2018) Guanylate cyclase‐C as a therapeutic target in gastrointestinal disorders. *Gut* **67**: 1543–1552 https://www.ncbi.nlm.nih.gov/pubmed/29563144?dopt=AbstractPlus

## http://www.guidetopharmacology.org/GRAC/FamilyDisplayForward?familyId=304

### Overview

Receptor tyrosine kinases (RTKs), a family of cellsurface receptors, which transduce signals to polypeptide and protein hormones, cytokines and growth factors are key regulators of critical cellular processes, such as proliferation and differentiation, cell survival and metabolism, cell migration and cell cycle control [http://www.ncbi.nlm.nih.gov/pubmed/11357143?dopt=AbstractPlus, http://www.ncbi.nlm.nih.gov/pubmed/17575237?dopt=AbstractPlus, http://www.ncbi.nlm.nih.gov/pubmed/2158859?dopt=AbstractPlus]. In the human genome, 58 RTKs have been identified, which fall into 20 families [http://www.ncbi.nlm.nih.gov/pubmed/20602996?dopt=AbstractPlus].

All RTKs display an extracellular ligand binding domain, a single transmembrane helix, a cytoplasmic region containing the protein tyrosine kinase activity (occasionally split into two domains by an insertion, termed the kinase insertion), with juxtamembrane and C‐terminal regulatory regions. Agonist binding to the extracellular domain evokes dimerization, and sometimes oligomerization, of RTKs (a small subset of RTKs forms multimers even in the absence of activating ligand). This leads to autophosphorylation in the tyrosine kinase domain in a trans orientation, serving as a site of assembly of protein complexes and stimulation of multiple signal transduction pathways, including http://www.guidetopharmacology.org/GRAC/FamilyDisplayForward?familyId=244, http://www.guidetopharmacology.org/GRAC/FamilyDisplayForward?familyId=246#mitogen‐activated protein kinases and phosphatidylinositol 3‐kinase [http://www.ncbi.nlm.nih.gov/pubmed/2158859?dopt=AbstractPlus].

RTKs are of widespread interest not only through physiological functions, but also as drug targets in many types of cancer and other disease states. Many diseases result from genetic changes or abnormalities that either alter the activity, abundance, cellular distribution and/or regulation of RTKs. Therefore, drugs thatmodify the dysregulated functions of these RTKs have been developed which fall into two categories. One group is often described as ‘biologicals’, which block the activation of RTKs directly or by chelating the cognate ligands, while the second are small molecules designed to inhibit the tyrosine kinase activity directly.

## http://www.guidetopharmacology.org/GRAC/FamilyDisplayForward?familyId=320

### Overview

http://www.ensembl.org/Homo_sapiens/Gene/Family/Genes?family=ENSFM00500000269785 are Class I receptor tyrosine kinases [http://www.ncbi.nlm.nih.gov/pubmed/12520021?dopt=AbstractPlus]. https://www.genenames.org/data/gene‐symbol‐report/#!/hgnc_id/HGNC:3430 (also known as HER‐2 or NEU) appears to act as an essential partner for the other members of the family without itself being activated by a cognate ligand [http://www.ncbi.nlm.nih.gov/pubmed/9130710?dopt=AbstractPlus]. Ligands of the ErbB family of receptors are peptides, many of which are generated by proteolytic cleavage of cell‐surface proteins. HER/ErbB is the viral counterpart to the receptor tyrosine kinase EGFR. All family members heterodimerize with each other to activate downstream signalling pathways and are aberrantly expressed inmany cancers, particularly forms of breast cancer and lung cancer. Mutations in the EGFR are responsible for acquired resistance to tyrosine kinase inhibitor chemotherapeutics.

**Table:** 

Nomenclature	http://www.guidetopharmacology.org/GRAC/ObjectDisplayForward?objectId=1797	http://www.guidetopharmacology.org/GRAC/ObjectDisplayForward?objectId=2019	http://www.guidetopharmacology.org/GRAC/ObjectDisplayForward?objectId=1798	http://www.guidetopharmacology.org/GRAC/ObjectDisplayForward?objectId=1799
Common abbreviation	EGFR	HER2	HER3	HER4
HGNC, UniProt	https://www.genenames.org/data/gene‐symbol‐report/#!/hgnc_id/HGNC:3236, http://www.uniprot.org/uniprot/P00533	https://www.genenames.org/data/gene‐symbol‐report/#!/hgnc_id/HGNC:3430, http://www.uniprot.org/uniprot/P04626	https://www.genenames.org/data/gene‐symbol‐report/#!/hgnc_id/HGNC:3431, http://www.uniprot.org/uniprot/P21860	https://www.genenames.org/data/gene‐symbol‐report/#!/hgnc_id/HGNC:3432, http://www.uniprot.org/uniprot/Q15303
EC number	http://www.genome.jp/dbget‐bin/www_bget?ec:2.7.10.1	http://www.genome.jp/dbget‐bin/www_bget?ec:2.7.10.1	http://www.genome.jp/dbget‐bin/www_bget?ec:2.7.10.1	http://www.genome.jp/dbget‐bin/www_bget?ec:2.7.10.1
Endogenous ligands	http://www.guidetopharmacology.org/GRAC/LigandDisplayForward?ligandId=4916 (http://www.guidetopharmacology.org/GRAC/LigandDisplayForward?ligandId=4916, http://www.uniprot.org/uniprot/P01133) (Binding), http://www.guidetopharmacology.org/GRAC/LigandDisplayForward?ligandId=4948 (https://www.genenames.org/data/gene‐symbol‐report/#!/hgnc_id/HGNC:3059, http://www.uniprot.org/uniprot/Q99075) (Binding), http://www.guidetopharmacology.org/GRAC/LigandDisplayForward?ligandId=5059 (https://www.genenames.org/data/gene‐symbol‐report/#!/hgnc_id/HGNC:11765, http://www.uniprot.org/uniprot/P01135) (Binding), http://www.guidetopharmacology.org/GRAC/LigandDisplayForward?ligandId=4864 (https://www.genenames.org/data/gene‐symbol‐report/#!/hgnc_id/HGNC:651, http://www.uniprot.org/uniprot/P15514) (Binding), http://www.guidetopharmacology.org/GRAC/LigandDisplayForward?ligandId=4873 (https://www.genenames.org/data/gene‐symbol‐report/#!/hgnc_id/HGNC:1121, http://www.uniprot.org/uniprot/P35070) (Binding), http://www.guidetopharmacology.org/GRAC/LigandDisplayForward?ligandId=4917 (https://www.genenames.org/data/gene‐symbol‐report/#!/hgnc_id/HGNC:17470, http://www.uniprot.org/uniprot/Q6UW88) (Binding), http://www.guidetopharmacology.org/GRAC/LigandDisplayForward?ligandId=4918 (https://www.genenames.org/data/gene‐symbol‐report/#!/hgnc_id/HGNC:3443, http://www.uniprot.org/uniprot/O14944) (Binding)	–	http://www.guidetopharmacology.org/GRAC/LigandDisplayForward?ligandId=5027 (https://www.genenames.org/data/gene‐symbol‐report/#!/hgnc_id/HGNC:7997, http://www.uniprot.org/uniprot/Q02297), http://www.guidetopharmacology.org/GRAC/LigandDisplayForward?ligandId=5029 (https://www.genenames.org/data/gene‐symbol‐report/#!/hgnc_id/HGNC:7998, http://www.uniprot.org/uniprot/O14511)	http://www.guidetopharmacology.org/GRAC/LigandDisplayForward?ligandId=4948 (https://www.genenames.org/data/gene‐symbol‐report/#!/hgnc_id/HGNC:3059, http://www.uniprot.org/uniprot/Q99075), http://www.guidetopharmacology.org/GRAC/LigandDisplayForward?ligandId=4873 (https://www.genenames.org/data/gene‐symbol‐report/#!/hgnc_id/HGNC:1121, http://www.uniprot.org/uniprot/P35070), http://www.guidetopharmacology.org/GRAC/LigandDisplayForward?ligandId=4918 (https://www.genenames.org/data/gene‐symbol‐report/#!/hgnc_id/HGNC:3443, http://www.uniprot.org/uniprot/O14944), http://www.guidetopharmacology.org/GRAC/LigandDisplayForward?ligandId=5027 (https://www.genenames.org/data/gene‐symbol‐report/#!/hgnc_id/HGNC:7997, http://www.uniprot.org/uniprot/Q02297), http://www.guidetopharmacology.org/GRAC/LigandDisplayForward?ligandId=5029 (https://www.genenames.org/data/gene‐symbol‐report/#!/hgnc_id/HGNC:7998, http://www.uniprot.org/uniprot/O14511), http://www.guidetopharmacology.org/GRAC/LigandDisplayForward?ligandId=5030 (https://www.genenames.org/data/gene‐symbol‐report/#!/hgnc_id/HGNC:7999, http://www.uniprot.org/uniprot/P56975), http://www.guidetopharmacology.org/GRAC/LigandDisplayForward?ligandId=5031 (https://www.genenames.org/data/gene‐symbol‐report/#!/hgnc_id/HGNC:29862, http://www.uniprot.org/uniprot/Q8WWG1)
Inhibitors	http://www.guidetopharmacology.org/GRAC/LigandDisplayForward?ligandId=5667 (p*K*_d_ 9.6) [http://www.ncbi.nlm.nih.gov/pubmed/22037378?dopt=AbstractPlus], http://www.guidetopharmacology.org/GRAC/LigandDisplayForward?ligandId=7944 (pIC_50_ 9.5) [http://www.ncbi.nlm.nih.gov/pubmed/17575237?dopt=AbstractPlus], http://www.guidetopharmacology.org/GRAC/LigandDisplayForward?ligandId=5667 (pIC_50_ 8–9.3) [http://www.ncbi.nlm.nih.gov/pubmed/20550212?dopt=AbstractPlus, http://www.ncbi.nlm.nih.gov/pubmed/18408761?dopt=AbstractPlus]	http://www.guidetopharmacology.org/GRAC/LigandDisplayForward?ligandId=7903 (pIC_50_ 8.3) [http://www.ncbi.nlm.nih.gov/pubmed/21306821?dopt=AbstractPlus], http://www.guidetopharmacology.org/GRAC/LigandDisplayForward?ligandId=7883 (pIC_50_ 7.9) [http://www.ncbi.nlm.nih.gov/pubmed/18500794?dopt=AbstractPlus], http://www.guidetopharmacology.org/GRAC/LigandDisplayForward?ligandId=7944 (pIC_50_ 7.8) [http://www.ncbi.nlm.nih.gov/pubmed/17575237?dopt=AbstractPlus], http://www.guidetopharmacology.org/GRAC/LigandDisplayForward?ligandId=7879 (pIC_50_ 7.7) [http://www.ncbi.nlm.nih.gov/pubmed/20166197?dopt=AbstractPlus]	–	http://www.guidetopharmacology.org/GRAC/LigandDisplayForward?ligandId=7903 (pIC_50_ 7.6) [http://www.ncbi.nlm.nih.gov/pubmed/21306821?dopt=AbstractPlus]
Antibodies	http://www.guidetopharmacology.org/GRAC/LigandDisplayForward?ligandId=8090 (Binding) (p*K*_d_ 9.5) [134], http://www.guidetopharmacology.org/GRAC/LigandDisplayForward?ligandId=6882 (Binding) (p*K*_d_ 9.4) [70]	http://www.guidetopharmacology.org/GRAC/LigandDisplayForward?ligandId=5046 (Inhibition) (pIC_50_ > 8) [106], http://www.guidetopharmacology.org/GRAC/LigandDisplayForward?ligandId=5082 (Inhibition)	–	–

### Comments

http://www.guidetopharmacology.org/GRAC/LigandDisplayForward?ligandId=4848 has been used to label the ErbB1 EGF receptor. The extracellular domain of ErbB2 can be targetted by the antibodies http://www.guidetopharmacology.org/GRAC/LigandDisplayForward?ligandId=5082 and http://www.guidetopharmacology.org/GRAC/LigandDisplayForward?ligandId=5046 to inhibit ErbB family action. The intracellular ATP‐binding site of the tyrosine kinase domain can be inhibited by http://www.guidetopharmacology.org/GRAC/LigandDisplayForward?ligandId=4947 (7.9‐8.0, [http://www.ncbi.nlm.nih.gov/pubmed/12639547?dopt=AbstractPlus]), http://www.guidetopharmacology.org/GRAC/LigandDisplayForward?ligandId=4941, http://www.guidetopharmacology.org/GRAC/LigandDisplayForward?ligandId=4920 and tyrphostins http://www.guidetopharmacology.org/GRAC/LigandDisplayForward?ligandId=4863 and http://www.guidetopharmacology.org/GRAC/LigandDisplayForward?ligandId=4862.

### Further reading on Type I RTKs: ErbB (epidermal growth factor) receptor family

Kobayashi Y *et al*. (2016) Not all epidermal growth factor receptor mutations in lung cancer are created equal: Perspectives for individualized treatment strategy. *Cancer Sci*. **107**: 1179‐86 https://www.ncbi.nlm.nih.gov/pubmed/27323238?dopt=AbstractPlus

## http://www.guidetopharmacology.org/GRAC/FamilyDisplayForward?familyId=321

### Overview

The circulating peptide hormones http://www.guidetopharmacology.org/GRAC/LigandDisplayForward?ligandId=5012 (https://www.genenames.org/data/gene‐symbol‐report/#!/hgnc_id/HGNC:6081, http://www.uniprot.org/uniprot/P01308) and the related insulin‐like growth factors (IGF) activate Class II receptor tyrosine kinases [http://www.ncbi.nlm.nih.gov/pubmed/12520021?dopt=AbstractPlus], to evoke cellular responses, mediated through multiple intracellular adaptor proteins. Exceptionally amongst the catalytic receptors, the functional receptor in the insulin receptor family is derived from a single gene product, cleaved post‐translationally into two peptides, which then cross‐link via disulphide bridges to form a heterotetramer. Intriguingly, the endogenous peptide ligands are formed in a parallel fashion with post‐translational processing producing a heterodimer linked by disulphide bridges. Signalling through the receptors is mediated through a rapid autophosphorylation event at intracellular tyrosine residues, followed by recruitment of multiple adaptor proteins, notably https://www.genenames.org/data/gene‐symbol‐report/#!/hgnc_id/HGNC:6125 (http://www.uniprot.org/uniprot/P35568), https://www.genenames.org/data/gene‐symbol‐report/#!/hgnc_id/HGNC:6126 (http://www.uniprot.org/uniprot/Q9Y4H2), https://www.genenames.org/data/gene‐symbol‐report/#!/hgnc_id/HGNC:10840 (http://www.uniprot.org/uniprot/P29353), https://www.genenames.org/data/gene‐symbol‐report/#!/hgnc_id/HGNC:4566 (http://www.uniprot.org/uniprot/P62993) and https://www.genenames.org/data/gene‐symbol‐report/#!/hgnc_id/HGNC:11187 (http://www.uniprot.org/uniprot/Q07889).

Serum levels of free IGFs are kept low by the action of IGF binding proteins (IGFBP1‐5, http://www.uniprot.org/uniprot/P08833, http://www.uniprot.org/uniprot/P18065, http://www.uniprot.org/uniprot/P17936, http://www.uniprot.org/uniprot/P22692, http://www.uniprot.org/uniprot/P24593), which sequester the IGFs; overexpression of IGFBPs may induce apoptosis, while IGFBP levels are also altered in some cancers.

**Table:** 

Nomenclature	http://www.guidetopharmacology.org/GRAC/ObjectDisplayForward?objectId=1800	http://www.guidetopharmacology.org/GRAC/ObjectDisplayForward?objectId=1801	http://www.guidetopharmacology.org/GRAC/ObjectDisplayForward?objectId=1802
Common abbreviation	InsR	IGF1R	IRR
HGNC, UniProt	https://www.genenames.org/data/gene‐symbol‐report/#!/hgnc_id/HGNC:6091, http://www.uniprot.org/uniprot/P06213	https://www.genenames.org/data/gene‐symbol‐report/#!/hgnc_id/HGNC:5465, http://www.uniprot.org/uniprot/P08069	https://www.genenames.org/data/gene‐symbol‐report/#!/hgnc_id/HGNC:6093, http://www.uniprot.org/uniprot/P14616
EC number	http://www.genome.jp/dbget‐bin/www_bget?ec:2.7.10.1	http://www.genome.jp/dbget‐bin/www_bget?ec:2.7.10.1	http://www.genome.jp/dbget‐bin/www_bget?ec:2.7.10.1
Inhibitors	–	http://www.guidetopharmacology.org/GRAC/LigandDisplayForward?ligandId=7952 (pIC_50_ 8.7) [http://www.ncbi.nlm.nih.gov/pubmed/19778024?dopt=AbstractPlus], http://www.guidetopharmacology.org/GRAC/LigandDisplayForward?ligandId=5683 (pIC_50_ 8.7) [http://www.ncbi.nlm.nih.gov/pubmed/19825801?dopt=AbstractPlus], http://www.guidetopharmacology.org/GRAC/LigandDisplayForward?ligandId=5683 (p*K*_d_ 8.1) [http://www.ncbi.nlm.nih.gov/pubmed/22037378?dopt=AbstractPlus], http://www.guidetopharmacology.org/GRAC/LigandDisplayForward?ligandId=5048 (pIC_50_ > 6) [http://www.ncbi.nlm.nih.gov/pubmed/16648580?dopt=AbstractPlus], http://www.guidetopharmacology.org/GRAC/LigandDisplayForward?ligandId=5912 (pIC_50_ 4.7) [http://www.ncbi.nlm.nih.gov/pubmed/9075698?dopt=AbstractPlus]	–
Selective inhibitors	–	http://www.guidetopharmacology.org/GRAC/LigandDisplayForward?ligandId=8067 (pIC_50_ 9.4) [http://www.ncbi.nlm.nih.gov/pubmed/15050915?dopt=AbstractPlus]	–
Endogenous agonists	http://www.guidetopharmacology.org/GRAC/LigandDisplayForward?ligandId=5012 (https://www.genenames.org/data/gene‐symbol‐report/#!/hgnc_id/HGNC:6081, http://www.uniprot.org/uniprot/P01308)	http://www.guidetopharmacology.org/GRAC/LigandDisplayForward?ligandId=4971 (https://www.genenames.org/data/gene‐symbol‐report/#!/hgnc_id/HGNC:5464, http://www.uniprot.org/uniprot/P05019), http://www.guidetopharmacology.org/GRAC/LigandDisplayForward?ligandId=4972 (https://www.genenames.org/data/gene‐symbol‐report/#!/hgnc_id/HGNC:5466, http://www.uniprot.org/uniprot/P01344)	–

### Comments

There is evidence for low potency binding and activation of insulin receptors by IGF1. IGF2 also binds and activates the cation‐independent mannose 6‐phosphate receptor (also known as the insulin‐like growth factor 2 receptor; https://www.genenames.org/data/gene‐symbol‐report/#!/hgnc_id/HGNC:5467; http://www.uniprot.org/uniprot/P11717), which lacks classical signalling capacity and appears to subserve a trafficking role [http://www.ncbi.nlm.nih.gov/pubmed/2964083?dopt=AbstractPlus]. INSRR, which has a much more discrete localization, being predominant in the kidney [http://www.ncbi.nlm.nih.gov/pubmed/1530648?dopt=AbstractPlus], currently lacks a cognate ligand or evidence for functional impact. Antibodies targetting IGF1, IGF2 and the extracellular portion of the IGF1 receptor are in clinical trials.

http://www.guidetopharmacology.org/GRAC/LigandDisplayForward?ligandId=5048 inhibits the insulin‐like growth factor receptor [5], while BMS‐536924 inhibits both the insulin receptor and the insulinlike growth factor receptor [http://www.ncbi.nlm.nih.gov/pubmed/16134929?dopt=AbstractPlus].

## http://www.guidetopharmacology.org/GRAC/FamilyDisplayForward?familyId=322

### Overview

Type III RTKs include PDGFR, CSF‐1R (Ems), Kit and FLT3, which function as homo‐ or heterodimers. Endogenous ligands of PDGF receptors are homo‐ or heterodimeric: PDGFA, PDGFB, VEGFE and http://www.guidetopharmacology.org/GRAC/LigandDisplayForward?ligandId=5323 (https://www.genenames.org/data/gene‐symbol‐report/#!/hgnc_id/HGNC:30620, http://www.uniprot.org/uniprot/Q9GZP0) combine as homo‐ or heterodimers to activate homo‐ or heterodimeric PDGF receptors. SCF is a dimeric ligand for KIT. Ligands for CSF1R are either monomeric or dimeric glycoproteins, while the endogenous agonist for FLT3 is a homodimer.

**Table:** 

Nomenclature	http://www.guidetopharmacology.org/GRAC/ObjectDisplayForward?objectId=1803	http://www.guidetopharmacology.org/GRAC/ObjectDisplayForward?objectId=1804	http://www.guidetopharmacology.org/GRAC/ObjectDisplayForward?objectId=1805
Common abbreviation	PDGFRα	PDGFRβ	Kit
HGNC, UniProt	https://www.genenames.org/data/gene‐symbol‐report/#!/hgnc_id/HGNC:8803, http://www.uniprot.org/uniprot/P16234	https://www.genenames.org/data/gene‐symbol‐report/#!/hgnc_id/HGNC:8804, http://www.uniprot.org/uniprot/P09619	https://www.genenames.org/data/gene‐symbol‐report/#!/hgnc_id/HGNC:6342, http://www.uniprot.org/uniprot/P10721
EC number	http://www.genome.jp/dbget‐bin/www_bget?ec:2.7.10.1	http://www.genome.jp/dbget‐bin/www_bget?ec:2.7.10.1	http://www.genome.jp/dbget‐bin/www_bget?ec:2.7.10.1
Endogenous ligands	PDGF	PDGF	–
Inhibitors	http://www.guidetopharmacology.org/GRAC/LigandDisplayForward?ligandId=8013 (pIC_50_ 8.7) [http://www.ncbi.nlm.nih.gov/pubmed/18849971?dopt=AbstractPlus], http://www.guidetopharmacology.org/GRAC/LigandDisplayForward?ligandId=7882 (p*K*_d_ 8.7) [http://www.ncbi.nlm.nih.gov/pubmed/22745105?dopt=AbstractPlus], http://www.guidetopharmacology.org/GRAC/LigandDisplayForward?ligandId=7885 (pIC_50_ 7.2) [http://www.ncbi.nlm.nih.gov/pubmed/19320489?dopt=AbstractPlus]	http://www.guidetopharmacology.org/GRAC/LigandDisplayForward?ligandId=7882 (p*K*_d_ 8.5) [http://www.ncbi.nlm.nih.gov/pubmed/22745105?dopt=AbstractPlus], http://www.guidetopharmacology.org/GRAC/LigandDisplayForward?ligandId=5712 (pIC_50_ 8.4) [http://www.ncbi.nlm.nih.gov/pubmed/16891463?dopt=AbstractPlus], http://www.guidetopharmacology.org/GRAC/LigandDisplayForward?ligandId=7886 (pIC_50_ 8.4) [http://www.ncbi.nlm.nih.gov/pubmed/21028894?dopt=AbstractPlus], http://www.guidetopharmacology.org/GRAC/LigandDisplayForward?ligandId=5713 (pIC_50_ 8.2) [http://www.ncbi.nlm.nih.gov/pubmed/23279183?dopt=AbstractPlus], http://www.guidetopharmacology.org/GRAC/LigandDisplayForward?ligandId=5713 (p*K*_i_ 8.1) [http://www.ncbi.nlm.nih.gov/pubmed/12538485?dopt=AbstractPlus]	http://www.guidetopharmacology.org/GRAC/LigandDisplayForward?ligandId=5713 (p*K*_d_ 9.4) [http://www.ncbi.nlm.nih.gov/pubmed/22037378?dopt=AbstractPlus], http://www.guidetopharmacology.org/GRAC/LigandDisplayForward?ligandId=7886 (pIC_50_ 8.7) [http://www.ncbi.nlm.nih.gov/pubmed/21028894?dopt=AbstractPlus], http://www.guidetopharmacology.org/GRAC/LigandDisplayForward?ligandId=5656 (p*K*_d_ 8.1) [http://www.ncbi.nlm.nih.gov/pubmed/22037378?dopt=AbstractPlus], http://www.guidetopharmacology.org/GRAC/LigandDisplayForward?ligandId=5712 (pIC_50_ 7.8) [http://www.ncbi.nlm.nih.gov/pubmed/16891463?dopt=AbstractPlus], http://www.guidetopharmacology.org/GRAC/LigandDisplayForward?ligandId=7969 (pIC_50_ 7.5) [http://www.ncbi.nlm.nih.gov/pubmed/22864397?dopt=AbstractPlus], http://www.guidetopharmacology.org/GRAC/LigandDisplayForward?ligandId=5711 (pIC_50_ 7.2) [http://www.ncbi.nlm.nih.gov/pubmed/15466206?dopt=AbstractPlus]
Selective inhibitors	http://www.guidetopharmacology.org/GRAC/LigandDisplayForward?ligandId=8069 (pIC_50_ 8) [http://www.ncbi.nlm.nih.gov/pubmed/15705896?dopt=AbstractPlus]	http://www.guidetopharmacology.org/GRAC/LigandDisplayForward?ligandId=8069 (pIC_50_ 9) [http://www.ncbi.nlm.nih.gov/pubmed/15705896?dopt=AbstractPlus]	–
Endogenous agonists	–	–	http://www.guidetopharmacology.org/GRAC/LigandDisplayForward?ligandId=5055 (https://www.genenames.org/data/gene‐symbol‐report/#!/hgnc_id/HGNC:6343, http://www.uniprot.org/uniprot/P21583) [http://www.ncbi.nlm.nih.gov/pubmed/7536489?dopt=AbstractPlus]

**Table:** 

Nomenclature	http://www.guidetopharmacology.org/GRAC/ObjectDisplayForward?objectId=1806	http://www.guidetopharmacology.org/GRAC/ObjectDisplayForward?objectId=1807
Common abbreviation	CSFR	FLT3
HGNC, UniProt	https://www.genenames.org/data/gene‐symbol‐report/#!/hgnc_id/HGNC:2433, http://www.uniprot.org/uniprot/P07333	https://www.genenames.org/data/gene‐symbol‐report/#!/hgnc_id/HGNC:3765, http://www.uniprot.org/uniprot/P36888
EC number	http://www.genome.jp/dbget‐bin/www_bget?ec:2.7.10.1	http://www.genome.jp/dbget‐bin/www_bget?ec:2.7.10.1
Endogenous ligands	http://www.guidetopharmacology.org/GRAC/LigandDisplayForward?ligandId=4934 (https://www.genenames.org/data/gene‐symbol‐report/#!/hgnc_id/HGNC:2438, http://www.uniprot.org/uniprot/P09919), http://www.guidetopharmacology.org/GRAC/LigandDisplayForward?ligandId=4942 (https://www.genenames.org/data/gene‐symbol‐report/#!/hgnc_id/HGNC:2434, http://www.uniprot.org/uniprot/P04141), http://www.guidetopharmacology.org/GRAC/LigandDisplayForward?ligandId=4905 (https://www.genenames.org/data/gene‐symbol‐report/#!/hgnc_id/HGNC:2432, http://www.uniprot.org/uniprot/P09603)	http://www.guidetopharmacology.org/GRAC/LigandDisplayForward?ligandId=4932 (https://www.genenames.org/data/gene‐symbol‐report/#!/hgnc_id/HGNC:3766, http://www.uniprot.org/uniprot/P49771)
Inhibitors	http://www.guidetopharmacology.org/GRAC/LigandDisplayForward?ligandId=5689 (pIC_50_ 9.2) [http://www.ncbi.nlm.nih.gov/pubmed/19887542?dopt=AbstractPlus], http://www.guidetopharmacology.org/GRAC/LigandDisplayForward?ligandId=5690 (p*K*_d_ 9.1) [http://www.ncbi.nlm.nih.gov/pubmed/22037378?dopt=AbstractPlus], http://www.guidetopharmacology.org/GRAC/LigandDisplayForward?ligandId=5690 (pIC_50_ 8.7) [http://www.ncbi.nlm.nih.gov/pubmed/17121910?dopt=AbstractPlus], http://www.guidetopharmacology.org/GRAC/LigandDisplayForward?ligandId=5685 (p*K*_d_ 8.7) [http://www.ncbi.nlm.nih.gov/pubmed/22037378?dopt=AbstractPlus], http://www.guidetopharmacology.org/GRAC/LigandDisplayForward?ligandId=5689 (p*K*_d_ 8.5) [http://www.ncbi.nlm.nih.gov/pubmed/22037378?dopt=AbstractPlus]	http://www.guidetopharmacology.org/GRAC/LigandDisplayForward?ligandId=8095 (p*K*_d_ 9.3) [http://www.ncbi.nlm.nih.gov/pubmed/24900421?dopt=AbstractPlus], http://www.guidetopharmacology.org/GRAC/LigandDisplayForward?ligandId=5657 (p*K*_d_ 9.2) [http://www.ncbi.nlm.nih.gov/pubmed/22037378?dopt=AbstractPlus], http://www.guidetopharmacology.org/GRAC/LigandDisplayForward?ligandId=7882 (p*K*_d_ 9.1) [http://www.ncbi.nlm.nih.gov/pubmed/22745105?dopt=AbstractPlus], http://www.guidetopharmacology.org/GRAC/LigandDisplayForward?ligandId=7885 (pIC_50_ 8.5) [http://www.ncbi.nlm.nih.gov/pubmed/19320489?dopt=AbstractPlus], http://www.guidetopharmacology.org/GRAC/LigandDisplayForward?ligandId=5695 (p*K*_d_ 8.5) [http://www.ncbi.nlm.nih.gov/pubmed/22037378?dopt=AbstractPlus], http://www.guidetopharmacology.org/GRAC/LigandDisplayForward?ligandId=5695 (pIC_50_ 6.7) [http://www.ncbi.nlm.nih.gov/pubmed/12124172?dopt=AbstractPlus]
Selective inhibitors	http://www.guidetopharmacology.org/GRAC/LigandDisplayForward?ligandId=5685 (pIC_50_ 7.2) [http://www.ncbi.nlm.nih.gov/pubmed/16249345?dopt=AbstractPlus]	http://www.guidetopharmacology.org/GRAC/LigandDisplayForward?ligandId=8108 (pIC_50_ 9.4) [http://www.ncbi.nlm.nih.gov/pubmed/24532805?dopt=AbstractPlus]
Comments	Upregulation of CSF1R expression is associated with migroglial activation and immune pathology in Alzhermer&apos;s disease (AD) [71, http://www.ncbi.nlm.nih.gov/pubmed/23392676?dopt=AbstractPlus]. Pharmacological inhibition of CSF1R with http://www.guidetopharmacology.org/GRAC/LigandDisplayForward?ligandId=5685 reduces microglial proliferation and prevents disease progression in a mouse model of AD, but this does not correlate with amyloid‐β plaque numbers [http://www.ncbi.nlm.nih.gov/pubmed/26747862?dopt=AbstractPlus].	http://www.guidetopharmacology.org/GRAC/LigandDisplayForward?ligandId=4855 has been described as a selective FLT3 inhibitor [http://www.ncbi.nlm.nih.gov/pubmed/20153646?dopt=AbstractPlus].

### Comments

Various small molecular inhibitors of type III RTKs have been described, including http://www.guidetopharmacology.org/GRAC/LigandDisplayForward?ligandId=5687 and http://www.guidetopharmacology.org/GRAC/LigandDisplayForward?ligandId=5697 (targetting PDGFR, KIT and CSF1R); http://www.guidetopharmacology.org/GRAC/LigandDisplayForward?ligandId=5702 and AC220 (http://www.guidetopharmacology.org/GRAC/LigandDisplayForward?ligandId=5658; FLT3), as well as pan‐type III RTK inhibitors such as http://www.guidetopharmacology.org/GRAC/LigandDisplayForward?ligandId=5713 and http://www.guidetopharmacology.org/GRAC/LigandDisplayForward?ligandId=5711 [http://www.ncbi.nlm.nih.gov/pubmed/15606337?dopt=AbstractPlus]; 5′‐fluoroindirubinoxime has been described as a selective FLT3 inhibitor [2].

## http://www.guidetopharmacology.org/GRAC/FamilyDisplayForward?familyId=324

### Overview

http://www.ensembl.org/Homo_sapiens/Gene/Family/Genes?family=ENSFM00440000236870 are homo‐ and heterodimeric proteins, which are characterized by seven Ig‐like loops in their extracellular domains and a split kinase domain in the cytoplasmic region. They are key regulators of angiogenesis and lymphangiogenesis; as such, they have been the focus of drug discovery for conditions such as metastatic cancer. Splice variants of VEGFR1 and VEGFR2 generate truncated proteins limited to the extracellular domains, capable of homodimerisation and binding VEGF ligands as a soluble, non‐signalling entity. Ligands at VEGF receptors are typically homodimeric. http://www.guidetopharmacology.org/GRAC/LigandDisplayForward?ligandId=5085 (https://www.genenames.org/data/gene‐symbol‐report/#!/hgnc_id/HGNC:12680, http://www.uniprot.org/uniprot/P15692) is able to activate VEGFR1 homodimers, VEGFR1/2 heterodimers and VEGFR2/3 heterodimers. http://www.guidetopharmacology.org/GRAC/LigandDisplayForward?ligandId=5086 (https://www.genenames.org/data/gene‐symbol‐report/#!/hgnc_id/HGNC:12681, http://www.uniprot.org/uniprot/P49765) and http://www.guidetopharmacology.org/GRAC/LigandDisplayForward?ligandId=5314 (https://www.genenames.org/data/gene‐symbol‐report/#!/hgnc_id/HGNC:8893, http://www.uniprot.org/uniprot/P49763) activate VEGFR1 homodimers, while VEGFC (http://www.guidetopharmacology.org/GRAC/LigandDisplayForward?ligandId=5087, http://www.uniprot.org/uniprot/P49767) and http://www.guidetopharmacology.org/GRAC/LigandDisplayForward?ligandId=5088 (https://www.genenames.org/data/gene‐symbol‐report/#!/hgnc_id/HGNC:3708, http://www.uniprot.org/uniprot/O43915) activate VEGFR2/3 heterodimers and VEGFR3 homodimers, and, following proteolysis, VEGFR2 homodimers.

**Table:** 

Nomenclature	http://www.guidetopharmacology.org/GRAC/ObjectDisplayForward?objectId=1812	http://www.guidetopharmacology.org/GRAC/ObjectDisplayForward?objectId=1813	http://www.guidetopharmacology.org/GRAC/ObjectDisplayForward?objectId=1814
Common abbreviation	VEGFR‐1	VEGFR‐2	VEGFR‐3
HGNC, UniProt	https://www.genenames.org/data/gene‐symbol‐report/#!/hgnc_id/HGNC:3763, http://www.uniprot.org/uniprot/P17948	https://www.genenames.org/data/gene‐symbol‐report/#!/hgnc_id/HGNC:6307, http://www.uniprot.org/uniprot/P35968	https://www.genenames.org/data/gene‐symbol‐report/#!/hgnc_id/HGNC:3767, http://www.uniprot.org/uniprot/P35916
EC number	http://www.genome.jp/dbget‐bin/www_bget?ec:2.7.10.1	http://www.genome.jp/dbget‐bin/www_bget?ec:2.7.10.1	http://www.genome.jp/dbget‐bin/www_bget?ec:2.7.10.1
Endogenous ligands	http://www.guidetopharmacology.org/GRAC/LigandDisplayForward?ligandId=5085 (http://www.guidetopharmacology.org/GRAC/LigandDisplayForward?ligandId=5085, http://www.uniprot.org/uniprot/P15692), http://www.guidetopharmacology.org/GRAC/LigandDisplayForward?ligandId=5086 (http://www.guidetopharmacology.org/GRAC/LigandDisplayForward?ligandId=5086, http://www.uniprot.org/uniprot/P49765)	http://www.guidetopharmacology.org/GRAC/LigandDisplayForward?ligandId=5085 (http://www.guidetopharmacology.org/GRAC/LigandDisplayForward?ligandId=5085, http://www.uniprot.org/uniprot/P15692), http://www.guidetopharmacology.org/GRAC/LigandDisplayForward?ligandId=5087 (http://www.guidetopharmacology.org/GRAC/LigandDisplayForward?ligandId=5087, http://www.uniprot.org/uniprot/P49767), http://www.guidetopharmacology.org/GRAC/LigandDisplayForward?ligandId=5089 (https://www.genenames.org/data/gene‐symbol‐report/#!/hgnc_id/HGNC:8801, http://www.uniprot.org/uniprot/Q9NRA1)	http://www.guidetopharmacology.org/GRAC/LigandDisplayForward?ligandId=5087 (http://www.guidetopharmacology.org/GRAC/LigandDisplayForward?ligandId=5087, http://www.uniprot.org/uniprot/P49767), http://www.guidetopharmacology.org/GRAC/LigandDisplayForward?ligandId=5088 (https://www.genenames.org/data/gene‐symbol‐report/#!/hgnc_id/HGNC:3708, http://www.uniprot.org/uniprot/O43915), http://www.guidetopharmacology.org/GRAC/LigandDisplayForward?ligandId=5089 (https://www.genenames.org/data/gene‐symbol‐report/#!/hgnc_id/HGNC:8801, http://www.uniprot.org/uniprot/Q9NRA1)
Inhibitors	http://www.guidetopharmacology.org/GRAC/LigandDisplayForward?ligandId=5712 (pIC_50_ 8.7) [http://www.ncbi.nlm.nih.gov/pubmed/16891463?dopt=AbstractPlus], http://www.guidetopharmacology.org/GRAC/LigandDisplayForward?ligandId=8189 (pIC_50_ 8.5) [http://www.ncbi.nlm.nih.gov/pubmed/22148921?dopt=AbstractPlus], http://www.guidetopharmacology.org/GRAC/LigandDisplayForward?ligandId=5056 (pIC_50_ 8.1) [http://www.ncbi.nlm.nih.gov/pubmed/10882357?dopt=AbstractPlus]	http://www.guidetopharmacology.org/GRAC/LigandDisplayForward?ligandId=5887 (pIC_50_ 10.5) [http://www.ncbi.nlm.nih.gov/pubmed/21926191?dopt=AbstractPlus], http://www.guidetopharmacology.org/GRAC/LigandDisplayForward?ligandId=5659 (pIC_50_ 9.6) [http://www.ncbi.nlm.nih.gov/pubmed/20869793?dopt=AbstractPlus], http://www.guidetopharmacology.org/GRAC/LigandDisplayForward?ligandId=5679 (pIC_50_ 8.2–9.1) [http://www.ncbi.nlm.nih.gov/pubmed/23098265?dopt=AbstractPlus], http://www.guidetopharmacology.org/GRAC/LigandDisplayForward?ligandId=5664 (p*K*_d_ 9) [http://www.ncbi.nlm.nih.gov/pubmed/22037378?dopt=AbstractPlus], http://www.guidetopharmacology.org/GRAC/LigandDisplayForward?ligandId=7944 (pIC_50_ 8.8) [http://www.ncbi.nlm.nih.gov/pubmed/17575237?dopt=AbstractPlus], http://www.guidetopharmacology.org/GRAC/LigandDisplayForward?ligandId=5660 (p*K*_d_ 8.6) [http://www.ncbi.nlm.nih.gov/pubmed/22037378?dopt=AbstractPlus], http://www.guidetopharmacology.org/GRAC/LigandDisplayForward?ligandId=7886 (pIC_50_ 8.3) [http://www.ncbi.nlm.nih.gov/pubmed/21028894?dopt=AbstractPlus], http://www.guidetopharmacology.org/GRAC/LigandDisplayForward?ligandId=5659 (p*K*_d_ 8.2) [http://www.ncbi.nlm.nih.gov/pubmed/22037378?dopt=AbstractPlus]	http://www.guidetopharmacology.org/GRAC/LigandDisplayForward?ligandId=7944 (pIC_50_ 8.1) [http://www.ncbi.nlm.nih.gov/pubmed/17575237?dopt=AbstractPlus], http://www.guidetopharmacology.org/GRAC/LigandDisplayForward?ligandId=5713 (pIC_50_ 8.1) [http://www.ncbi.nlm.nih.gov/pubmed/20570526?dopt=AbstractPlus], http://www.guidetopharmacology.org/GRAC/LigandDisplayForward?ligandId=5936 (pIC_50_ 7.9) [http://www.ncbi.nlm.nih.gov/pubmed/18559524?dopt=AbstractPlus]
Sub/family‐selective inhibitors	http://www.guidetopharmacology.org/GRAC/LigandDisplayForward?ligandId=5698 (pIC_50_ 8) [http://www.ncbi.nlm.nih.gov/pubmed/18620382?dopt=AbstractPlus]	http://www.guidetopharmacology.org/GRAC/LigandDisplayForward?ligandId=5698 (p*K*_d_ 7.8) [http://www.ncbi.nlm.nih.gov/pubmed/22037378?dopt=AbstractPlus], http://www.guidetopharmacology.org/GRAC/LigandDisplayForward?ligandId=5698 (pIC_50_ 7.5) [http://www.ncbi.nlm.nih.gov/pubmed/18620382?dopt=AbstractPlus]	http://www.guidetopharmacology.org/GRAC/LigandDisplayForward?ligandId=5698 (pIC_50_ 7.3) [http://www.ncbi.nlm.nih.gov/pubmed/18620382?dopt=AbstractPlus]
Antibodies	–	http://www.guidetopharmacology.org/GRAC/LigandDisplayForward?ligandId=7390 (Antagonist) (pIC_50_ 9) [http://www.ncbi.nlm.nih.gov/pubmed/12917408?dopt=AbstractPlus]	–

### Comments

The VEGFR, as well as VEGF ligands, have been targeted by antibodies and tyrosine kinase inhibitors. DMH4 [http://www.ncbi.nlm.nih.gov/pubmed/12443771?dopt=AbstractPlus], Ki8751 [http://www.ncbi.nlm.nih.gov/pubmed/15743179?dopt=AbstractPlus] and ZM323881, a novel inhibitor of vascular endothelial growth factor‐receptor‐2 tyrosine kinase activity [http://www.ncbi.nlm.nih.gov/pubmed/12483548?dopt=AbstractPlus] are described as VEGFR2‐selective tyrosine kinase inhibitors. Bevacizumab is a monoclonal antibody directed against VEGF‐A, used clinically for the treatment of certain metastatic cancers; an antibody fragment has been used for wet age‐related macular degeneration.

## http://www.guidetopharmacology.org/GRAC/FamilyDisplayForward?familyId=323

### Overview

Fibroblast growth factor (FGF) family receptors act as homo‐ and heterodimers, and are characterized by Ig‐like loops in the extracellular domain, in which disulphide bridges may form across protein partners to allow the formation of covalent dimers which may be constitutively active. FGF receptors have been implicated in achondroplasia, angiogenesis and numerous congenital disorders. At least 22 members of the FGF gene family have been identified in the human genome [11]. Within this group, subfamilies of FGF may be divided into canonical, intracellular and hormone‐like FGFs. FGF1‐FGF10 have been identified to act through FGF receptors, while FGF11‐14 appear to signal through intracellular targets. Other family members are less well characterized [http://www.ncbi.nlm.nih.gov/pubmed/21711248?dopt=AbstractPlus].

**Table:** 

Nomenclature	http://www.guidetopharmacology.org/GRAC/ObjectDisplayForward?objectId=1808	http://www.guidetopharmacology.org/GRAC/ObjectDisplayForward?objectId=1809	http://www.guidetopharmacology.org/GRAC/ObjectDisplayForward?objectId=1810	http://www.guidetopharmacology.org/GRAC/ObjectDisplayForward?objectId=1811
Common abbreviation	FGFR1	FGFR2	FGFR3	FGFR4
HGNC, UniProt	https://www.genenames.org/data/gene‐symbol‐report/#!/hgnc_id/HGNC:3688, http://www.uniprot.org/uniprot/P11362	https://www.genenames.org/data/gene‐symbol‐report/#!/hgnc_id/HGNC:3689, http://www.uniprot.org/uniprot/P21802	https://www.genenames.org/data/gene‐symbol‐report/#!/hgnc_id/HGNC:3690, http://www.uniprot.org/uniprot/P22607	https://www.genenames.org/data/gene‐symbol‐report/#!/hgnc_id/HGNC:3691, http://www.uniprot.org/uniprot/P22455
EC number	http://www.genome.jp/dbget‐bin/www_bget?ec:2.7.10.1	http://www.genome.jp/dbget‐bin/www_bget?ec:2.7.10.1	http://www.genome.jp/dbget‐bin/www_bget?ec:2.7.10.1	http://www.genome.jp/dbget‐bin/www_bget?ec:2.7.10.1
Endogenous ligands	http://www.guidetopharmacology.org/GRAC/LigandDisplayForward?ligandId=4923 (https://www.genenames.org/data/gene‐symbol‐report/#!/hgnc_id/HGNC:3665, http://www.uniprot.org/uniprot/P05230), http://www.guidetopharmacology.org/GRAC/LigandDisplayForward?ligandId=4924 (https://www.genenames.org/data/gene‐symbol‐report/#!/hgnc_id/HGNC:3676, http://www.uniprot.org/uniprot/P09038), http://www.guidetopharmacology.org/GRAC/LigandDisplayForward?ligandId=4925 (https://www.genenames.org/data/gene‐symbol‐report/#!/hgnc_id/HGNC:3682, http://www.uniprot.org/uniprot/P08620) > http://www.guidetopharmacology.org/GRAC/LigandDisplayForward?ligandId=4926 (https://www.genenames.org/data/gene‐symbol‐report/#!/hgnc_id/HGNC:3683, http://www.uniprot.org/uniprot/P12034), http://www.guidetopharmacology.org/GRAC/LigandDisplayForward?ligandId=4927 (https://www.genenames.org/data/gene‐symbol‐report/#!/hgnc_id/HGNC:3684, http://www.uniprot.org/uniprot/P10767) [http://www.ncbi.nlm.nih.gov/pubmed/8663044?dopt=AbstractPlus]	http://www.guidetopharmacology.org/GRAC/LigandDisplayForward?ligandId=4923 (https://www.genenames.org/data/gene‐symbol‐report/#!/hgnc_id/HGNC:3665, http://www.uniprot.org/uniprot/P05230) > http://www.guidetopharmacology.org/GRAC/LigandDisplayForward?ligandId=4925 (https://www.genenames.org/data/gene‐symbol‐report/#!/hgnc_id/HGNC:3682, http://www.uniprot.org/uniprot/P08620), http://www.guidetopharmacology.org/GRAC/LigandDisplayForward?ligandId=4928 (https://www.genenames.org/data/gene‐symbol‐report/#!/hgnc_id/HGNC:3685, http://www.uniprot.org/uniprot/P21781), http://www.guidetopharmacology.org/GRAC/LigandDisplayForward?ligandId=4930 (https://www.genenames.org/data/gene‐symbol‐report/#!/hgnc_id/HGNC:3687, http://www.uniprot.org/uniprot/P31371) > http://www.guidetopharmacology.org/GRAC/LigandDisplayForward?ligandId=4924 (https://www.genenames.org/data/gene‐symbol‐report/#!/hgnc_id/HGNC:3676, http://www.uniprot.org/uniprot/P09038), http://www.guidetopharmacology.org/GRAC/LigandDisplayForward?ligandId=4927 (https://www.genenames.org/data/gene‐symbol‐report/#!/hgnc_id/HGNC:3684, http://www.uniprot.org/uniprot/P10767) [http://www.ncbi.nlm.nih.gov/pubmed/8663044?dopt=AbstractPlus] http://www.guidetopharmacology.org/GRAC/LigandDisplayForward?ligandId=9641 (https://www.genenames.org/data/gene‐symbol‐report/#!/hgnc_id/HGNC:3666, http://www.uniprot.org/uniprot/O15520) [http://www.ncbi.nlm.nih.gov/pubmed/12591959?dopt=AbstractPlus]	http://www.guidetopharmacology.org/GRAC/LigandDisplayForward?ligandId=4923 (https://www.genenames.org/data/gene‐symbol‐report/#!/hgnc_id/HGNC:3665, http://www.uniprot.org/uniprot/P05230), http://www.guidetopharmacology.org/GRAC/LigandDisplayForward?ligandId=4924 (https://www.genenames.org/data/gene‐symbol‐report/#!/hgnc_id/HGNC:3676, http://www.uniprot.org/uniprot/P09038), http://www.guidetopharmacology.org/GRAC/LigandDisplayForward?ligandId=4930 (https://www.genenames.org/data/gene‐symbol‐report/#!/hgnc_id/HGNC:3687, http://www.uniprot.org/uniprot/P31371) > http://www.guidetopharmacology.org/GRAC/LigandDisplayForward?ligandId=4925 (https://www.genenames.org/data/gene‐symbol‐report/#!/hgnc_id/HGNC:3682, http://www.uniprot.org/uniprot/P08620), http://www.guidetopharmacology.org/GRAC/LigandDisplayForward?ligandId=4929 (https://www.genenames.org/data/gene‐symbol‐report/#!/hgnc_id/HGNC:3686, http://www.uniprot.org/uniprot/P55075) [http://www.ncbi.nlm.nih.gov/pubmed/8663044?dopt=AbstractPlus] http://www.guidetopharmacology.org/GRAC/LigandDisplayForward?ligandId=9629 (https://www.genenames.org/data/gene‐symbol‐report/#!/hgnc_id/HGNC:3681, http://www.uniprot.org/uniprot/P11487)	http://www.guidetopharmacology.org/GRAC/LigandDisplayForward?ligandId=4923 (https://www.genenames.org/data/gene‐symbol‐report/#!/hgnc_id/HGNC:3665, http://www.uniprot.org/uniprot/P05230), http://www.guidetopharmacology.org/GRAC/LigandDisplayForward?ligandId=4924 (https://www.genenames.org/data/gene‐symbol‐report/#!/hgnc_id/HGNC:3676, http://www.uniprot.org/uniprot/P09038), http://www.guidetopharmacology.org/GRAC/LigandDisplayForward?ligandId=4925 (https://www.genenames.org/data/gene‐symbol‐report/#!/hgnc_id/HGNC:3682, http://www.uniprot.org/uniprot/P08620), http://www.guidetopharmacology.org/GRAC/LigandDisplayForward?ligandId=4930 (https://www.genenames.org/data/gene‐symbol‐report/#!/hgnc_id/HGNC:3687, http://www.uniprot.org/uniprot/P31371) > http://www.guidetopharmacology.org/GRAC/LigandDisplayForward?ligandId=4927 (https://www.genenames.org/data/gene‐symbol‐report/#!/hgnc_id/HGNC:3684, http://www.uniprot.org/uniprot/P10767), http://www.guidetopharmacology.org/GRAC/LigandDisplayForward?ligandId=4929 (https://www.genenames.org/data/gene‐symbol‐report/#!/hgnc_id/HGNC:3686, http://www.uniprot.org/uniprot/P55075) [http://www.ncbi.nlm.nih.gov/pubmed/8663044?dopt=AbstractPlus]
Sub/family‐selective inhibitors	http://www.guidetopharmacology.org/GRAC/LigandDisplayForward?ligandId=8104 (pIC_50_ 8.6) [http://www.ncbi.nlm.nih.gov/pubmed/21900693?dopt=AbstractPlus]	http://www.guidetopharmacology.org/GRAC/LigandDisplayForward?ligandId=8104 (pIC_50_ 8.6) [http://www.ncbi.nlm.nih.gov/pubmed/21900693?dopt=AbstractPlus]	http://www.guidetopharmacology.org/GRAC/LigandDisplayForward?ligandId=8104 (pIC_50_ 8.2) [http://www.ncbi.nlm.nih.gov/pubmed/21900693?dopt=AbstractPlus]	http://www.guidetopharmacology.org/GRAC/LigandDisplayForward?ligandId=8104 (pIC_50_ 8.2) [http://www.ncbi.nlm.nih.gov/pubmed/21900693?dopt=AbstractPlus]
Selective inhibitors	–	–	–	http://www.guidetopharmacology.org/GRAC/LigandDisplayForward?ligandId=8862 (Irreversible inhibition) (pIC_50_ 8.5) [http://www.ncbi.nlm.nih.gov/pubmed/25776529?dopt=AbstractPlus]
Agonists	–	http://www.guidetopharmacology.org/GRAC/LigandDisplayForward?ligandId=6954	–	–

### Comments

Splice variation of the receptors can influence agonist responses. https://www.genenames.org/data/gene‐symbol‐report/#!/hgnc_id/HGNC:3693 (http://www.uniprot.org/uniprot/Q8N441) is a truncated kinase‐null analogue. Various antibodies and tyrosine kinase inhibitors have been developed against FGF receptors [http://www.ncbi.nlm.nih.gov/pubmed/22884522?dopt=AbstractPlus, http://www.ncbi.nlm.nih.gov/pubmed/20338520?dopt=AbstractPlus]. PD161570 is an FGFR tyrosine kinase inhibitor [http://www.ncbi.nlm.nih.gov/pubmed/9488112?dopt=AbstractPlus], while http://www.guidetopharmacology.org/GRAC/LigandDisplayForward?ligandId=5037 has been described to inhibit FGFR1 and FGFR3 [http://www.ncbi.nlm.nih.gov/pubmed/10987832?dopt=AbstractPlus].

## http://www.guidetopharmacology.org/GRAC/FamilyDisplayForward?familyId=792

### Overview

The PTK7 receptor is associated with polarization of epithelial cells and the development of neural structures. Sequence analysis suggests that the gene product is catalytically inactive as a protein kinase, although there is evidence for a role in Wnt signalling [http://www.ncbi.nlm.nih.gov/pubmed/21132015?dopt=AbstractPlus].

**Table:** 

Nomenclature	http://www.guidetopharmacology.org/GRAC/ObjectDisplayForward?objectId=1848
Common abbreviation	CCK4
HGNC, UniProt	https://www.genenames.org/data/gene‐symbol‐report/#!/hgnc_id/HGNC:9618, http://www.uniprot.org/uniprot/Q13308
EC number	http://www.genome.jp/dbget‐bin/www_bget?ec:2.7.10.1

### Comments

Thus far, no selective PTK7 inhibitors have been described.

## http://www.guidetopharmacology.org/GRAC/FamilyDisplayForward?familyId=326

### Overview

The neurotrophin receptor family of RTKs include trkA, trkB and trkC (tropomyosin‐related kinase) receptors, which respond to NGF, BDNF and neurotrophin‐3, respectively. They are associated primarily with proliferative and migration effects in neural systems. Various isoforms of neurotrophin receptors exist, including truncated forms of trkB and trkC, which lack catalytic domains. p75 (http://www.guidetopharmacology.org/GRAC/FamilyDisplayForward?familyId=334#show_object_1888, also known as nerve growth factor receptor), which has homologies with http://www.guidetopharmacology.org/GRAC/FamilyDisplayForward?familyId=334, lacks a tyrosine kinase domain, but can signal via ceramide release and nuclear factor θB (NF‐θB) activation. Both trkA and trkB contain two leucine‐rich regions and can exist in monomeric or dimeric forms.

**Table:** 

Nomenclature	http://www.guidetopharmacology.org/GRAC/ObjectDisplayForward?objectId=1817	http://www.guidetopharmacology.org/GRAC/ObjectDisplayForward?objectId=1818	http://www.guidetopharmacology.org/GRAC/ObjectDisplayForward?objectId=1819
Common abbreviation	trkA	trkB	trkC
HGNC, UniProt	https://www.genenames.org/data/gene‐symbol‐report/#!/hgnc_id/HGNC:8031, http://www.uniprot.org/uniprot/P04629	https://www.genenames.org/data/gene‐symbol‐report/#!/hgnc_id/HGNC:8032, http://www.uniprot.org/uniprot/Q16620	https://www.genenames.org/data/gene‐symbol‐report/#!/hgnc_id/HGNC:8033, http://www.uniprot.org/uniprot/Q16288
EC number	http://www.genome.jp/dbget‐bin/www_bget?ec:2.7.10.1	http://www.genome.jp/dbget‐bin/www_bget?ec:2.7.10.1	http://www.genome.jp/dbget‐bin/www_bget?ec:2.7.10.1
Endogenous ligands	http://www.guidetopharmacology.org/GRAC/LigandDisplayForward?ligandId=5026 (http://www.guidetopharmacology.org/GRAC/LigandDisplayForward?ligandId=5026, http://www.uniprot.org/uniprot/P01138) > http://www.guidetopharmacology.org/GRAC/LigandDisplayForward?ligandId=5033 (https://www.genenames.org/data/gene‐symbol‐report/#!/hgnc_id/HGNC:8023, http://www.uniprot.org/uniprot/P20783)	http://www.guidetopharmacology.org/GRAC/LigandDisplayForward?ligandId=4872 (http://www.guidetopharmacology.org/GRAC/LigandDisplayForward?ligandId=4872, http://www.uniprot.org/uniprot/P23560), http://www.guidetopharmacology.org/GRAC/LigandDisplayForward?ligandId=5034 (https://www.genenames.org/data/gene‐symbol‐report/#!/hgnc_id/HGNC:8024, http://www.uniprot.org/uniprot/P34130) > http://www.guidetopharmacology.org/GRAC/LigandDisplayForward?ligandId=5033 (https://www.genenames.org/data/gene‐symbol‐report/#!/hgnc_id/HGNC:8023, http://www.uniprot.org/uniprot/P20783)	http://www.guidetopharmacology.org/GRAC/LigandDisplayForward?ligandId=5033 (https://www.genenames.org/data/gene‐symbol‐report/#!/hgnc_id/HGNC:8023, http://www.uniprot.org/uniprot/P20783)
Inhibitors	http://www.guidetopharmacology.org/GRAC/LigandDisplayForward?ligandId=10314 (pIC_50_ >9.3) [165], http://www.guidetopharmacology.org/GRAC/LigandDisplayForward?ligandId=8138 (pIC_50_ 8.9) [http://www.ncbi.nlm.nih.gov/pubmed/24900538?dopt=AbstractPlus], http://www.guidetopharmacology.org/GRAC/LigandDisplayForward?ligandId=7938 (pIC_50_ 7.3) [http://www.ncbi.nlm.nih.gov/pubmed/19603809?dopt=AbstractPlus]	–	–
Sub/family‐selective inhibitors	http://www.guidetopharmacology.org/GRAC/LigandDisplayForward?ligandId=7700 (pIC_50_ >8.3) [6], http://www.guidetopharmacology.org/GRAC/LigandDisplayForward?ligandId=8071 (pIC_50_ 8) [http://www.ncbi.nlm.nih.gov/pubmed/24900443?dopt=AbstractPlus]	http://www.guidetopharmacology.org/GRAC/LigandDisplayForward?ligandId=7700 (pIC_50_ >8.3) [6], http://www.guidetopharmacology.org/GRAC/LigandDisplayForward?ligandId=8071 (pIC_50_ 8.1) [http://www.ncbi.nlm.nih.gov/pubmed/24900443?dopt=AbstractPlus]	http://www.guidetopharmacology.org/GRAC/LigandDisplayForward?ligandId=7700 (pIC_50_ >8.3) [6], http://www.guidetopharmacology.org/GRAC/LigandDisplayForward?ligandId=8071 (pIC_50_ 8.1) [http://www.ncbi.nlm.nih.gov/pubmed/24900443?dopt=AbstractPlus]

### Comments

http://www.guidetopharmacology.org/GRAC/LigandDisplayForward?ligandId=4849 and http://www.guidetopharmacology.org/GRAC/LigandDisplayForward?ligandId=4846 have been used to label the trkA and trkB receptor, respectively. p75 influences the binding of http://www.guidetopharmacology.org/GRAC/LigandDisplayForward?ligandId=5026 (http://www.guidetopharmacology.org/GRAC/LigandDisplayForward?ligandId=5026, http://www.uniprot.org/uniprot/P01138) and http://www.guidetopharmacology.org/GRAC/LigandDisplayForward?ligandId=5033 (https://www.genenames.org/data/gene‐symbol‐report/#!/hgnc_id/HGNC:8023, http://www.uniprot.org/uniprot/P20783) to trkA. The ligand selectivity of p75 appears to be dependent on the cell type; for example, in sympathetic neurones, it binds http://www.guidetopharmacology.org/GRAC/LigandDisplayForward?ligandId=5033 (https://www.genenames.org/data/gene‐symbol‐report/#!/hgnc_id/HGNC:8023, http://www.uniprot.org/uniprot/P20783) with comparable affinity to trkC [http://www.ncbi.nlm.nih.gov/pubmed/9204912?dopt=AbstractPlus].

Small molecule agonists of trkB have been described, including LM22A4 [http://www.ncbi.nlm.nih.gov/pubmed/20407211?dopt=AbstractPlus], while ANA12 has been described as a noncompetitive antagonist of BDNF binding to trkB [http://www.ncbi.nlm.nih.gov/pubmed/21505263?dopt=AbstractPlus]. GNF5837 is a family‐selective tyrosine kinase inhibitor [http://www.ncbi.nlm.nih.gov/pubmed/24900443?dopt=AbstractPlus], while the tyrosine kinase activity of the trkA receptor can be inhibited by http://www.guidetopharmacology.org/GRAC/LigandDisplayForward?ligandId=4946 (pIC_50_= 8.7, [http://www.ncbi.nlm.nih.gov/pubmed/15013000?dopt=AbstractPlus]) and tyrphostin http://www.guidetopharmacology.org/GRAC/LigandDisplayForward?ligandId=4863 [http://www.ncbi.nlm.nih.gov/pubmed/7683492?dopt=AbstractPlus].

## http://www.guidetopharmacology.org/GRAC/FamilyDisplayForward?familyId=332

### Overview

Members of the ROR family appear to be activated by ligands complexing with other cell‐surface proteins. Thus, ROR1 and ROR2 appear to be activated by http://www.guidetopharmacology.org/GRAC/LigandDisplayForward?ligandId=3548 (https://www.genenames.org/data/gene‐symbol‐report/#!/hgnc_id/HGNC:12784, http://www.uniprot.org/uniprot/P41221) binding to a http://www.guidetopharmacology.org/GRAC/FamilyDisplayForward?familyId=25 thereby forming a cell‐surface multiprotein complex [http://www.ncbi.nlm.nih.gov/pubmed/21078818?dopt=AbstractPlus].

**Table:** 

Nomenclature	http://www.guidetopharmacology.org/GRAC/ObjectDisplayForward?objectId=1845	http://www.guidetopharmacology.org/GRAC/ObjectDisplayForward?objectId=1846
Common abbreviation	ROR1	ROR2
HGNC, UniProt	https://www.genenames.org/data/gene‐symbol‐report/#!/hgnc_id/HGNC:10256, http://www.uniprot.org/uniprot/Q01973	https://www.genenames.org/data/gene‐symbol‐report/#!/hgnc_id/HGNC:10257, http://www.uniprot.org/uniprot/Q01974
EC number	http://www.genome.jp/dbget‐bin/www_bget?ec:2.7.10.1	http://www.genome.jp/dbget‐bin/www_bget?ec:2.7.10.1

## http://www.guidetopharmacology.org/GRAC/FamilyDisplayForward?familyId=793

### Overview

The muscle‐specific kinase MuSK is associated with the formation and organisation of the neuromuscular junction from the skeletal muscle side. http://www.guidetopharmacology.org/GRAC/LigandDisplayForward?ligandId=5319 (https://www.genenames.org/data/gene‐symbol‐report/#!/hgnc_id/HGNC:329, http://www.uniprot.org/uniprot/O00468) forms a complex with http://www.guidetopharmacology.org/GRAC/LigandDisplayForward?ligandId=5320 (https://www.genenames.org/data/gene‐symbol‐report/#!/hgnc_id/HGNC:6696, http://www.uniprot.org/uniprot/O75096) to activate MuSK [http://www.ncbi.nlm.nih.gov/pubmed/18848351?dopt=AbstractPlus].

**Table:** 

Nomenclature	http://www.guidetopharmacology.org/GRAC/ObjectDisplayForward?objectId=1847
Common abbreviation	MuSK
HGNC, UniProt	https://www.genenames.org/data/gene‐symbol‐report/#!/hgnc_id/HGNC:7525, http://www.uniprot.org/uniprot/O15146
EC number	http://www.genome.jp/dbget‐bin/www_bget?ec:2.7.10.1

### Comments

Thus far, no selective MuSK inhibitors have been described.

## http://www.guidetopharmacology.org/GRAC/FamilyDisplayForward?familyId=325

### Overview

HGF receptors regulatematuration of the liver in the embryo, as well as having roles in the adult, for example, in the innate immune system. HGF is synthesized as a single gene product, which is post‐translationally processed to yield a heterodimer linked by a disulphide bridge. The maturation of HGF is enhanced by a serine protease, HGF activating complex, and inhibited by http://www.guidetopharmacology.org/GRAC/LigandDisplayForward?ligandId=5315 (https://www.genenames.org/data/gene‐symbol‐report/#!/hgnc_id/HGNC:11246, http://www.uniprot.org/uniprot/O43278), a serine protease inhibitor. MST1, the ligand of RON, is two disulphide‐linked peptide chains generated by proteolysis of a single gene product.

**Table:** 

Nomenclature	http://www.guidetopharmacology.org/GRAC/ObjectDisplayForward?objectId=1815	http://www.guidetopharmacology.org/GRAC/ObjectDisplayForward?objectId=1816
Common abbreviation	MET	Ron
HGNC, UniProt	https://www.genenames.org/data/gene‐symbol‐report/#!/hgnc_id/HGNC:7029, http://www.uniprot.org/uniprot/P08581	https://www.genenames.org/data/gene‐symbol‐report/#!/hgnc_id/HGNC:7381, http://www.uniprot.org/uniprot/Q04912
EC number	http://www.genome.jp/dbget‐bin/www_bget?ec:2.7.10.1	http://www.genome.jp/dbget‐bin/www_bget?ec:2.7.10.1
Endogenous ligands	http://www.guidetopharmacology.org/GRAC/LigandDisplayForward?ligandId=4949 (https://www.genenames.org/data/gene‐symbol‐report/#!/hgnc_id/HGNC:4893, http://www.uniprot.org/uniprot/P14210)	http://www.guidetopharmacology.org/GRAC/LigandDisplayForward?ligandId=5023 (https://www.genenames.org/data/gene‐symbol‐report/#!/hgnc_id/HGNC:7380, http://www.uniprot.org/uniprot/P09603)
Inhibitors	http://www.guidetopharmacology.org/GRAC/LigandDisplayForward?ligandId=7904 (pIC_50_ 9.9) [http://www.ncbi.nlm.nih.gov/pubmed/21918175?dopt=AbstractPlus], http://www.guidetopharmacology.org/GRAC/LigandDisplayForward?ligandId=5709 (p*K*_d_ 9.7) [http://www.ncbi.nlm.nih.gov/pubmed/22037378?dopt=AbstractPlus], http://www.guidetopharmacology.org/GRAC/LigandDisplayForward?ligandId=5887 (pIC_50_ 8.9) [http://www.ncbi.nlm.nih.gov/pubmed/21926191?dopt=AbstractPlus]	http://www.guidetopharmacology.org/GRAC/LigandDisplayForward?ligandId=7953 (pIC_50_ 8.7) [http://www.ncbi.nlm.nih.gov/pubmed/19260711?dopt=AbstractPlus]
Selective inhibitors	http://www.guidetopharmacology.org/GRAC/LigandDisplayForward?ligandId=5709 (pIC_50_ 8.4) [http://www.ncbi.nlm.nih.gov/pubmed/19934279?dopt=AbstractPlus]	–

### Comments

PF04217903 is a selective Met tyrosine kinase inhibitor [http://www.ncbi.nlm.nih.gov/pubmed/22924734?dopt=AbstractPlus]. http://www.guidetopharmacology.org/GRAC/LigandDisplayForward?ligandId=5057 is an inhibitor of the HGF receptor [http://www.ncbi.nlm.nih.gov/pubmed/14500382?dopt=AbstractPlus], with the possibility of further targets [http://www.ncbi.nlm.nih.gov/pubmed/17595299?dopt=AbstractPlus].

## http://www.guidetopharmacology.org/GRAC/FamilyDisplayForward?familyId=328

### Overview

Members of this RTK family represented a novel structural motif, when sequenced. The ligands for this family, http://www.guidetopharmacology.org/GRAC/LigandDisplayForward?ligandId=4935 (https://www.genenames.org/data/gene‐symbol‐report/#!/hgnc_id/HGNC:4168, http://www.uniprot.org/uniprot/Q14393) and http://www.guidetopharmacology.org/GRAC/LigandDisplayForward?ligandId=5050 (https://www.genenames.org/data/gene‐symbol‐report/#!/hgnc_id/HGNC:9456, http://www.uniprot.org/uniprot/P07225), are secreted plasma proteins which undergo vitamin K‐dependent post‐translational modifications generating carboxyglutamate‐rich domains which are able to bind to negatively‐charged surfaces of apoptotic cells.

**Table:** 

Nomenclature	http://www.guidetopharmacology.org/GRAC/ObjectDisplayForward?objectId=1835	http://www.guidetopharmacology.org/GRAC/ObjectDisplayForward?objectId=1836	http://www.guidetopharmacology.org/GRAC/ObjectDisplayForward?objectId=1837
Common abbreviation	Axl	Tyro3	Mer
HGNC, UniProt	https://www.genenames.org/data/gene‐symbol‐report/#!/hgnc_id/HGNC:905, http://www.uniprot.org/uniprot/P30530	https://www.genenames.org/data/gene‐symbol‐report/#!/hgnc_id/HGNC:12446, http://www.uniprot.org/uniprot/Q06418	https://www.genenames.org/data/gene‐symbol‐report/#!/hgnc_id/HGNC:7027, http://www.uniprot.org/uniprot/Q12866
EC number	http://www.genome.jp/dbget‐bin/www_bget?ec:2.7.10.1	http://www.genome.jp/dbget‐bin/www_bget?ec:2.7.10.1	http://www.genome.jp/dbget‐bin/www_bget?ec:2.7.10.1
Endogenous ligands	http://www.guidetopharmacology.org/GRAC/LigandDisplayForward?ligandId=4935 (https://www.genenames.org/data/gene‐symbol‐report/#!/hgnc_id/HGNC:4168, http://www.uniprot.org/uniprot/Q14393) [http://www.ncbi.nlm.nih.gov/pubmed/8939948?dopt=AbstractPlus], http://www.guidetopharmacology.org/GRAC/LigandDisplayForward?ligandId=5050 (https://www.genenames.org/data/gene‐symbol‐report/#!/hgnc_id/HGNC:9456, http://www.uniprot.org/uniprot/P07225) [http://www.ncbi.nlm.nih.gov/pubmed/7867073?dopt=AbstractPlus]	http://www.guidetopharmacology.org/GRAC/LigandDisplayForward?ligandId=4935 (https://www.genenames.org/data/gene‐symbol‐report/#!/hgnc_id/HGNC:4168, http://www.uniprot.org/uniprot/Q14393) [http://www.ncbi.nlm.nih.gov/pubmed/8939948?dopt=AbstractPlus], http://www.guidetopharmacology.org/GRAC/LigandDisplayForward?ligandId=5050 (https://www.genenames.org/data/gene‐symbol‐report/#!/hgnc_id/HGNC:9456, http://www.uniprot.org/uniprot/P07225) [http://www.ncbi.nlm.nih.gov/pubmed/7867073?dopt=AbstractPlus]	http://www.guidetopharmacology.org/GRAC/LigandDisplayForward?ligandId=4935 (https://www.genenames.org/data/gene‐symbol‐report/#!/hgnc_id/HGNC:4168, http://www.uniprot.org/uniprot/Q14393) [http://www.ncbi.nlm.nih.gov/pubmed/8939948?dopt=AbstractPlus]

### Comments

AXL tyrosine kinase inhibitors have been described [http://www.ncbi.nlm.nih.gov/pubmed/22247788?dopt=AbstractPlus].

## http://www.guidetopharmacology.org/GRAC/FamilyDisplayForward?familyId=330

### Overview

The TIE family were initially associated with formation of blood vessels. Endogenous ligands are http://www.guidetopharmacology.org/GRAC/LigandDisplayForward?ligandId=4867 (https://www.genenames.org/data/gene‐symbol‐report/#!/hgnc_id/HGNC:484, http://www.uniprot.org/uniprot/Q15389), http://www.guidetopharmacology.org/GRAC/LigandDisplayForward?ligandId=5316 (https://www.genenames.org/data/gene‐symbol‐report/#!/hgnc_id/HGNC:485, http://www.uniprot.org/uniprot/O15123), and http://www.guidetopharmacology.org/GRAC/LigandDisplayForward?ligandId=4868 (https://www.genenames.org/data/gene‐symbol‐report/#!/hgnc_id/HGNC:487, http://www.uniprot.org/uniprot/Q9Y264). http://www.guidetopharmacology.org/GRAC/LigandDisplayForward?ligandId=5316 (https://www.genenames.org/data/gene‐symbol‐report/#!/hgnc_id/HGNC:485, http://www.uniprot.org/uniprot/O15123) appears to act as an endogenous antagonist of angiopoietin‐1 function.

**Table:** 

Nomenclature	http://www.guidetopharmacology.org/GRAC/ObjectDisplayForward?objectId=1841	http://www.guidetopharmacology.org/GRAC/ObjectDisplayForward?objectId=1842
Common abbreviation	TIE1	TIE2
HGNC, UniProt	https://www.genenames.org/data/gene‐symbol‐report/#!/hgnc_id/HGNC:11809, http://www.uniprot.org/uniprot/P35590	https://www.genenames.org/data/gene‐symbol‐report/#!/hgnc_id/HGNC:11724, http://www.uniprot.org/uniprot/Q02763
EC number	http://www.genome.jp/dbget‐bin/www_bget?ec:2.7.10.1	http://www.genome.jp/dbget‐bin/www_bget?ec:2.7.10.1
Endogenous ligands	–	http://www.guidetopharmacology.org/GRAC/LigandDisplayForward?ligandId=4867 (https://www.genenames.org/data/gene‐symbol‐report/#!/hgnc_id/HGNC:484, http://www.uniprot.org/uniprot/Q15389), http://www.guidetopharmacology.org/GRAC/LigandDisplayForward?ligandId=4868 (https://www.genenames.org/data/gene‐symbol‐report/#!/hgnc_id/HGNC:487, http://www.uniprot.org/uniprot/Q9Y264)

## http://www.guidetopharmacology.org/GRAC/FamilyDisplayForward?familyId=327

### Overview

Ephrin receptors are a family of 15 RTKs (the largest family of RTKs) with two identified subfamilies (EphA and EphB), which have a role in the regulation of neuronal development, cell migration, patterning and angiogenesis. Their ligands are membrane‐associated proteins, thought to be glycosylphosphatidylinositol‐linked for EphA (http://www.guidetopharmacology.org/GRAC/LigandDisplayForward?ligandId=4908 (https://www.genenames.org/data/gene‐symbol‐report/#!/hgnc_id/HGNC:3221, http://www.uniprot.org/uniprot/P20827), http://www.guidetopharmacology.org/GRAC/LigandDisplayForward?ligandId=4909 (https://www.genenames.org/data/gene‐symbol‐report/#!/hgnc_id/HGNC:3222, http://www.uniprot.org/uniprot/O43921), http://www.guidetopharmacology.org/GRAC/LigandDisplayForward?ligandId=4910 (https://www.genenames.org/data/gene‐symbol‐report/#!/hgnc_id/HGNC:3223, http://www.uniprot.org/uniprot/P52797), http://www.guidetopharmacology.org/GRAC/LigandDisplayForward?ligandId=4911 (https://www.genenames.org/data/gene‐symbol‐report/#!/hgnc_id/HGNC:3224, http://www.uniprot.org/uniprot/P52798) and http://www.guidetopharmacology.org/GRAC/LigandDisplayForward?ligandId=4912 (https://www.genenames.org/data/gene‐symbol‐report/#!/hgnc_id/HGNC:3225, http://www.uniprot.org/uniprot/P52803)) and 1TM proteins for Ephrin B (http://www.ensembl.org/Homo_sapiens/Gene/Family/Genes?family=ENSFM00250000002014: http://www.guidetopharmacology.org/GRAC/LigandDisplayForward?ligandId=4913 (https://www.genenames.org/data/gene‐symbol‐report/#!/hgnc_id/HGNC:3226, http://www.uniprot.org/uniprot/P98172), http://www.guidetopharmacology.org/GRAC/LigandDisplayForward?ligandId=4914 (https://www.genenames.org/data/gene‐symbol‐report/#!/hgnc_id/HGNC:3227, http://www.uniprot.org/uniprot/P52799) and http://www.guidetopharmacology.org/GRAC/LigandDisplayForward?ligandId=4915 (https://www.genenames.org/data/gene‐symbol‐report/#!/hgnc_id/HGNC:3228, http://www.uniprot.org/uniprot/Q15768)), although the relationship between ligands and receptors has been incompletely defined.

**Table:** 

Nomenclature	http://www.guidetopharmacology.org/GRAC/ObjectDisplayForward?objectId=1821	http://www.guidetopharmacology.org/GRAC/ObjectDisplayForward?objectId=1822	http://www.guidetopharmacology.org/GRAC/ObjectDisplayForward?objectId=1823	http://www.guidetopharmacology.org/GRAC/ObjectDisplayForward?objectId=1824	http://www.guidetopharmacology.org/GRAC/ObjectDisplayForward?objectId=1825	http://www.guidetopharmacology.org/GRAC/ObjectDisplayForward?objectId=1826	http://www.guidetopharmacology.org/GRAC/ObjectDisplayForward?objectId=1827
Common abbreviation	EphA1	EphA2	EphA3	EphA4	EphA5	EphA6	EphA7
HGNC, UniProt	https://www.genenames.org/data/gene‐symbol‐report/#!/hgnc_id/HGNC:3385, http://www.uniprot.org/uniprot/P21709	https://www.genenames.org/data/gene‐symbol‐report/#!/hgnc_id/HGNC:3386, http://www.uniprot.org/uniprot/P29317	https://www.genenames.org/data/gene‐symbol‐report/#!/hgnc_id/HGNC:3387, http://www.uniprot.org/uniprot/P29320	https://www.genenames.org/data/gene‐symbol‐report/#!/hgnc_id/HGNC:3388, http://www.uniprot.org/uniprot/P54764	https://www.genenames.org/data/gene‐symbol‐report/#!/hgnc_id/HGNC:3389, http://www.uniprot.org/uniprot/P54756	https://www.genenames.org/data/gene‐symbol‐report/#!/hgnc_id/HGNC:19296, http://www.uniprot.org/uniprot/Q9UF33	https://www.genenames.org/data/gene‐symbol‐report/#!/hgnc_id/HGNC:3390, http://www.uniprot.org/uniprot/Q15375
EC number	http://www.genome.jp/dbget‐bin/www_bget?ec:2.7.10.1	http://www.genome.jp/dbget‐bin/www_bget?ec:2.7.10.1	http://www.genome.jp/dbget‐bin/www_bget?ec:2.7.10.1	http://www.genome.jp/dbget‐bin/www_bget?ec:2.7.10.1	http://www.genome.jp/dbget‐bin/www_bget?ec:2.7.10.1	http://www.genome.jp/dbget‐bin/www_bget?ec:2.7.10.1	http://www.genome.jp/dbget‐bin/www_bget?ec:2.7.10.1

**Table:** 

Nomenclature	http://www.guidetopharmacology.org/GRAC/ObjectDisplayForward?objectId=1828	http://www.guidetopharmacology.org/GRAC/ObjectDisplayForward?objectId=1829	http://www.guidetopharmacology.org/GRAC/ObjectDisplayForward?objectId=1830	http://www.guidetopharmacology.org/GRAC/ObjectDisplayForward?objectId=1831	http://www.guidetopharmacology.org/GRAC/ObjectDisplayForward?objectId=1832	http://www.guidetopharmacology.org/GRAC/ObjectDisplayForward?objectId=1833	http://www.guidetopharmacology.org/GRAC/ObjectDisplayForward?objectId=1834
Common abbreviation	EphA8	EphA10	EphB1	EphB2	EphB3	EphB4	EphB6
HGNC, UniProt	https://www.genenames.org/data/gene‐symbol‐report/#!/hgnc_id/HGNC:3391, http://www.uniprot.org/uniprot/P29322	https://www.genenames.org/data/gene‐symbol‐report/#!/hgnc_id/HGNC:19987, http://www.uniprot.org/uniprot/Q5JZY3	https://www.genenames.org/data/gene‐symbol‐report/#!/hgnc_id/HGNC:3392, http://www.uniprot.org/uniprot/P54762	https://www.genenames.org/data/gene‐symbol‐report/#!/hgnc_id/HGNC:3393, http://www.uniprot.org/uniprot/P29323	https://www.genenames.org/data/gene‐symbol‐report/#!/hgnc_id/HGNC:3394, http://www.uniprot.org/uniprot/P54753	https://www.genenames.org/data/gene‐symbol‐report/#!/hgnc_id/HGNC:3395, http://www.uniprot.org/uniprot/P54760	https://www.genenames.org/data/gene‐symbol‐report/#!/hgnc_id/HGNC:3396, http://www.uniprot.org/uniprot/O15197
EC number	http://www.genome.jp/dbget‐bin/www_bget?ec:2.7.10.1	http://www.genome.jp/dbget‐bin/www_bget?ec:2.7.10.1	http://www.genome.jp/dbget‐bin/www_bget?ec:2.7.10.1	http://www.genome.jp/dbget‐bin/www_bget?ec:2.7.10.1	http://www.genome.jp/dbget‐bin/www_bget?ec:2.7.10.1	http://www.genome.jp/dbget‐bin/www_bget?ec:2.7.10.1	http://www.genome.jp/dbget‐bin/www_bget?ec:2.7.10.1
Inhibitors	–	–	http://www.guidetopharmacology.org/GRAC/LigandDisplayForward?ligandId=8141 (pIC_50_ 9) [http://www.ncbi.nlm.nih.gov/pubmed/19788238?dopt=AbstractPlus]	–	–	http://www.guidetopharmacology.org/GRAC/LigandDisplayForward?ligandId=7944 (pIC_50_ 8.9) [http://www.ncbi.nlm.nih.gov/pubmed/17575237?dopt=AbstractPlus]	–

## http://www.guidetopharmacology.org/GRAC/FamilyDisplayForward?familyId=794

### Overview

Ret proto‐oncogene (Rearranged during transfection) is a transmembrane tyrosine kinase enzyme which is employed as a signalling partner for members of the http://www.guidetopharmacology.org/GRAC/FamilyDisplayForward?familyId=314. Ligand‐activated GFR appears to recruit Ret as a dimer, leading to activation of further intracellular signalling pathways. Ret appears to be involved in neural crest development, while mutations may be involved in multiple endocrine neoplasia, Hirschsprung&apos;s disease, and medullary thyroid carcinoma.

**Table:** 

Nomenclature	http://www.guidetopharmacology.org/GRAC/ObjectDisplayForward?objectId=2185
Common abbreviation	Ret
HGNC, UniProt	https://www.genenames.org/data/gene‐symbol‐report/#!/hgnc_id/HGNC:9967, http://www.uniprot.org/uniprot/P07949
EC number	http://www.genome.jp/dbget‐bin/www_bget?ec:2.7.10.1
Inhibitors	http://www.guidetopharmacology.org/GRAC/LigandDisplayForward?ligandId=5706 (pIC_50_ 8.3) [http://www.ncbi.nlm.nih.gov/pubmed/19107952?dopt=AbstractPlus]

### Comments

A number of tyrosine kinase inhibitors targeting RET have been described [http://www.ncbi.nlm.nih.gov/pubmed/21960212?dopt=AbstractPlus].

## http://www.guidetopharmacology.org/GRAC/FamilyDisplayForward?familyId=795

### Overview

The ‘related to tyrosine kinase receptor’ (Ryk) is structurally atypical of the family of RTKs, particularly in the activation and ATP‐binding domains. RYK has been suggested to lack kinase activity and appears to be involved, with FZD8, in the Wnt signalling system [http://www.ncbi.nlm.nih.gov/pubmed/21132015?dopt=AbstractPlus].

**Table:** 

Nomenclature	http://www.guidetopharmacology.org/GRAC/ObjectDisplayForward?objectId=1849
Common abbreviation	RYK
HGNC, UniProt	https://www.genenames.org/data/gene‐symbol‐report/#!/hgnc_id/HGNC:10481, http://www.uniprot.org/uniprot/P34925
EC number	http://www.genome.jp/dbget‐bin/www_bget?ec:2.7.10.1

### Comments

Thus far, no selective RYK inhibitors have been described.

## http://www.guidetopharmacology.org/GRAC/FamilyDisplayForward?familyId=331

### Overview

Discoidin domain receptors 1 and 2 (DDR1 and DDR2) are structurally‐related membrane protein tyrosine kinases activated by collagen. Collagen is probably the most abundant protein in man, with at least 29 families of genes encoding proteins, which undergo splice variation and post‐translational processing, and may exist in monomeric or polymeric forms, producing a triple‐stranded, twine‐like structure. In man, principal family members include http://www.guidetopharmacology.org/GRAC/LigandDisplayForward?ligandId=4898 (https://www.genenames.org/data/gene‐symbol‐report/#!/hgnc_id/HGNC:2197, http://www.uniprot.org/uniprot/P02452), http://www.guidetopharmacology.org/GRAC/LigandDisplayForward?ligandId=4899 (https://www.genenames.org/data/gene‐symbol‐report/#!/hgnc_id/HGNC:2200, http://www.uniprot.org/uniprot/P02458), http://www.guidetopharmacology.org/GRAC/LigandDisplayForward?ligandId=4900 (https://www.genenames.org/data/gene‐symbol‐report/#!/hgnc_id/HGNC:2201, http://www.uniprot.org/uniprot/P02461) and http://www.guidetopharmacology.org/GRAC/LigandDisplayForward?ligandId=4901 (https://www.genenames.org/data/gene‐symbol‐report/#!/hgnc_id/HGNC:2202, http://www.uniprot.org/uniprot/P02462).

**Table:** 

Nomenclature	http://www.guidetopharmacology.org/GRAC/ObjectDisplayForward?objectId=1843	http://www.guidetopharmacology.org/GRAC/ObjectDisplayForward?objectId=1844
Common abbreviation	DDR1	DDR2
HGNC, UniProt	https://www.genenames.org/data/gene‐symbol‐report/#!/hgnc_id/HGNC:2730, http://www.uniprot.org/uniprot/Q08345	https://www.genenames.org/data/gene‐symbol‐report/#!/hgnc_id/HGNC:2731, http://www.uniprot.org/uniprot/Q16832
EC number	http://www.genome.jp/dbget‐bin/www_bget?ec:2.7.10.1	http://www.genome.jp/dbget‐bin/www_bget?ec:2.7.10.1

### Comments

The tyrosine kinase inhibitors of DDR, http://www.guidetopharmacology.org/GRAC/LigandDisplayForward?ligandId=5687 and http://www.guidetopharmacology.org/GRAC/LigandDisplayForward?ligandId=5697, were identified from proteomic analysis [http://www.ncbi.nlm.nih.gov/pubmed/18938156?dopt=AbstractPlus]. Other collagen receptors include glycoprotein VI (http://www.uniprot.org/uniprot/Q9HCN6), leukocyte‐associated immunoglobulin‐like receptor 1 (http://www.uniprot.org/uniprot/Q6GTX8), leukocyte‐associated immunoglobulin‐like receptor 2 (http://www.uniprot.org/uniprot/Q6ISS4) and osteoclast‐associated immunoglobulin‐like receptor (http://www.uniprot.org/uniprot/Q8IYS5).

## http://www.guidetopharmacology.org/GRAC/FamilyDisplayForward?familyId=796

**Table:** 

Nomenclature	http://www.guidetopharmacology.org/GRAC/ObjectDisplayForward?objectId=1840
Common abbreviation	ROS
HGNC, UniProt	https://www.genenames.org/data/gene‐symbol‐report/#!/hgnc_id/HGNC:10261, http://www.uniprot.org/uniprot/P08922
EC number	http://www.genome.jp/dbget‐bin/www_bget?ec:2.7.10.1

### Comments

http://www.guidetopharmacology.org/GRAC/LigandDisplayForward?ligandId=4903 is a tyrosine kinase inhibitor, anti‐cancer drug targeting ALK and ROS1.

## http://www.guidetopharmacology.org/GRAC/FamilyDisplayForward?familyId=658

### Overview

The LMR kinases are unusual amongst the RTKs in possessing a short extracellular domain and extended intracellular domain (hence the ‘Lemur’ name reflecting the long tail). A precise function for these receptors has yet to be defined, although LMR1 was identified as a potential marker of apoptosis [http://www.ncbi.nlm.nih.gov/pubmed/9444961?dopt=AbstractPlus], giving rise to the name AATYK (Apoptosis‐associated tyrosine kinase); while over‐expression induces differentiation in neuroblastoma cells [http://www.ncbi.nlm.nih.gov/pubmed/10837911?dopt=AbstractPlus].

**Table:** 

Nomenclature	http://www.guidetopharmacology.org/GRAC/ObjectDisplayForward?objectId=1922	http://www.guidetopharmacology.org/GRAC/ObjectDisplayForward?objectId=2056	http://www.guidetopharmacology.org/GRAC/ObjectDisplayForward?objectId=2057
Common abbreviation	Lmr1	Lmr2	Lmr3
HGNC, UniProt	https://www.genenames.org/data/gene‐symbol‐report/#!/hgnc_id/HGNC:21, http://www.uniprot.org/uniprot/Q6ZMQ8	https://www.genenames.org/data/gene‐symbol‐report/#!/hgnc_id/HGNC:17880, http://www.uniprot.org/uniprot/Q8IWU2	https://www.genenames.org/data/gene‐symbol‐report/#!/hgnc_id/HGNC:19295, http://www.uniprot.org/uniprot/Q96Q04
EC number	http://www.genome.jp/dbget‐bin/www_bget?ec:2.7.11.1	http://www.genome.jp/dbget‐bin/www_bget?ec:2.7.11.1	http://www.genome.jp/dbget‐bin/www_bget?ec:2.7.11.1

### Comments

As yet no selective inhibitors of the LMR family have been described.

## http://www.guidetopharmacology.org/GRAC/FamilyDisplayForward?familyId=329

### Overview

The LTK family appear to lack endogenous ligands. LTK is subject to tissue‐specific splice variation, which appears to generate products in distinct subcellular locations. ALK fusions created by gene translocations and rearrangements are associated with many types of cancer, including large cell lymphomas, inflammatory myofibrilastic tumours and non‐small cell lung cancer [http://www.ncbi.nlm.nih.gov/pubmed/23742252?dopt=AbstractPlus].

**Table:** 

Nomenclature	http://www.guidetopharmacology.org/GRAC/ObjectDisplayForward?objectId=1838	http://www.guidetopharmacology.org/GRAC/ObjectDisplayForward?objectId=1839
Common abbreviation	LTK	ALK
HGNC, UniProt	https://www.genenames.org/data/gene‐symbol‐report/#!/hgnc_id/HGNC:6721, http://www.uniprot.org/uniprot/P29376	https://www.genenames.org/data/gene‐symbol‐report/#!/hgnc_id/HGNC:427, http://www.uniprot.org/uniprot/Q9UM73
EC number	http://www.genome.jp/dbget‐bin/www_bget?ec:2.7.10.1	http://www.genome.jp/dbget‐bin/www_bget?ec:2.7.10.1
Inhibitors	–	http://www.guidetopharmacology.org/GRAC/LigandDisplayForward?ligandId=5683 (pIC_50_ 9.3) [http://www.ncbi.nlm.nih.gov/pubmed/19825801?dopt=AbstractPlus], http://www.guidetopharmacology.org/GRAC/LigandDisplayForward?ligandId=8137 (pIC_50_ 9.1) [http://www.ncbi.nlm.nih.gov/pubmed/24432909?dopt=AbstractPlus], http://www.guidetopharmacology.org/GRAC/LigandDisplayForward?ligandId=4903 (pIC_50_ 9) [http://www.ncbi.nlm.nih.gov/pubmed/21812414?dopt=AbstractPlus], http://www.guidetopharmacology.org/GRAC/LigandDisplayForward?ligandId=5714 (p*K*_d_ 9) [http://www.ncbi.nlm.nih.gov/pubmed/22037378?dopt=AbstractPlus], http://www.guidetopharmacology.org/GRAC/LigandDisplayForward?ligandId=8139 (pIC_50_ 8.7) [http://www.ncbi.nlm.nih.gov/pubmed/22564207?dopt=AbstractPlus] http://www.guidetopharmacology.org/GRAC/LigandDisplayForward?ligandId=7397 (pIC_50_ 9.7) [http://www.ncbi.nlm.nih.gov/pubmed/23742252?dopt=AbstractPlus]
Selective inhibitors	–	–
Comments	–	http://www.guidetopharmacology.org/GRAC/LigandDisplayForward?ligandId=4903 appears to be a selective ALK inhibitor acting on the tyrosine kinase activity [http://www.ncbi.nlm.nih.gov/pubmed/21156280?dopt=AbstractPlus]

## http://www.guidetopharmacology.org/GRAC/FamilyDisplayForward?familyId=668

### Overview

Similar to the LMR RTK family, STYK1 has a truncated extracellular domain, but also displays a relatively short intracellular tail beyond the split kinase domain. STYK1 (also known as Novel Oncogene with Kinase‐domain, NOK) has been suggested to co‐localize with activated EGF receptor [http://www.ncbi.nlm.nih.gov/pubmed/22516751?dopt=AbstractPlus].

**Table:** 

Nomenclature	http://www.guidetopharmacology.org/GRAC/ObjectDisplayForward?objectId=2229
Common abbreviation	STYK1
HGNC, UniProt	https://www.genenames.org/data/gene‐symbol‐report/#!/hgnc_id/HGNC:18889, http://www.uniprot.org/uniprot/Q6J9G0
EC number	http://www.genome.jp/dbget‐bin/www_bget?ec:2.7.10.2

### Comments

As yet, no selective inhibitors of STYK1 have been described.

### Further reading on Receptor tyrosine kinases (RTKs)

Bergeron JJ *et al*. (2016) Spatial and Temporal Regulation of Receptor Tyrosine Kinase Activation and Intracellular Signal Transduction. *Annu. Rev. Biochem*. **85**: 573–97 https://www.ncbi.nlm.nih.gov/pubmed/27023845?dopt=AbstractPlus

Carvalho S *et al*. (2016) Immunotherapy of cancer: from monoclonal to oligoclonal cocktails of anti‐cancer antibodies: IUPHAR Review 18. *Br. J. Pharmacol*. **173**: 1407–24 https://www.ncbi.nlm.nih.gov/pubmed/26833433?dopt=AbstractPlus

De Silva DM *et al*. (2017) Targeting the hepatocyte growth factor/Met pathway in cancer. *Biochem. Soc. Trans*. **45**: 855–870 https://www.ncbi.nlm.nih.gov/pubmed/28673936?dopt=AbstractPlus

Eklund L *et al*. (2017) Angiopoietin‐Tie signalling in the cardiovascular and lymphatic systems. *Clin. Sci*. **131**: 87–103 https://www.ncbi.nlm.nih.gov/pubmed/27941161?dopt=AbstractPlus

Katayama R. (2017) Therapeutic strategies and mechanisms of drug resistance in anaplastic lymphoma kinase (ALK)‐rearranged lung cancer. *Pharmacol. Ther*. **177**: 1–8 https://www.ncbi.nlm.nih.gov/pubmed/28185914?dopt=AbstractPlus

Kazlauskas A. (2017) PDGFs and their receptors. *Gene* **614**: 1–7 https://www.ncbi.nlm.nih.gov/pubmed/28267575?dopt=AbstractPlus

Ke EE *et al*. (2016) EGFR as a Pharmacological Target in EGFR‐Mutant Non‐Small‐Cell Lung Cancer: Where Do We Stand Now? *Trends Pharmacol. Sci*. **37**: 887–903 https://www.ncbi.nlm.nih.gov/pubmed/27717507?dopt=AbstractPlus

Kuwano M *et al*. (2016) Overcoming drug resistance to receptor tyrosine kinase inhibitors: Learning from lung cancer. *Pharmacol. Ther*. **161**: 97–110 https://www.ncbi.nlm.nih.gov/pubmed/27000770?dopt=AbstractPlus

Lee DH. (2017) Treatments for EGFR‐mutant non‐small cell lung cancer (NSCLC): The road to a success, paved with failures. *Pharmacol. Ther*. **174**: 1–21 https://www.ncbi.nlm.nih.gov/pubmed/28167215?dopt=AbstractPlus

Nelson KN *et al*. (2017) Receptor Tyrosine Kinases: Translocation Partners in Hematopoietic Disorders. *Trends Mol Med* **23**: 59–79 https://www.ncbi.nlm.nih.gov/pubmed/27988109?dopt=AbstractPlus

Simons M *et al*. (2016) Mechanisms and regulation of endothelial VEGF receptor signalling. *Nat. Rev. Mol. Cell Biol*. **17**: 611–25 https://www.ncbi.nlm.nih.gov/pubmed/27461391?dopt=AbstractPlus

Stricker S *et al*. (2017) ROR‐Family Receptor Tyrosine Kinases. *Curr. Top. Dev. Biol*. **123**: 105–142 https://www.ncbi.nlm.nih.gov/pubmed/28236965?dopt=AbstractPlus

Tan AC *et al*. (2017) Exploiting receptor tyrosine kinase co‐activation for cancer therapy. *Drug Discov. Today* **22**: 72–84 https://www.ncbi.nlm.nih.gov/pubmed/27452454?dopt=AbstractPlus

Álvarez‐Aznar A *et al*. (2017) VEGF Receptor Tyrosine Kinases: Key Regulators of Vascular Function. *Curr. Top. Dev. Biol*. **123**: 433–482 https://www.ncbi.nlm.nih.gov/pubmed/28236974?dopt=AbstractPlus

## http://www.guidetopharmacology.org/GRAC/FamilyDisplayForward?familyId=303

### Overview

Receptor serine/threonine kinases (RTSK), http://www.genome.jp/kegg‐bin/search_brite?option=‐a&search_string=2.7.11.30, respond to particular cytokines, the transforming growth factor β (TGFβ) and bone morphogenetic protein (BMP) families, and may be divided into two subfamilies on the basis of structural similarities. Agonist binding initiates formation of a cell‐surface complex of type I and type II RSTK, possibly heterotetrameric, where where both subunits express serine/threonine kinase activity. The type I receptor serine/threonine kinases are also known as activin receptors or activin receptor‐like kinases, ALKs, for which a systematic nomenclature has been proposed (ALK1‐7). The type II protein phosphorylates the kinase domain of the type I partner (sometimes referred to as the signal propagating subunit), causing displacement of the protein partners, such as the FKBP12 FK506‐binding protein https://www.genenames.org/data/gene‐symbol‐report/#!/hgnc_id/HGNC:3711 (http://www.uniprot.org/uniprot/P62942) and allowing the binding and phosphorylation of particular members of the Smad family. These migrate to the nucleus and act as complexes to regulate gene transcription. Type III receptors, sometimes called co‐receptors or accessory proteins, regulate the signalling of the receptor complex, in either enhancing (for example, presenting the ligand to the receptor) or inhibitory manners. TGFβ family ligand signalling may be inhibited by endogenous proteins, such as http://www.guidetopharmacology.org/GRAC/LigandDisplayForward?ligandId=4933 (https://www.genenames.org/data/gene‐symbol‐report/#!/hgnc_id/HGNC:3971, http://www.uniprot.org/uniprot/P19883), which binds and neutralizes activins to prevent activation of the target receptors.

Endogenous agonists, approximately 30 in man, are often described as paracrine messengers acting close to the source of production. They are characterized by six conserved cysteine residues and are divided into two subfamilies on the basis of sequence comparison and signalling pathways activated, the TGFβ/activin/nodal subfamily and the BMP/GDF (growth/differentiation factor)/MIS (Müllerian inhibiting substance) subfamily. Ligands active at RSTKs appear to be generated as large precursors which undergo complexmaturation processes [http://www.ncbi.nlm.nih.gov/pubmed/18692464?dopt=AbstractPlus]. Some are known to form disulphide‐linked homo‐ and/or heterodimeric complexes. Thus, inhibins are α subunits linked to a variety of β chains, while activins are combinations of β subunits.

## http://www.guidetopharmacology.org/GRAC/FamilyDisplayForward?familyId=318

### Overview

The type I receptor serine/threonine kinases are also known as activin receptors or activin receptor‐like kinases, ALKs, for which a systematic nomenclature has been proposed (ALK1‐7).

**Table:** 

Nomenclature	http://www.guidetopharmacology.org/GRAC/ObjectDisplayForward?objectId=1784	http://www.guidetopharmacology.org/GRAC/ObjectDisplayForward?objectId=1785	http://www.guidetopharmacology.org/GRAC/ObjectDisplayForward?objectId=1786	http://www.guidetopharmacology.org/GRAC/ObjectDisplayForward?objectId=1787	http://www.guidetopharmacology.org/GRAC/ObjectDisplayForward?objectId=1788	http://www.guidetopharmacology.org/GRAC/ObjectDisplayForward?objectId=1789	http://www.guidetopharmacology.org/GRAC/ObjectDisplayForward?objectId=1790
Common abbreviation	ALK1	ALK2	BMPR1A	ALK4	TGFBR1	BMPR1B	ALK7
HGNC, UniProt	https://www.genenames.org/data/gene‐symbol‐report/#!/hgnc_id/HGNC:175, http://www.uniprot.org/uniprot/P37023	https://www.genenames.org/data/gene‐symbol‐report/#!/hgnc_id/HGNC:171, http://www.uniprot.org/uniprot/Q04771	https://www.genenames.org/data/gene‐symbol‐report/#!/hgnc_id/HGNC:1076, http://www.uniprot.org/uniprot/P36894	https://www.genenames.org/data/gene‐symbol‐report/#!/hgnc_id/HGNC:172, http://www.uniprot.org/uniprot/P36896	https://www.genenames.org/data/gene‐symbol‐report/#!/hgnc_id/HGNC:11772, http://www.uniprot.org/uniprot/P36897	https://www.genenames.org/data/gene‐symbol‐report/#!/hgnc_id/HGNC:1077, http://www.uniprot.org/uniprot/O00238	https://www.genenames.org/data/gene‐symbol‐report/#!/hgnc_id/HGNC:18123, http://www.uniprot.org/uniprot/Q8NER5
EC number	http://www.genome.jp/dbget‐bin/www_bget?ec:2.7.11.30	http://www.genome.jp/dbget‐bin/www_bget?ec:2.7.11.30	http://www.genome.jp/dbget‐bin/www_bget?ec:2.7.11.30	http://www.genome.jp/dbget‐bin/www_bget?ec:2.7.11.30	http://www.genome.jp/dbget‐bin/www_bget?ec:2.7.11.30	http://www.genome.jp/dbget‐bin/www_bget?ec:2.7.11.30	http://www.genome.jp/dbget‐bin/www_bget?ec:2.7.11.30
Inhibitors	–	http://www.guidetopharmacology.org/GRAC/LigandDisplayForward?ligandId=8121 (pIC_50_ 7.5) [http://www.ncbi.nlm.nih.gov/pubmed/23639540?dopt=AbstractPlus]	–	–	http://www.guidetopharmacology.org/GRAC/LigandDisplayForward?ligandId=8075 (p*K*_i_ 7.4) [http://www.ncbi.nlm.nih.gov/pubmed/18413796?dopt=AbstractPlus], http://www.guidetopharmacology.org/GRAC/LigandDisplayForward?ligandId=8194 (pIC_50_ 7.1) [http://www.ncbi.nlm.nih.gov/pubmed/16539403?dopt=AbstractPlus]	–	–
Selective inhibitors	–	–	–	http://www.guidetopharmacology.org/GRAC/LigandDisplayForward?ligandId=8107 (pIC_50_ 7.9) [http://www.ncbi.nlm.nih.gov/pubmed/24786585?dopt=AbstractPlus]	http://www.guidetopharmacology.org/GRAC/LigandDisplayForward?ligandId=8107 (pIC_50_ 8) [http://www.ncbi.nlm.nih.gov/pubmed/24786585?dopt=AbstractPlus]	–	–

### Further reading on Type I receptor serine/threonine kinases

Batlle E *et al*. (2019) Transforming Growth Factor‐β Signaling in Immunity and Cancer *Immunity* **50**: 924–940

## http://www.guidetopharmacology.org/GRAC/FamilyDisplayForward?familyId=319

**Table:** 

Nomenclature	http://www.guidetopharmacology.org/GRAC/ObjectDisplayForward?objectId=1791	http://www.guidetopharmacology.org/GRAC/ObjectDisplayForward?objectId=1792	http://www.guidetopharmacology.org/GRAC/ObjectDisplayForward?objectId=1793	http://www.guidetopharmacology.org/GRAC/ObjectDisplayForward?objectId=1794	http://www.guidetopharmacology.org/GRAC/ObjectDisplayForward?objectId=1795
Common abbreviation	ActR2	ActR2B	MISR2	BMPR2	TGFBR2
HGNC, UniProt	https://www.genenames.org/data/gene‐symbol‐report/#!/hgnc_id/HGNC:173, http://www.uniprot.org/uniprot/P27037	https://www.genenames.org/data/gene‐symbol‐report/#!/hgnc_id/HGNC:174, http://www.uniprot.org/uniprot/Q13705	https://www.genenames.org/data/gene‐symbol‐report/#!/hgnc_id/HGNC:465, http://www.uniprot.org/uniprot/Q16671	https://www.genenames.org/data/gene‐symbol‐report/#!/hgnc_id/HGNC:1078, http://www.uniprot.org/uniprot/Q13873	https://www.genenames.org/data/gene‐symbol‐report/#!/hgnc_id/HGNC:11773, http://www.uniprot.org/uniprot/P37173
EC number	http://www.genome.jp/dbget‐bin/www_bget?ec:2.7.11.30	http://www.genome.jp/dbget‐bin/www_bget?ec:2.7.11.30	http://www.genome.jp/dbget‐bin/www_bget?ec:2.7.11.30	http://www.genome.jp/dbget‐bin/www_bget?ec:2.7.11.30	http://www.genome.jp/dbget‐bin/www_bget?ec:2.7.11.30
Antibodies	–	http://www.guidetopharmacology.org/GRAC/LigandDisplayForward?ligandId=8086 (Binding) (p*K*_d_ 11.8) [10]	–	–	–

### Further reading on Type II receptor serine/threonine kinases

Batlle E *et al*. (2019) Transforming Growth Factor‐β Signaling in Immunity and Cancer *Immunity* **50**: 924‐940

## http://www.guidetopharmacology.org/GRAC/FamilyDisplayForward?familyId=798

**Table:** 

Nomenclature	http://www.guidetopharmacology.org/GRAC/ObjectDisplayForward?objectId=1796
Common abbreviation	TGFBR3
HGNC, UniProt	https://www.genenames.org/data/gene‐symbol‐report/#!/hgnc_id/HGNC:11774, http://www.uniprot.org/uniprot/Q03167

## http://www.guidetopharmacology.org/GRAC/FamilyDisplayForward?familyId=797

### Overview

For the receptors listed below, the exact combination of subunits forming the functional heteromeric receptors is unknown.

**Table:** 

Nomenclature	http://www.guidetopharmacology.org/GRAC/ObjectDisplayForward?objectId=2585	>http://www.guidetopharmacology.org/GRAC/ObjectDisplayForward?objectId=2586
Subunits	http://www.guidetopharmacology.org/GRAC/ObjectDisplayForward?objectId=1788 (Type I), http://www.guidetopharmacology.org/GRAC/ObjectDisplayForward?objectId=1796 (Type III), http://www.guidetopharmacology.org/GRAC/ObjectDisplayForward?objectId=1795 (Type II)	>http://www.guidetopharmacology.org/GRAC/ObjectDisplayForward?objectId=1789 (Type I), http://www.guidetopharmacology.org/GRAC/ObjectDisplayForward?objectId=1792 (Type II), http://www.guidetopharmacology.org/GRAC/ObjectDisplayForward?objectId=1791 (Type II), http://www.guidetopharmacology.org/GRAC/ObjectDisplayForward?objectId=1784 (Type I), http://www.guidetopharmacology.org/GRAC/ObjectDisplayForward?objectId=1785 (Type I), http://www.guidetopharmacology.org/GRAC/ObjectDisplayForward?objectId=1786 (Type I), http://www.guidetopharmacology.org/GRAC/ObjectDisplayForward?objectId=1794 (Type II)
Coupling	Smad2, Smad3 [http://www.ncbi.nlm.nih.gov/pubmed/19855013?dopt=AbstractPlus, http://www.ncbi.nlm.nih.gov/pubmed/12809600?dopt=AbstractPlus]	>Smad1, Smad5, Smad8 [http://www.ncbi.nlm.nih.gov/pubmed/19855013?dopt=AbstractPlus, http://www.ncbi.nlm.nih.gov/pubmed/12809600?dopt=AbstractPlus]
Endogenous agonists	http://www.guidetopharmacology.org/GRAC/LigandDisplayForward?ligandId=5060 (https://www.genenames.org/data/gene‐symbol‐report/#!/hgnc_id/HGNC:11766, http://www.uniprot.org/uniprot/P01137), http://www.guidetopharmacology.org/GRAC/LigandDisplayForward?ligandId=5061 (https://www.genenames.org/data/gene‐symbol‐report/#!/hgnc_id/HGNC:11768, http://www.uniprot.org/uniprot/P61812), http://www.guidetopharmacology.org/GRAC/LigandDisplayForward?ligandId=5062 (https://www.genenames.org/data/gene‐symbol‐report/#!/hgnc_id/HGNC:11769, http://www.uniprot.org/uniprot/P10600)	>http://www.guidetopharmacology.org/GRAC/LigandDisplayForward?ligandId=4875 (https://www.genenames.org/data/gene‐symbol‐report/#!/hgnc_id/HGNC:20869, http://www.uniprot.org/uniprot/O95393), http://www.guidetopharmacology.org/GRAC/LigandDisplayForward?ligandId=4881 (https://www.genenames.org/data/gene‐symbol‐report/#!/hgnc_id/HGNC:1069, http://www.uniprot.org/uniprot/P12643), http://www.guidetopharmacology.org/GRAC/LigandDisplayForward?ligandId=4883 (https://www.genenames.org/data/gene‐symbol‐report/#!/hgnc_id/HGNC:1071, http://www.uniprot.org/uniprot/P12644), http://www.guidetopharmacology.org/GRAC/LigandDisplayForward?ligandId=4884 (https://www.genenames.org/data/gene‐symbol‐report/#!/hgnc_id/HGNC:1072, http://www.uniprot.org/uniprot/P22003), http://www.guidetopharmacology.org/GRAC/LigandDisplayForward?ligandId=4885 (https://www.genenames.org/data/gene‐symbol‐report/#!/hgnc_id/HGNC:1073, http://www.uniprot.org/uniprot/P22004), http://www.guidetopharmacology.org/GRAC/LigandDisplayForward?ligandId=4886 (https://www.genenames.org/data/gene‐symbol‐report/#!/hgnc_id/HGNC:1074, http://www.uniprot.org/uniprot/P18075), http://www.guidetopharmacology.org/GRAC/LigandDisplayForward?ligandId=4887 (https://www.genenames.org/data/gene‐symbol‐report/#!/hgnc_id/HGNC:21650, http://www.uniprot.org/uniprot/Q7Z5Y6), http://www.guidetopharmacology.org/GRAC/LigandDisplayForward?ligandId=4888 (https://www.genenames.org/data/gene‐symbol‐report/#!/hgnc_id/HGNC:1075, http://www.uniprot.org/uniprot/P34820), http://www.guidetopharmacology.org/GRAC/LigandDisplayForward?ligandId=4889 (https://www.genenames.org/data/gene‐symbol‐report/#!/hgnc_id/HGNC:4217, http://www.uniprot.org/uniprot/Q9UK05)

**Table:** 

Nomenclature	http://www.guidetopharmacology.org/GRAC/ObjectDisplayForward?objectId=2587	http://www.guidetopharmacology.org/GRAC/ObjectDisplayForward?objectId=2588	http://www.guidetopharmacology.org/GRAC/ObjectDisplayForward?objectId=2589
Subunits	http://www.guidetopharmacology.org/GRAC/ObjectDisplayForward?objectId=1788 (Type I), http://www.guidetopharmacology.org/GRAC/ObjectDisplayForward?objectId=1789 (Type I), http://www.guidetopharmacology.org/GRAC/ObjectDisplayForward?objectId=1792 (Type II), http://www.guidetopharmacology.org/GRAC/ObjectDisplayForward?objectId=1791 (Type II), http://www.guidetopharmacology.org/GRAC/ObjectDisplayForward?objectId=1790 (Type I), http://www.guidetopharmacology.org/GRAC/ObjectDisplayForward?objectId=1786 (Type I), http://www.guidetopharmacology.org/GRAC/ObjectDisplayForward?objectId=1787 (Type I), http://www.guidetopharmacology.org/GRAC/ObjectDisplayForward?objectId=1794 (Type II)	http://www.guidetopharmacology.org/GRAC/ObjectDisplayForward?objectId=1792 (Type II), http://www.guidetopharmacology.org/GRAC/ObjectDisplayForward?objectId=1791 (Type II), http://www.guidetopharmacology.org/GRAC/ObjectDisplayForward?objectId=1790 (Type I), http://www.guidetopharmacology.org/GRAC/ObjectDisplayForward?objectId=1787 (Type I)	http://www.guidetopharmacology.org/GRAC/ObjectDisplayForward?objectId=1793 (Type II), http://www.guidetopharmacology.org/GRAC/ObjectDisplayForward?objectId=1789 (Type I), http://www.guidetopharmacology.org/GRAC/ObjectDisplayForward?objectId=1785 (Type I), http://www.guidetopharmacology.org/GRAC/ObjectDisplayForward?objectId=1786 (Type I)
Coupling	Smad1, Smad5, Smad8 [http://www.ncbi.nlm.nih.gov/pubmed/19855013?dopt=AbstractPlus, http://www.ncbi.nlm.nih.gov/pubmed/12809600?dopt=AbstractPlus]	Smad2, Smad3 [http://www.ncbi.nlm.nih.gov/pubmed/12809600?dopt=AbstractPlus]	Smad1, Smad5, Smad8 [http://www.ncbi.nlm.nih.gov/pubmed/19855013?dopt=AbstractPlus, http://www.ncbi.nlm.nih.gov/pubmed/12809600?dopt=AbstractPlus]
Endogenous agonists	http://www.guidetopharmacology.org/GRAC/LigandDisplayForward?ligandId=4936 (https://www.genenames.org/data/gene‐symbol‐report/#!/hgnc_id/HGNC:4214, http://www.uniprot.org/uniprot/P27539), http://www.guidetopharmacology.org/GRAC/LigandDisplayForward?ligandId=4937 (https://www.genenames.org/data/gene‐symbol‐report/#!/hgnc_id/HGNC:4215, http://www.uniprot.org/uniprot/P55107), http://www.guidetopharmacology.org/GRAC/LigandDisplayForward?ligandId=4938 (https://www.genenames.org/data/gene‐symbol‐report/#!/hgnc_id/HGNC:4218, http://www.uniprot.org/uniprot/Q9NR23), http://www.guidetopharmacology.org/GRAC/LigandDisplayForward?ligandId=4877 (https://www.genenames.org/data/gene‐symbol‐report/#!/hgnc_id/HGNC:4222, http://www.uniprot.org/uniprot/Q7Z4P5), http://www.guidetopharmacology.org/GRAC/LigandDisplayForward?ligandId=4939 (https://www.genenames.org/data/gene‐symbol‐report/#!/hgnc_id/HGNC:4224, http://www.uniprot.org/uniprot/O60383)	http://www.guidetopharmacology.org/GRAC/LigandDisplayForward?ligandId=4857 (https://www.genenames.org/data/gene‐symbol‐report/#!/hgnc_id/HGNC:6066, http://www.uniprot.org/uniprot/P08476), http://www.guidetopharmacology.org/GRAC/LigandDisplayForward?ligandId=4858 (https://www.genenames.org/data/gene‐symbol‐report/#!/hgnc_id/HGNC:6066 https://www.genenames.org/data/gene‐symbol‐report/#!/hgnc_id/HGNC:6067, http://www.uniprot.org/uniprot/P08476 http://www.uniprot.org/uniprot/P09529), http://www.guidetopharmacology.org/GRAC/LigandDisplayForward?ligandId=4859 (https://www.genenames.org/data/gene‐symbol‐report/#!/hgnc_id/HGNC:6067, http://www.uniprot.org/uniprot/P09529), http://www.guidetopharmacology.org/GRAC/LigandDisplayForward?ligandId=5010 (https://www.genenames.org/data/gene‐symbol‐report/#!/hgnc_id/HGNC:6065 https://www.genenames.org/data/gene‐symbol‐report/#!/hgnc_id/HGNC:6066, http://www.uniprot.org/uniprot/P05111 http://www.uniprot.org/uniprot/P08476)	http://www.guidetopharmacology.org/GRAC/LigandDisplayForward?ligandId=6686 (https://www.genenames.org/data/gene‐symbol‐report/#!/hgnc_id/HGNC:464, http://www.uniprot.org/uniprot/P03971)
Comments	–	Activin receptors are heteromeric complexes comprising activin receptor type I and type II subunits.	–

### Further reading on RSTK functional heteromers

Batlle E *et al*. (2019) Transforming Growth Factor‐β Signaling in Immunity and Cancer *Immunity* **50**: 924‐940

### Comments on Receptor serine/threonine kinase (RSTK) family

A number of endogenous inhibitory ligands have been identified for RSTKs, including http://www.guidetopharmacology.org/GRAC/LigandDisplayForward?ligandId=4882 (https://www.genenames.org/data/gene‐symbol‐report/#!/hgnc_id/HGNC:1070, http://www.uniprot.org/uniprot/P12645), http://www.guidetopharmacology.org/GRAC/LigandDisplayForward?ligandId=5005 (https://www.genenames.org/data/gene‐symbol‐report/#!/hgnc_id/HGNC:6065, http://www.uniprot.org/uniprot/P05111), http://www.guidetopharmacology.org/GRAC/LigandDisplayForward?ligandId=5008 (https://www.genenames.org/data/gene‐symbol‐report/#!/hgnc_id/HGNC:6068, http://www.uniprot.org/uniprot/P55103) and inhibin http://www.guidetopharmacology.org/GRAC/LigandDisplayForward?ligandId=5009 (https://www.genenames.org/data/gene‐symbol‐report/#!/hgnc_id/HGNC:24029, http://www.uniprot.org/uniprot/P58166).

An appraisal of small molecule inhibitors of TGFβ and BMP signalling concluded that TGFβ pathway inhibitors were more selective than BMP signalling inhibitors [http://www.ncbi.nlm.nih.gov/pubmed/21740966?dopt=AbstractPlus]. The authors confirmed the selectivity of http://www.guidetopharmacology.org/GRAC/LigandDisplayForward?ligandId=6049 to inhibit TGFβ signalling through ALK4, ALK5, ALK7 [http://www.ncbi.nlm.nih.gov/pubmed/14978253?dopt=AbstractPlus]. http://www.guidetopharmacology.org/GRAC/LigandDisplayForward?ligandId=4907 inhibits BMP signalling through ALK2 and ALK3, it also inhibits AMP kinase [http://www.ncbi.nlm.nih.gov/pubmed/11602624?dopt=AbstractPlus].

**Smads** were identified as mammalian orthologues of Drosophila genes termed “mothers against decapentaplegic” and may be divided into Receptor‐regulated Smads (R‐Smads, including Smad1, Smad2, Smad3, Smad5 and Smad8), Co‐mediated Smad (Co‐Smad, Smad4) and Inhibitory Smads (I‐Smad, Smad6 and Smad7). R‐Smads form heteromeric complexes with Co‐Smad. I‐Smads compete for binding of R‐Smad with both receptors and Co‐Smad.

**Table:** 

**Nomenclature**	**HGNC gene symbol**	**Uniprot ID**	**Other names**
Smad1	https://www.genenames.org/data/gene‐symbol‐report/#!/hgnc_id/HGNC:6767	http://www.uniprot.org/uniprot/Q15797	JV4‐1, MADH1, MADR1
Smad2	https://www.genenames.org/data/gene‐symbol‐report/#!/hgnc_id/HGNC:6768	http://www.uniprot.org/uniprot/Q15796	JV18‐1, MADH2, MADR2
Smad3	https://www.genenames.org/data/gene‐symbol‐report/#!/hgnc_id/HGNC:6769	http://www.uniprot.org/uniprot/P84022	HsT17436, JV15‐2, MADH3
Smad4	https://www.genenames.org/data/gene‐symbol‐report/#!/hgnc_id/HGNC:6770	http://www.uniprot.org/uniprot/Q13485	DPC4, MADH4
Smad5	https://www.genenames.org/data/gene‐symbol‐report/#!/hgnc_id/HGNC:6771	http://www.uniprot.org/uniprot/Q99717	Dwfc, JV5‐1, MADH5
Smad6	https://www.genenames.org/data/gene‐symbol‐report/#!/hgnc_id/HGNC:6772	http://www.uniprot.org/uniprot/O43541	HsT17432, MADH6, MADH7
Smad7	https://www.genenames.org/data/gene‐symbol‐report/#!/hgnc_id/HGNC:6773	http://www.uniprot.org/uniprot/O15105	MADH7, MADH8
Smad8	https://www.genenames.org/data/gene‐symbol‐report/#!/hgnc_id/HGNC:6774	http://www.uniprot.org/uniprot/O15198	MADH6, MADH9

### Further reading on Receptor serine/threonine kinase (RSTK) family

Budi EH *et al*. (2017) Transforming Growth Factor‐β Receptors and Smads: Regulatory Complexity and Functional Versatility. *Trends Cell Biol*. **27**: 658‐672 [https://www.ncbi.nlm.nih.gov/pubmed/28552280?dopt=AbstractPlus]

Chen W *et al*. (2016) Immunoregulation by members of the TGFβ superfamily. *Nat. Rev. Immunol*. **16**: 723‐740 [https://www.ncbi.nlm.nih.gov/pubmed/27885276?dopt=AbstractPlus]

Heger J *et al*. (2016) Molecular switches under TGFβ signalling during progression from cardiac hypertrophy to heart failure. *Br. J. Pharmacol*. **173**: 3‐14 [https://www.ncbi.nlm.nih.gov/pubmed/26431212?dopt=AbstractPlus]

Luo JY *et al*. (2015) Regulators and effectors of bone morphogenetic protein signalling in the cardiovascular system. *J. Physiol. (Lond.)* **593**: 2995‐3011 [https://www.ncbi.nlm.nih.gov/pubmed/25952563?dopt=AbstractPlus]

Macias MJ *et al*. (2015) Structural determinants of Smad function in TGF‐β signaling. *Trends Biochem. Sci*. **40**: 296‐308 [https://www.ncbi.nlm.nih.gov/pubmed/25935112?dopt=AbstractPlus]

Morrell NW *et al*. (2016) Targeting BMP signalling in cardiovascular disease and anaemia. *Nat Rev Cardiol* **13**: 106‐20 [https://www.ncbi.nlm.nih.gov/pubmed/26461965?dopt=AbstractPlus]

Neuzillet C *et al*. (2015) Targeting the TGFβ pathway for cancer therapy. *Pharmacol. Ther*. **147**: 22‐31 [https://www.ncbi.nlm.nih.gov/pubmed/25444759?dopt=AbstractPlus]

van der Kraan PM. (2017) The changing role of TGFβ in healthy, ageing and osteoarthritic joints. *Nat Rev Rheumatol* **13**: 155‐163 [https://www.ncbi.nlm.nih.gov/pubmed/28148919?dopt=AbstractPlus]

## http://www.guidetopharmacology.org/GRAC/FamilyDisplayForward?familyId=333

### Overview

Receptor tyrosine phosphatases (RTP) are cell‐surface proteins with a single TM region and intracellular phosphotyrosine phosphatase activity. Many family members exhibit constitutive activity in heterologous expression, dephosphorylating intracellular targets such as Src tyrosine kinase (https://www.genenames.org/data/gene‐symbol‐report/#!/hgnc_id/HGNC:11283) to activate signalling cascades. Family members bind components of the extracellular matrix or cell‐surface proteins indicating a role in intercellular communication.

**Table:** 

Nomenclature	http://www.guidetopharmacology.org/GRAC/ObjectDisplayForward?objectId=1850	http://www.guidetopharmacology.org/GRAC/ObjectDisplayForward?objectId=1851	http://www.guidetopharmacology.org/GRAC/ObjectDisplayForward?objectId=1852	http://www.guidetopharmacology.org/GRAC/ObjectDisplayForward?objectId=1853	http://www.guidetopharmacology.org/GRAC/ObjectDisplayForward?objectId=1854	http://www.guidetopharmacology.org/GRAC/ObjectDisplayForward?objectId=1855	http://www.guidetopharmacology.org/GRAC/ObjectDisplayForward?objectId=1856
HGNC, UniProt	https://www.genenames.org/data/gene‐symbol‐report/#!/hgnc_id/HGNC:9664, http://www.uniprot.org/uniprot/P18433	https://www.genenames.org/data/gene‐symbol‐report/#!/hgnc_id/HGNC:9665, http://www.uniprot.org/uniprot/P23467	https://www.genenames.org/data/gene‐symbol‐report/#!/hgnc_id/HGNC:9666, http://www.uniprot.org/uniprot/P08575	https://www.genenames.org/data/gene‐symbol‐report/#!/hgnc_id/HGNC:9668, http://www.uniprot.org/uniprot/P23468	https://www.genenames.org/data/gene‐symbol‐report/#!/hgnc_id/HGNC:9669, http://www.uniprot.org/uniprot/P23469	https://www.genenames.org/data/gene‐symbol‐report/#!/hgnc_id/HGNC:9670, http://www.uniprot.org/uniprot/P10586	https://www.genenames.org/data/gene‐symbol‐report/#!/hgnc_id/HGNC:9671, http://www.uniprot.org/uniprot/P23470
Putative endogenous ligands	–	–	http://www.guidetopharmacology.org/GRAC/LigandDisplayForward?ligandId=6603 (https://www.genenames.org/data/gene‐symbol‐report/#!/hgnc_id/HGNC:6561, http://www.uniprot.org/uniprot/P09382) [http://www.ncbi.nlm.nih.gov/pubmed/10369126?dopt=AbstractPlus]	http://www.guidetopharmacology.org/GRAC/LigandDisplayForward?ligandId=6607 (https://www.genenames.org/data/gene‐symbol‐report/#!/hgnc_id/HGNC:25042, http://www.uniprot.org/uniprot/Q9NT99) [http://www.ncbi.nlm.nih.gov/pubmed/20139422?dopt=AbstractPlus]	–	http://www.guidetopharmacology.org/GRAC/LigandDisplayForward?ligandId=6607 (https://www.genenames.org/data/gene‐symbol‐report/#!/hgnc_id/HGNC:25042, http://www.uniprot.org/uniprot/Q9NT99) [http://www.ncbi.nlm.nih.gov/pubmed/20139422?dopt=AbstractPlus]	http://www.guidetopharmacology.org/GRAC/LigandDisplayForward?ligandId=6609 (https://www.genenames.org/data/gene‐symbol‐report/#!/hgnc_id/HGNC:2173, http://www.uniprot.org/uniprot/Q9P232), http://www.guidetopharmacology.org/GRAC/LigandDisplayForward?ligandId=6612 (https://www.genenames.org/data/gene‐symbol‐report/#!/hgnc_id/HGNC:2174, http://www.uniprot.org/uniprot/Q8IWV2), http://www.guidetopharmacology.org/GRAC/LigandDisplayForward?ligandId=6613 (https://www.genenames.org/data/gene‐symbol‐report/#!/hgnc_id/HGNC:2175, http://www.uniprot.org/uniprot/O94779), http://www.guidetopharmacology.org/GRAC/LigandDisplayForward?ligandId=6614 (https://www.genenames.org/data/gene‐symbol‐report/#!/hgnc_id/HGNC:2176, http://www.uniprot.org/uniprot/Q9UQ52) [http://www.ncbi.nlm.nih.gov/pubmed/20133774?dopt=AbstractPlus]
Inhibitors	–	–	–	–	–	http://www.guidetopharmacology.org/GRAC/LigandDisplayForward?ligandId=8770 (pIC_50_ 5.9) [http://www.ncbi.nlm.nih.gov/pubmed/18579388?dopt=AbstractPlus]	http://www.guidetopharmacology.org/GRAC/LigandDisplayForward?ligandId=8831 (p*K*_i_ 5.6) [http://www.ncbi.nlm.nih.gov/pubmed/21882820?dopt=AbstractPlus]

**Table:** 

Nomenclature	http://www.guidetopharmacology.org/GRAC/ObjectDisplayForward?objectId=1857	http://www.guidetopharmacology.org/GRAC/ObjectDisplayForward?objectId=1858	http://www.guidetopharmacology.org/GRAC/ObjectDisplayForward?objectId=1859	http://www.guidetopharmacology.org/GRAC/ObjectDisplayForward?objectId=1860	http://www.guidetopharmacology.org/GRAC/ObjectDisplayForward?objectId=1861	http://www.guidetopharmacology.org/GRAC/ObjectDisplayForward?objectId=1862	http://www.guidetopharmacology.org/GRAC/ObjectDisplayForward?objectId=1863
HGNC, UniProt	https://www.genenames.org/data/gene‐symbol‐report/#!/hgnc_id/HGNC:9672, http://www.uniprot.org/uniprot/Q9HD43	https://www.genenames.org/data/gene‐symbol‐report/#!/hgnc_id/HGNC:9673, http://www.uniprot.org/uniprot/Q12913	https://www.genenames.org/data/gene‐symbol‐report/#!/hgnc_id/HGNC:9674, http://www.uniprot.org/uniprot/Q15262	https://www.genenames.org/data/gene‐symbol‐report/#!/hgnc_id/HGNC:9675, http://www.uniprot.org/uniprot/P28827	https://www.genenames.org/data/gene‐symbol‐report/#!/hgnc_id/HGNC:9676, http://www.uniprot.org/uniprot/Q16849	https://www.genenames.org/data/gene‐symbol‐report/#!/hgnc_id/HGNC:9677, http://www.uniprot.org/uniprot/Q92932	https://www.genenames.org/data/gene‐symbol‐report/#!/hgnc_id/HGNC:9678, http://www.uniprot.org/uniprot/Q16827
Putative endogenous ligands	–	–	http://www.guidetopharmacology.org/GRAC/LigandDisplayForward?ligandId=6619 (https://www.genenames.org/data/gene‐symbol‐report/#!/hgnc_id/HGNC:6563, http://www.uniprot.org/uniprot/P17931), http://www.guidetopharmacology.org/GRAC/LigandDisplayForward?ligandId=6615 (https://www.genenames.org/data/gene‐symbol‐report/#!/hgnc_id/HGNC:6564, http://www.uniprot.org/uniprot/Q08380) [http://www.ncbi.nlm.nih.gov/pubmed/21094132?dopt=AbstractPlus]	–	–	–	–
Inhibitors	–	–	–	http://www.guidetopharmacology.org/GRAC/LigandDisplayForward?ligandId=8856 (pIC_50_ 5.2) [http://www.ncbi.nlm.nih.gov/pubmed/23713581?dopt=AbstractPlus]	–	–	–

**Table:** 

Nomenclature	http://www.guidetopharmacology.org/GRAC/ObjectDisplayForward?objectId=1864	http://www.guidetopharmacology.org/GRAC/ObjectDisplayForward?objectId=1865	http://www.guidetopharmacology.org/GRAC/ObjectDisplayForward?objectId=1866	http://www.guidetopharmacology.org/GRAC/ObjectDisplayForward?objectId=1867	http://www.guidetopharmacology.org/GRAC/ObjectDisplayForward?objectId=1868	http://www.guidetopharmacology.org/GRAC/ObjectDisplayForward?objectId=1869
HGNC, UniProt	https://www.genenames.org/data/gene‐symbol‐report/#!/hgnc_id/HGNC:9679, http://www.uniprot.org/uniprot/Q9UMZ3	https://www.genenames.org/data/gene‐symbol‐report/#!/hgnc_id/HGNC:9680, http://www.uniprot.org/uniprot/Q15256	https://www.genenames.org/data/gene‐symbol‐report/#!/hgnc_id/HGNC:9681, http://www.uniprot.org/uniprot/Q13332	https://www.genenames.org/data/gene‐symbol‐report/#!/hgnc_id/HGNC:9682, http://www.uniprot.org/uniprot/O14522	https://www.genenames.org/data/gene‐symbol‐report/#!/hgnc_id/HGNC:9683, http://www.uniprot.org/uniprot/Q92729	https://www.genenames.org/data/gene‐symbol‐report/#!/hgnc_id/HGNC:9685, http://www.uniprot.org/uniprot/P23471
Putative endogenous ligands	–	–	http://www.guidetopharmacology.org/GRAC/LigandDisplayForward?ligandId=6616 (https://www.genenames.org/data/gene‐symbol‐report/#!/hgnc_id/HGNC:2465, http://www.uniprot.org/uniprot/O14594), http://www.guidetopharmacology.org/GRAC/LigandDisplayForward?ligandId=6607 (https://www.genenames.org/data/gene‐symbol‐report/#!/hgnc_id/HGNC:25042, http://www.uniprot.org/uniprot/Q9NT99) [http://www.ncbi.nlm.nih.gov/pubmed/20139422?dopt=AbstractPlus, http://www.ncbi.nlm.nih.gov/pubmed/19833921?dopt=AbstractPlus]	–	–	http://www.guidetopharmacology.org/GRAC/LigandDisplayForward?ligandId=6617 (https://www.genenames.org/data/gene‐symbol‐report/#!/hgnc_id/HGNC:2171, http://www.uniprot.org/uniprot/Q12860), http://www.guidetopharmacology.org/GRAC/LigandDisplayForward?ligandId=6618 (https://www.genenames.org/data/gene‐symbol‐report/#!/hgnc_id/HGNC:9630, http://www.uniprot.org/uniprot/C9JR52) (acts as a negative regulator) [http://www.ncbi.nlm.nih.gov/pubmed/20133774?dopt=AbstractPlus, http://www.ncbi.nlm.nih.gov/pubmed/10706604?dopt=AbstractPlus]
Inhibitors	–	–	http://www.guidetopharmacology.org/GRAC/LigandDisplayForward?ligandId=8850 (pIC_50_ 5.4) [78], http://www.guidetopharmacology.org/GRAC/LigandDisplayForward?ligandId=10109 (pIC_50_ 4.4) [http://www.ncbi.nlm.nih.gov/pubmed/9276730?dopt=AbstractPlus]	–	–	–

### Further reading on Receptor tyrosine phosphatase (RTP) family

Papadimitriou E *et al*. (2016) Pleiotrophin and its receptor protein tyrosine phosphatase beta/zeta as regulators of angiogenesis and cancer. *Biochim. Biophys. Acta* **1866**: 252‐265 [https://www.ncbi.nlm.nih.gov/pubmed/27693125?dopt=AbstractPlus]

Stanford SM *et al*. (2017) Targeting Tyrosine Phosphatases: Time to End the Stigma. *Trends Pharmacol. Sci*. **38**: 524‐540 [https://www.ncbi.nlm.nih.gov/pubmed/28412041?dopt=AbstractPlus]

## http://www.guidetopharmacology.org/GRAC/FamilyDisplayForward?familyId=334

### Overview

Dysregulated TNFR signalling is associated with many inflammatory disorders, including some forms of arthritis and inflammatory bowel disease, and targeting TNF has been an effective therapeutic strategy in these diseases and for cancer immunotherapy [http://www.ncbi.nlm.nih.gov/pubmed/23840967?dopt=AbstractPlus, http://www.ncbi.nlm.nih.gov/pubmed/26008591?dopt=AbstractPlus, http://www.ncbi.nlm.nih.gov/pubmed/25169849?dopt=AbstractPlus].

**Table:** 

Nomenclature	http://www.guidetopharmacology.org/GRAC/ObjectDisplayForward?objectId=1870	http://www.guidetopharmacology.org/GRAC/ObjectDisplayForward?objectId=1871	http://www.guidetopharmacology.org/GRAC/ObjectDisplayForward?objectId=1872	http://www.guidetopharmacology.org/GRAC/ObjectDisplayForward?objectId=1873	http://www.guidetopharmacology.org/GRAC/ObjectDisplayForward?objectId=1874	http://www.guidetopharmacology.org/GRAC/ObjectDisplayForward?objectId=1875	http://www.guidetopharmacology.org/GRAC/ObjectDisplayForward?objectId=2322
Systematic nomenclature	TNFRSF1A	TNFRSF1B	TNFRSF3	TNFRSF4	TNFRSF5	TNFRSF6	TNFRSF6B
Common abbreviation	TNFR1	TNFR2	–	–	–	–	–
HGNC, UniProt	https://www.genenames.org/data/gene‐symbol‐report/#!/hgnc_id/HGNC:11916, http://www.uniprot.org/uniprot/P19438	https://www.genenames.org/data/gene‐symbol‐report/#!/hgnc_id/HGNC:11917, http://www.uniprot.org/uniprot/P20333	https://www.genenames.org/data/gene‐symbol‐report/#!/hgnc_id/HGNC:6718, http://www.uniprot.org/uniprot/P36941	https://www.genenames.org/data/gene‐symbol‐report/#!/hgnc_id/HGNC:11918, http://www.uniprot.org/uniprot/P43489	http://www.guidetopharmacology.org/GRAC/ObjectDisplayForward?objectId=1874, http://www.uniprot.org/uniprot/P25942	http://www.guidetopharmacology.org/GRAC/ObjectDisplayForward?objectId=1875, http://www.uniprot.org/uniprot/P25445	https://www.genenames.org/data/gene‐symbol‐report/#!/hgnc_id/HGNC:11921, http://www.uniprot.org/uniprot/O95407
Adaptor proteins	TRADD	TRAF1, TRAF2, TRAF5	TRAF3, TRAF4, TRAF5	TRAF1, TRAF2, TRAF3, TRAF5	TRAF1, TRAF2, TRAF3, TRAF5, TRAF6	FADD	–
Endogenous ligands	http://www.guidetopharmacology.org/GRAC/LigandDisplayForward?ligandId=5064 (https://www.genenames.org/data/gene‐symbol‐report/#!/hgnc_id/HGNC:6709, http://www.uniprot.org/uniprot/P01374), http://www.guidetopharmacology.org/GRAC/LigandDisplayForward?ligandId=5073 (https://www.genenames.org/data/gene‐symbol‐report/#!/hgnc_id/HGNC:11892, http://www.uniprot.org/uniprot/P01375), http://www.guidetopharmacology.org/GRAC/LigandDisplayForward?ligandId=5074 (https://www.genenames.org/data/gene‐symbol‐report/#!/hgnc_id/HGNC:11892, http://www.uniprot.org/uniprot/P01375)	http://www.guidetopharmacology.org/GRAC/LigandDisplayForward?ligandId=5064 (https://www.genenames.org/data/gene‐symbol‐report/#!/hgnc_id/HGNC:6709, http://www.uniprot.org/uniprot/P01374), http://www.guidetopharmacology.org/GRAC/LigandDisplayForward?ligandId=5073 (https://www.genenames.org/data/gene‐symbol‐report/#!/hgnc_id/HGNC:11892, http://www.uniprot.org/uniprot/P01375)	http://www.guidetopharmacology.org/GRAC/LigandDisplayForward?ligandId=5070 (https://www.genenames.org/data/gene‐symbol‐report/#!/hgnc_id/HGNC:11930, http://www.uniprot.org/uniprot/O43557), http://www.guidetopharmacology.org/GRAC/LigandDisplayForward?ligandId=6142 http://www.guidetopharmacology.org/GRAC/LigandDisplayForward?ligandId=6142 (https://www.genenames.org/data/gene‐symbol‐report/#!/hgnc_id/HGNC:6709 https://www.genenames.org/data/gene‐symbol‐report/#!/hgnc_id/HGNC:6711, http://www.uniprot.org/uniprot/P01374 http://www.uniprot.org/uniprot/Q06643)	http://www.guidetopharmacology.org/GRAC/LigandDisplayForward?ligandId=5076 (https://www.genenames.org/data/gene‐symbol‐report/#!/hgnc_id/HGNC:11934, http://www.uniprot.org/uniprot/P23510)	http://www.guidetopharmacology.org/GRAC/LigandDisplayForward?ligandId=5077 (https://www.genenames.org/data/gene‐symbol‐report/#!/hgnc_id/HGNC:11935, http://www.uniprot.org/uniprot/P29965)	http://www.guidetopharmacology.org/GRAC/LigandDisplayForward?ligandId=5078 (https://www.genenames.org/data/gene‐symbol‐report/#!/hgnc_id/HGNC:11936, http://www.uniprot.org/uniprot/P48023)	–
Ligands	–	–	–	http://www.guidetopharmacology.org/GRAC/LigandDisplayForward?ligandId=9092 (Binding) (pIC_50_ 5.9) [http://www.ncbi.nlm.nih.gov/pubmed/24930776?dopt=AbstractPlus]	–	–	–
Comments	–	–	–	The OX40/OX40L pair is involved in late T‐cell costimulatory signaling and both are transiently expressed following antigen recognition, and blocking OX40/OX40L is reported to prevent the development of disease in *in vivo* autoimmune and inflammatory disease models [http://www.ncbi.nlm.nih.gov/pubmed/26215166?dopt=AbstractPlus]	–	–	Decoy receptor for http://www.guidetopharmacology.org/GRAC/LigandDisplayForward?ligandId=5070 (https://www.genenames.org/data/gene‐symbol‐report/#!/hgnc_id/HGNC:11930, http://www.uniprot.org/uniprot/O43557), http://www.guidetopharmacology.org/GRAC/LigandDisplayForward?ligandId=5071 (https://www.genenames.org/data/gene‐symbol‐report/#!/hgnc_id/HGNC:11931, http://www.uniprot.org/uniprot/O95150) and http://www.guidetopharmacology.org/GRAC/LigandDisplayForward?ligandId=5078 (https://www.genenames.org/data/gene‐symbol‐report/#!/hgnc_id/HGNC:11936, http://www.uniprot.org/uniprot/P48023).

**Table:** 

Nomenclature	http://www.guidetopharmacology.org/GRAC/ObjectDisplayForward?objectId=1876	http://www.guidetopharmacology.org/GRAC/ObjectDisplayForward?objectId=1877	http://www.guidetopharmacology.org/GRAC/ObjectDisplayForward?objectId=1878	http://www.guidetopharmacology.org/GRAC/ObjectDisplayForward?objectId=1879	http://www.guidetopharmacology.org/GRAC/ObjectDisplayForward?objectId=1880	http://www.guidetopharmacology.org/GRAC/ObjectDisplayForward?objectId=2323	http://www.guidetopharmacology.org/GRAC/ObjectDisplayForward?objectId=2324
Systematic nomenclature	TNFRSF7	TNFRSF8	TNFRSF9	TNFRSF10A	TNFRSF10B	TNFRSF10C	TNFRSF10D
Common abbreviation	–	–	–	DR4	DR5	–	–
HGNC, UniProt	http://www.guidetopharmacology.org/GRAC/ObjectDisplayForward?objectId=1876, http://www.uniprot.org/uniprot/P26842	https://www.genenames.org/data/gene‐symbol‐report/#!/hgnc_id/HGNC:11923, http://www.uniprot.org/uniprot/P28908	https://www.genenames.org/data/gene‐symbol‐report/#!/hgnc_id/HGNC:11924, http://www.uniprot.org/uniprot/Q07011	https://www.genenames.org/data/gene‐symbol‐report/#!/hgnc_id/HGNC:11904, http://www.uniprot.org/uniprot/O00220	https://www.genenames.org/data/gene‐symbol‐report/#!/hgnc_id/HGNC:11905, http://www.uniprot.org/uniprot/O14763	https://www.genenames.org/data/gene‐symbol‐report/#!/hgnc_id/HGNC:11906, http://www.uniprot.org/uniprot/O14798	https://www.genenames.org/data/gene‐symbol‐report/#!/hgnc_id/HGNC:11907, http://www.uniprot.org/uniprot/Q9UBN6
Adaptor proteins	TRAF2, SIVA	TRAF1, TRAF2, TRAF3, TRAF5	TRAF1, TRAF2, TRAF3	FADD	FADD	–	–
Endogenous ligands	https://www.genenames.org/data/gene‐symbol‐report/#!/hgnc_id/HGNC:11937 (https://www.genenames.org/data/gene‐symbol‐report/#!/hgnc_id/HGNC:11937, http://www.uniprot.org/uniprot/P32970)	http://www.guidetopharmacology.org/GRAC/ObjectDisplayForward?objectId=1877 (https://www.genenames.org/data/gene‐symbol‐report/#!/hgnc_id/HGNC:11938, http://www.uniprot.org/uniprot/P32971)	http://www.guidetopharmacology.org/GRAC/ObjectDisplayForward?objectId=1878 (https://www.genenames.org/data/gene‐symbol‐report/#!/hgnc_id/HGNC:11939, http://www.uniprot.org/uniprot/P41273)	http://www.guidetopharmacology.org/GRAC/LigandDisplayForward?ligandId=5065 (https://www.genenames.org/data/gene‐symbol‐report/#!/hgnc_id/HGNC:11925, http://www.uniprot.org/uniprot/P50591)	–	–	–
Endogenous agonists	–	–	–	–	http://www.guidetopharmacology.org/GRAC/LigandDisplayForward?ligandId=5065 (https://www.genenames.org/data/gene‐symbol‐report/#!/hgnc_id/HGNC:11925, http://www.uniprot.org/uniprot/P50591) [251]	–	–
Agonists	–	–	–	http://www.guidetopharmacology.org/GRAC/LigandDisplayForward?ligandId=8889 [81]	–	–	–
Antibodies	–	http://www.guidetopharmacology.org/GRAC/LigandDisplayForward?ligandId=6772 (Inhibition)	–	–	http://www.guidetopharmacology.org/GRAC/LigandDisplayForward?ligandId=9334 (Agonist) (p*K*_d_ ~8.5) [251]	–	–
Comments	–	–	–	–	–	Decoy receptor for http://www.guidetopharmacology.org/GRAC/LigandDisplayForward?ligandId=5065 (https://www.genenames.org/data/gene‐symbol‐report/#!/hgnc_id/HGNC:11925, http://www.uniprot.org/uniprot/P50591).	Decoy receptor for http://www.guidetopharmacology.org/GRAC/LigandDisplayForward?ligandId=5065 (https://www.genenames.org/data/gene‐symbol‐report/#!/hgnc_id/HGNC:11925, http://www.uniprot.org/uniprot/P50591).

**Table:** 

Nomenclature	http://www.guidetopharmacology.org/GRAC/ObjectDisplayForward?objectId=1881	http://www.guidetopharmacology.org/GRAC/ObjectDisplayForward?objectId=1882	http://www.guidetopharmacology.org/GRAC/ObjectDisplayForward?objectId=1883	http://www.guidetopharmacology.org/GRAC/ObjectDisplayForward?objectId=1884	http://www.guidetopharmacology.org/GRAC/ObjectDisplayForward?objectId=1885	http://www.guidetopharmacology.org/GRAC/ObjectDisplayForward?objectId=1886	http://www.guidetopharmacology.org/GRAC/ObjectDisplayForward?objectId=1887
Systematic nomenclature	TNFRSF11A	TNFRSF11B	TNFRSF25	TNFRSF12A	TNFRSF13B	TNFRSF13C	TNFRSF14
Common abbreviation	RANK	OPG	DR3	–	–	BAFF‐R	HVEM
HGNC, UniProt	https://www.genenames.org/data/gene‐symbol‐report/#!/hgnc_id/HGNC:11908, http://www.uniprot.org/uniprot/Q9Y6Q6	https://www.genenames.org/data/gene‐symbol‐report/#!/hgnc_id/HGNC:11909, http://www.uniprot.org/uniprot/O00300	https://www.genenames.org/data/gene‐symbol‐report/#!/hgnc_id/HGNC:11910, http://www.uniprot.org/uniprot/Q93038	https://www.genenames.org/data/gene‐symbol‐report/#!/hgnc_id/HGNC:18152, http://www.uniprot.org/uniprot/Q9NP84	https://www.genenames.org/data/gene‐symbol‐report/#!/hgnc_id/HGNC:18153, http://www.uniprot.org/uniprot/O14836	https://www.genenames.org/data/gene‐symbol‐report/#!/hgnc_id/HGNC:17755, http://www.uniprot.org/uniprot/Q96RJ3	https://www.genenames.org/data/gene‐symbol‐report/#!/hgnc_id/HGNC:11912, http://www.uniprot.org/uniprot/Q92956
Adaptor proteins	TRAF1, TRAF2, TRAF3, TRAF5, TRAF6	–	TRADD	TRAF1, TRAF2, TRAF3	TRAF2, TRAF5, TRAF6	TRAF3	TRAF2, TRAF3, TRAF5
Endogenous ligands	http://www.guidetopharmacology.org/GRAC/LigandDisplayForward?ligandId=5066 (https://www.genenames.org/data/gene‐symbol‐report/#!/hgnc_id/HGNC:11926, http://www.uniprot.org/uniprot/O14788)	–	http://www.guidetopharmacology.org/GRAC/LigandDisplayForward?ligandId=5071 (https://www.genenames.org/data/gene‐symbol‐report/#!/hgnc_id/HGNC:11931, http://www.uniprot.org/uniprot/O95150)	http://www.guidetopharmacology.org/GRAC/LigandDisplayForward?ligandId=5067 (https://www.genenames.org/data/gene‐symbol‐report/#!/hgnc_id/HGNC:11927, http://www.uniprot.org/uniprot/O43508)	http://www.guidetopharmacology.org/GRAC/LigandDisplayForward?ligandId=5068 (https://www.genenames.org/data/gene‐symbol‐report/#!/hgnc_id/HGNC:11928, http://www.uniprot.org/uniprot/O75888), http://www.guidetopharmacology.org/GRAC/LigandDisplayForward?ligandId=5069 (https://www.genenames.org/data/gene‐symbol‐report/#!/hgnc_id/HGNC:11929, http://www.uniprot.org/uniprot/Q9Y275)	http://www.guidetopharmacology.org/GRAC/LigandDisplayForward?ligandId=5069 (https://www.genenames.org/data/gene‐symbol‐report/#!/hgnc_id/HGNC:11929, http://www.uniprot.org/uniprot/Q9Y275)	http://www.guidetopharmacology.org/GRAC/LigandDisplayForward?ligandId=4891 (https://www.genenames.org/data/gene‐symbol‐report/#!/hgnc_id/HGNC:21087, http://www.uniprot.org/uniprot/Q7Z6A9), http://www.guidetopharmacology.org/GRAC/LigandDisplayForward?ligandId=5070 (https://www.genenames.org/data/gene‐symbol‐report/#!/hgnc_id/HGNC:11930, http://www.uniprot.org/uniprot/O43557), http://www.guidetopharmacology.org/GRAC/LigandDisplayForward?ligandId=5064 (https://www.genenames.org/data/gene‐symbol‐report/#!/hgnc_id/HGNC:6709, http://www.uniprot.org/uniprot/P01374)
Comments	–	Acts as a decoy receptor for http://www.guidetopharmacology.org/GRAC/LigandDisplayForward?ligandId=5066 (https://www.genenames.org/data/gene‐symbol‐report/#!/hgnc_id/HGNC:11926, http://www.uniprot.org/uniprot/O14788) and possibly for http://www.guidetopharmacology.org/GRAC/LigandDisplayForward?ligandId=5065 (https://www.genenames.org/data/gene‐symbol‐report/#!/hgnc_id/HGNC:11925, http://www.uniprot.org/uniprot/P50591).	The only known TNFSF ligand for DR3 is TNF‐like protein 1A (TL1A) [http://www.ncbi.nlm.nih.gov/pubmed/22612445?dopt=AbstractPlus].	–	–	–	–

**Table:** 

Nomenclature	http://www.guidetopharmacology.org/GRAC/ObjectDisplayForward?objectId=1888	http://www.guidetopharmacology.org/GRAC/ObjectDisplayForward?objectId=1889	http://www.guidetopharmacology.org/GRAC/ObjectDisplayForward?objectId=1890	http://www.guidetopharmacology.org/GRAC/ObjectDisplayForward?objectId=1891	http://www.guidetopharmacology.org/GRAC/ObjectDisplayForward?objectId=1892	http://www.guidetopharmacology.org/GRAC/ObjectDisplayForward?objectId=1893
Systematic nomenclature	TNFRSF16	TNFRSF17	TNFRSF18	TNFRSF19	TNFRSF19L	TNFRSF21
Common abbreviation	–	BCMA	GITR	TAJ	–	DR6
HGNC, UniProt	https://www.genenames.org/data/gene‐symbol‐report/#!/hgnc_id/HGNC:7809, http://www.uniprot.org/uniprot/P08138	https://www.genenames.org/data/gene‐symbol‐report/#!/hgnc_id/HGNC:11913, http://www.uniprot.org/uniprot/Q02223	https://www.genenames.org/data/gene‐symbol‐report/#!/hgnc_id/HGNC:11914, http://www.uniprot.org/uniprot/Q9Y5U5	https://www.genenames.org/data/gene‐symbol‐report/#!/hgnc_id/HGNC:11915, http://www.uniprot.org/uniprot/Q9NS68	http://www.guidetopharmacology.org/GRAC/ObjectDisplayForward?objectId=1892, http://www.uniprot.org/uniprot/Q969Z4	https://www.genenames.org/data/gene‐symbol‐report/#!/hgnc_id/HGNC:13469, http://www.uniprot.org/uniprot/O75509
Adaptor proteins	TRAF2, TRAF4, TRAF6	TRAF1, TRAF2, TRAF3, TRAF5, TRAF6	TRAF1, TRAF2, TRAF3, SIVA	TRAF1, TRAF2, TRAF3, TRAF5	TRAF1	TRADD
Endogenous ligands	https://www.genenames.org/data/gene‐symbol‐report/#!/hgnc_id/HGNC:7808 (https://www.genenames.org/data/gene‐symbol‐report/#!/hgnc_id/HGNC:7808, http://www.uniprot.org/uniprot/P01138) (pIC_50_ 6) [http://www.ncbi.nlm.nih.gov/pubmed/22227462?dopt=AbstractPlus], https://www.genenames.org/data/gene‐symbol‐report/#!/hgnc_id/HGNC:1033 (https://www.genenames.org/data/gene‐symbol‐report/#!/hgnc_id/HGNC:1033, http://www.uniprot.org/uniprot/P23560), http://www.guidetopharmacology.org/GRAC/LigandDisplayForward?ligandId=5033 (https://www.genenames.org/data/gene‐symbol‐report/#!/hgnc_id/HGNC:8023, http://www.uniprot.org/uniprot/P20783), http://www.guidetopharmacology.org/GRAC/LigandDisplayForward?ligandId=5034 (https://www.genenames.org/data/gene‐symbol‐report/#!/hgnc_id/HGNC:8024, http://www.uniprot.org/uniprot/P34130)	http://www.guidetopharmacology.org/GRAC/LigandDisplayForward?ligandId=5068 (https://www.genenames.org/data/gene‐symbol‐report/#!/hgnc_id/HGNC:11928, http://www.uniprot.org/uniprot/O75888), http://www.guidetopharmacology.org/GRAC/LigandDisplayForward?ligandId=5069 (https://www.genenames.org/data/gene‐symbol‐report/#!/hgnc_id/HGNC:11929, http://www.uniprot.org/uniprot/Q9Y275)	http://www.guidetopharmacology.org/GRAC/LigandDisplayForward?ligandId=5072 (https://www.genenames.org/data/gene‐symbol‐report/#!/hgnc_id/HGNC:11932, http://www.uniprot.org/uniprot/Q9UNG2)	http://www.guidetopharmacology.org/GRAC/LigandDisplayForward?ligandId=5064 (https://www.genenames.org/data/gene‐symbol‐report/#!/hgnc_id/HGNC:6709, http://www.uniprot.org/uniprot/P01374)	–	–
Comments	One of the two receptor types for the neurotrophins (factors that stimulate neuronal cell survival and differentiation). The other family of neurotrophin receptors are the Trk family of receptor tyrosine kinases.	–	–	Believed to be essential during embryonic development.	Abundant in hematologic tissues. Selective receptor for TNF receptor‐associated factor 1 (TRAF1). Activates the NF‐κB pathway.	–

**Table:** 

Nomenclature	http://www.guidetopharmacology.org/GRAC/ObjectDisplayForward?objectId=1894	http://www.guidetopharmacology.org/GRAC/ObjectDisplayForward?objectId=1895	http://www.guidetopharmacology.org/GRAC/ObjectDisplayForward?objectId=1896	http://www.guidetopharmacology.org/GRAC/ObjectDisplayForward?objectId=2325
Systematic nomenclature	–	–	TNFRS27	–
HGNC, UniProt	–	–	https://www.genenames.org/data/gene‐symbol‐report/#!/hgnc_id/HGNC:17756, http://www.uniprot.org/uniprot/Q9HAV5	https://www.genenames.org/data/gene‐symbol‐report/#!/hgnc_id/HGNC:2895, http://www.uniprot.org/uniprot/Q9UNE0
Adaptor proteins	–	–	TRAF1, TRAF3, TRAF6	TRAF1, TRAF2, TRAF3
Endogenous ligands	–	–	http://www.guidetopharmacology.org/GRAC/ObjectDisplayForward?objectId=1896 (https://www.genenames.org/data/gene‐symbol‐report/#!/hgnc_id/HGNC:3157, http://www.uniprot.org/uniprot/Q92838) [http://www.ncbi.nlm.nih.gov/pubmed/11039935?dopt=AbstractPlus]	http://www.guidetopharmacology.org/GRAC/ObjectDisplayForward?objectId=2325 (https://www.genenames.org/data/gene‐symbol‐report/#!/hgnc_id/HGNC:3157, http://www.uniprot.org/uniprot/Q92838) [http://www.ncbi.nlm.nih.gov/pubmed/11039935?dopt=AbstractPlus]
Comments	Only identified in mouse to date. A potential decoy receptor for the cytotoxic ligand TNFSF10/TRAIL. Does not contain a cytoplasmic death domain so does not induce apoptosis, and does not activate the NF‐κB signalling pathway.	Only identified in mouse to date. A potential decoy receptor for the cytotoxic ligand TNFSF10/TRAIL. Does not contain a cytoplasmic death domain so does not induce apoptosis, and does not activate the NF‐κB signalling pathway.	Receptor for the EDA‐A2 isoform of ectodysplasin encoded by the anhidrotic ectodermal dysplasia (*EDA*) gene.	Cell surface receptor for ectodysplasin A (a morphogen involved in the development of ectodermal tissues, including skin, hair, nails, teeth, and sweat glands).

### Comments

TNFRSF1A is preferentially activated by the shed form of TNF ligand, whereas the membrane‐bound form of TNF serves to activate TNFRSF1A and TNFRSF1B equally. The neurotrophins nerve growth factor (https://www.genenames.org/data/gene‐symbol‐report/#!/hgnc_id/HGNC:7808 (https://www.genenames.org/data/gene‐symbol‐report/#!/hgnc_id/HGNC:7808, http://www.uniprot.org/uniprot/P01138)), brain‐derived neurotrophic factor (https://www.genenames.org/data/gene‐symbol‐report/#!/hgnc_id/HGNC:1033 (https://www.genenames.org/data/gene‐symbol‐report/#!/hgnc_id/HGNC:1033, http://www.uniprot.org/uniprot/P23560)), http://www.guidetopharmacology.org/GRAC/LigandDisplayForward?ligandId=5033 (https://www.genenames.org/data/gene‐symbol‐report/#!/hgnc_id/HGNC:8023, http://www.uniprot.org/uniprot/P20783) (https://www.genenames.org/data/gene‐symbol‐report/#!/hgnc_id/HGNC:8023) and http://www.guidetopharmacology.org/GRAC/LigandDisplayForward?ligandId=5034 (https://www.genenames.org/data/gene‐symbol‐report/#!/hgnc_id/HGNC:8024, http://www.uniprot.org/uniprot/P34130) (NTF4) are structurally unrelated to the TNF ligand superfamily but exert some of their actions through the “low affinity nerve growth factor receptor” (NGFR (TNFRSF16)) as well as through the http://www.guidetopharmacology.org/GRAC/FamilyDisplayForward?familyId=304#show_overview_326 of receptor tyrosine kinases. The endogenous ligands for EDAR and EDA2R are, respectively, the membrane (http://www.uniprot.org/uniprot/Q92838#PRO_0000034538) and secreted (http://www.uniprot.org/uniprot/Q92838#PRO_0000034539) isoforms of Ectodysplasin‐A (EDA, http://www.uniprot.org/uniprot/Q92838).

### Further reading on Tumour necrosis factor (TNF) receptor family

Blaser H *et al*. (2016) TNF and ROS Crosstalk in Inflammation. *Trends Cell Biol*. **26**: 249‐261 https://www.ncbi.nlm.nih.gov/pubmed/26791157?dopt=AbstractPlus

Croft M *et al*. (2017) Beyond TNF: TNF superfamily cytokines as targets for the treatment of rheumatic diseases. *Nat Rev Rheumatol* **13**: 217‐233 https://www.ncbi.nlm.nih.gov/pubmed/28275260?dopt=AbstractPlus

Kalliolias GD *et al*. (2016) TNF biology, pathogenic mechanisms and emerging therapeutic strategies. *Nat Rev Rheumatol* **12**: 49‐62 **[PMID:26656660]**

Olesen CM *et al*. (2016) Mechanisms behind efficacy of tumor necrosis factor inhibitors in inflammatory bowel diseases. *Pharmacol. Ther*. **159**: 110‐9 https://www.ncbi.nlm.nih.gov/pubmed/26808166?dopt=AbstractPlus

von Karstedt S *et al*. (2017) Exploring the TRAILs less travelled: TRAIL in cancer biology and therapy. *Nat. Rev. Cancer* **17**: 352‐366 https://www.ncbi.nlm.nih.gov/pubmed/28536452?dopt=AbstractPlus

## References

[1] Akeson AL *et al* (1996) https://www.ncbi.nlm.nih.gov/pubmed/8940020?dopt=AbstractPlus

[2] Albaugh P *et al* (2012) https://www.ncbi.nlm.nih.gov/pubmed/24900443?dopt=AbstractPlus

[3] Alexopoulou L *et al* (2001) https://www.ncbi.nlm.nih.gov/pubmed/11607032?dopt=AbstractPlus

[4] Apsel B *et al* (2008) https://www.ncbi.nlm.nih.gov/pubmed/18849971?dopt=AbstractPlus

[5] Arena S *et al* (2007) https://www.ncbi.nlm.nih.gov/pubmed/17595299?dopt=AbstractPlus

[6] AstraZeneca AZD1332.

[7] Bach T *et al* (2014) https://www.ncbi.nlm.nih.gov/pubmed/24297249?dopt=AbstractPlus

[8] Baldwin AG *et al* (2017) https://www.ncbi.nlm.nih.gov/pubmed/28943355?dopt=AbstractPlus

[9] Batley BL *et al* (1998) https://www.ncbi.nlm.nih.gov/pubmed/9488112?dopt=AbstractPlus

[10] Berger C *et al* (2013) Patent number: US8388968.

[11] Berl T *et al* (2000) https://www.ncbi.nlm.nih.gov/pubmed/11033834?dopt=AbstractPlus

[12] Bhaskar V *et al* (2008) https://www.ncbi.nlm.nih.gov/pubmed/17786386?dopt=AbstractPlus

[13] Bhaskar V *et al* (2007) https://www.ncbi.nlm.nih.gov/pubmed/18042290?dopt=AbstractPlus

[14] Blume-Jensen P *et al* (2001) https://www.ncbi.nlm.nih.gov/pubmed/11357143?dopt=AbstractPlus

[15] Bold G *et al* (2000) https://www.ncbi.nlm.nih.gov/pubmed/10882357?dopt=AbstractPlus

[16] Bouyain S *et al* (2010) https://www.ncbi.nlm.nih.gov/pubmed/20133774?dopt=AbstractPlus

[17] Brasca MG *et al* (2009) https://www.ncbi.nlm.nih.gov/pubmed/19603809?dopt=AbstractPlus

[18] Breitenstein W *et al* (2015) Patent number: WO2015189265.

[19] Bremer E. (2013) https://www.ncbi.nlm.nih.gov/pubmed/23840967?dopt=AbstractPlus

[20] Brenner D *et al* (2015) https://www.ncbi.nlm.nih.gov/pubmed/26008591?dopt=AbstractPlus

[21] Bruns AM *et al* (2014) https://www.ncbi.nlm.nih.gov/pubmed/25081315?dopt=AbstractPlus

[22] Bryant CE *et al* (2015) https://www.ncbi.nlm.nih.gov/pubmed/25829385?dopt=AbstractPlus

[23] Buchanan SG *et al* (2009) https://www.ncbi.nlm.nih.gov/pubmed/19934279?dopt=AbstractPlus

[24] Buys ES *et al* (2018) https://www.ncbi.nlm.nih.gov/pubmed/29859918?dopt=AbstractPlus

[25] Busby RW *et al* (2010) https://www.ncbi.nlm.nih.gov/pubmed/20863829?dopt=AbstractPlus

[26] Cardarelli JM *et al* (2010) Patent number: US7662381.

[27] Cazorla M *et al* (2011) https://www.ncbi.nlm.nih.gov/pubmed/21505263?dopt=AbstractPlus

[28] Chao YC *et al* (2015) https://www.ncbi.nlm.nih.gov/pubmed/25452496?dopt=AbstractPlus

[29] Chao YC *et al* (2010) https://www.ncbi.nlm.nih.gov/pubmed/20738256?dopt=AbstractPlus

[30] Cho TP *et al* (2010) https://www.ncbi.nlm.nih.gov/pubmed/21028894?dopt=AbstractPlus

[31] Choi SJ *et al* (2010) https://www.ncbi.nlm.nih.gov/pubmed/20153646?dopt=AbstractPlus

[32] Clemens GR *et al* (2009) https://www.ncbi.nlm.nih.gov/pubmed/19107952?dopt=AbstractPlus

[33] Cohen ES *et al* (2012) Patent number: US8263075.

[34] Coll RC *et al* (2015) https://www.ncbi.nlm.nih.gov/pubmed/25686105?dopt=AbstractPlus

[35] Coller BS *et al* (1999) Patent number: US5976532.

[36] Conway JG *et al* (2005) https://www.ncbi.nlm.nih.gov/pubmed/16249345?dopt=AbstractPlus

[37] Coumar MS *et al* (2010) https://www.ncbi.nlm.nih.gov/pubmed/20550212?dopt=AbstractPlus

[38] Cui JJ *et al* (2012) https://www.ncbi.nlm.nih.gov/pubmed/22924734?dopt=AbstractPlus

[39] Cui JJ *et al* (2011) https://www.ncbi.nlm.nih.gov/pubmed/21812414?dopt=AbstractPlus

[40] DaCosta Byfield S *et al* (2004) https://www.ncbi.nlm.nih.gov/pubmed/14978253?dopt=AbstractPlus

[41] Davis BK *et al* (2011) https://www.ncbi.nlm.nih.gov/pubmed/21219188?dopt=AbstractPlus

[42] Davis MI *et al* (2011) https://www.ncbi.nlm.nih.gov/pubmed/22037378?dopt=AbstractPlus

[43] Day E *et al* (2008) https://www.ncbi.nlm.nih.gov/pubmed/18938156?dopt=AbstractPlus

[44] Dechant G *et al* (1997) https://www.ncbi.nlm.nih.gov/pubmed/9204912?dopt=AbstractPlus

[45] Delporte C *et al* (1991) https://www.ncbi.nlm.nih.gov/pubmed/1680722?dopt=AbstractPlus

[46] Derkach DN *et al* (2010) https://www.ncbi.nlm.nih.gov/pubmed/20508901?dopt=AbstractPlus

[47] Deschênes J *et al* (2005) https://www.ncbi.nlm.nih.gov/pubmed/15652659?dopt=AbstractPlus

[48] Ding X *et al* (2012) https://www.ncbi.nlm.nih.gov/pubmed/22516751?dopt=AbstractPlus

[49] Dripps DJ *et al* (1991) https://www.ncbi.nlm.nih.gov/pubmed/1834644?dopt=AbstractPlus

[50] Edelson JD *et al* (2013) https://www.ncbi.nlm.nih.gov/pubmed/23154072?dopt=AbstractPlus

[51] Eldred CD *et al* (1994) https://www.ncbi.nlm.nih.gov/pubmed/7966149?dopt=AbstractPlus

[52] Engers DW *et al* (2013) https://www.ncbi.nlm.nih.gov/pubmed/23639540?dopt=AbstractPlus

[53] Eriksson A *et al* (2012) https://www.ncbi.nlm.nih.gov/pubmed/22864397?dopt=AbstractPlus

[54] Fabbro D *et al* (2012) https://www.ncbi.nlm.nih.gov/pubmed/21960212?dopt=AbstractPlus

[55] Fan Q *et al* (2006) https://www.ncbi.nlm.nih.gov/pubmed/16982323?dopt=AbstractPlus

[56] Feelisch M *et al* (1999) https://www.ncbi.nlm.nih.gov/pubmed/10419542?dopt=AbstractPlus

[57] Fraley ME *et al* (2002) https://www.ncbi.nlm.nih.gov/pubmed/12443771?dopt=AbstractPlus

[58] Frantz WL *et al* (1974) https://www.ncbi.nlm.nih.gov/pubmed/4362846?dopt=AbstractPlus

[59] Friebe A *et al* (1998) https://www.ncbi.nlm.nih.gov/pubmed/9855623?dopt=AbstractPlus

[60] Friebe A *et al* (1998) https://www.ncbi.nlm.nih.gov/pubmed/9855623?dopt=AbstractPlus

[61] Gable KL *et al* (2006) https://www.ncbi.nlm.nih.gov/pubmed/16648580?dopt=AbstractPlus

[62] Galle J *et al* (1999) https://www.ncbi.nlm.nih.gov/pubmed/10369473?dopt=AbstractPlus

[63] Gaozza E *et al* (1997) https://www.ncbi.nlm.nih.gov/pubmed/9444961?dopt=AbstractPlus

[64] García-Echeverría C *et al* (2004) https://www.ncbi.nlm.nih.gov/pubmed/15050915?dopt=AbstractPlus

[65] Garthwaite J *et al* (1995) https://www.ncbi.nlm.nih.gov/pubmed/7544433?dopt=AbstractPlus

[66] Gaul MD *et al* (2003) https://www.ncbi.nlm.nih.gov/pubmed/12639547?dopt=AbstractPlus

[67] Gendreau SB *et al* (2007) https://www.ncbi.nlm.nih.gov/pubmed/17575237?dopt=AbstractPlus

[68] Gerber DE *et al* (2010) https://www.ncbi.nlm.nih.gov/pubmed/21156280?dopt=AbstractPlus

[69] Gingrich DE *et al* (2012) https://www.ncbi.nlm.nih.gov/pubmed/22564207?dopt=AbstractPlus

[70] Goldstein NI *et al* (2006) Patent number: US7060808.

[71] Gomez-Nicola D and Perry VH.. (2014) In Microglia in health and disease. Edited by Tremblay M‐È , Sierra A. : Springer: 437-53 [ISBN: 9781493914296]

[72] Goodman SL *et al* (2002) https://www.ncbi.nlm.nih.gov/pubmed/11855984?dopt=AbstractPlus

[73] Grassot J *et al* (2003) https://www.ncbi.nlm.nih.gov/pubmed/12520021?dopt=AbstractPlus

[74] Graus-Porta D *et al* (1997) https://www.ncbi.nlm.nih.gov/pubmed/9130710?dopt=AbstractPlus

[75] Grumolato L *et al* (2010) https://www.ncbi.nlm.nih.gov/pubmed/21078818?dopt=AbstractPlus

[76] Gundla R *et al* (2008) https://www.ncbi.nlm.nih.gov/pubmed/18500794?dopt=AbstractPlus

[77] Gómez-Nicola D *et al* (2013) https://www.ncbi.nlm.nih.gov/pubmed/23392676?dopt=AbstractPlus

[78] Haftchenary S *et al* (2013) MedChemComm. 987-992.

[79] Hagel M *et al* (2015) https://www.ncbi.nlm.nih.gov/pubmed/25776529?dopt=AbstractPlus

[80] Hamra FK *et al* (1997) https://www.ncbi.nlm.nih.gov/pubmed/9122260?dopt=AbstractPlus

[81] Hanson GJ *et al* (1996) Bioorg Med Chem Lett 6: 1931-6.

[82] Harris LA *et al* (2007) https://www.ncbi.nlm.nih.gov/pubmed/17694454?dopt=AbstractPlus

[83] Harris PA *et al* (2008) https://www.ncbi.nlm.nih.gov/pubmed/18620382?dopt=AbstractPlus

[84] Hayashi F *et al* (2001) https://www.ncbi.nlm.nih.gov/pubmed/11323673?dopt=AbstractPlus

[85] He Y *et al* (2013) https://www.ncbi.nlm.nih.gov/pubmed/23713581?dopt=AbstractPlus

[86] Heil F *et al* (2003) https://www.ncbi.nlm.nih.gov/pubmed/14579267?dopt=AbstractPlus

[87] Heinrich MC *et al* (2012) https://www.ncbi.nlm.nih.gov/pubmed/22745105?dopt=AbstractPlus

[88] Hemmi H *et al* (2002) https://www.ncbi.nlm.nih.gov/pubmed/11812998?dopt=AbstractPlus

[89] Hemmi H *et al* (2000) https://www.ncbi.nlm.nih.gov/pubmed/11130078?dopt=AbstractPlus

[90] Hilberg F *et al* (2008) https://www.ncbi.nlm.nih.gov/pubmed/18559524?dopt=AbstractPlus

[91] Hobbs A *et al* (2004) https://www.ncbi.nlm.nih.gov/pubmed/15337698?dopt=AbstractPlus

[92] Hu J *et al* (2007) https://www.ncbi.nlm.nih.gov/pubmed/17702944?dopt=AbstractPlus

[93] Huang Q *et al* (2014) https://www.ncbi.nlm.nih.gov/pubmed/24432909?dopt=AbstractPlus

[94] Hudkins RL *et al* (2012) https://www.ncbi.nlm.nih.gov/pubmed/22148921?dopt=AbstractPlus

[95] Hutchinson JH *et al* (2003) https://www.ncbi.nlm.nih.gov/pubmed/14561098?dopt=AbstractPlus

[96] Huynh AS *et al* (2012) https://www.ncbi.nlm.nih.gov/pubmed/23098072?dopt=AbstractPlus

[97] Igawa T *et al* (2013) Patent number: US8562991 B2.

[98] Ii M *et al* (2006) https://www.ncbi.nlm.nih.gov/pubmed/16373689?dopt=AbstractPlus

[99] Ingalls RR *et al* (1998) https://www.ncbi.nlm.nih.gov/pubmed/9820516?dopt=AbstractPlus

[100] Jardieu PM *et al* (2004) Patent number: US6703018.

[101] Jeon YH *et al* (2012) https://www.ncbi.nlm.nih.gov/pubmed/22227462?dopt=AbstractPlus

[102] Jiang H *et al* (2017) https://www.ncbi.nlm.nih.gov/pubmed/29021150?dopt=AbstractPlus

[103] Jin CH *et al* (2014) https://www.ncbi.nlm.nih.gov/pubmed/24786585?dopt=AbstractPlus

[104] Jurk M *et al* (2002) https://www.ncbi.nlm.nih.gov/pubmed/12032557?dopt=AbstractPlus

[105] Kambayashi Y *et al* (1989) https://www.ncbi.nlm.nih.gov/pubmed/2542088?dopt=AbstractPlus

[106] Kao Y‐H *et al* (2006) Patent number: WO2006033700.

[107] Kawasaki K *et al* (2000) https://www.ncbi.nlm.nih.gov/pubmed/10644670?dopt=AbstractPlus

[108] Kelly LM *et al* (2002) https://www.ncbi.nlm.nih.gov/pubmed/12124172?dopt=AbstractPlus

[109] Khanwelkar RR *et al* (2010) https://www.ncbi.nlm.nih.gov/pubmed/20570526?dopt=AbstractPlus

[110] Kim N *et al* (2008) https://www.ncbi.nlm.nih.gov/pubmed/18848351?dopt=AbstractPlus

[111] Kim YS *et al* (2011) https://www.ncbi.nlm.nih.gov/pubmed/21094132?dopt=AbstractPlus

[112] Kitagawa D *et al* (2013) https://www.ncbi.nlm.nih.gov/pubmed/23279183?dopt=AbstractPlus

[113] Klein RD *et al* (1997) https://www.ncbi.nlm.nih.gov/pubmed/9192898?dopt=AbstractPlus

[114] Ko FN *et al* (1994) https://www.ncbi.nlm.nih.gov/pubmed/7527671?dopt=AbstractPlus

[115] Koike M *et al* (2013) Patent number: US8501176.

[116] Kubo K *et al* (2005) https://www.ncbi.nlm.nih.gov/pubmed/15743179?dopt=AbstractPlus

[117] Kurachi H *et al* (1992) https://www.ncbi.nlm.nih.gov/pubmed/1530648?dopt=AbstractPlus

[118] Kwon SK *et al* (2010) https://www.ncbi.nlm.nih.gov/pubmed/20139422?dopt=AbstractPlus

[119] Lafleur K *et al* (2009) https://www.ncbi.nlm.nih.gov/pubmed/19788238?dopt=AbstractPlus

[120] Lamphier M *et al* (2014) https://www.ncbi.nlm.nih.gov/pubmed/24342772?dopt=AbstractPlus

[121] Larson P *et al* (2017) ACS Med Chem Lett.

[122] Lee HK *et al* (2014) https://www.ncbi.nlm.nih.gov/pubmed/24532805?dopt=AbstractPlus

[123] Lee K *et al* (2010) https://www.ncbi.nlm.nih.gov/pubmed/20869793?dopt=AbstractPlus

[124] Lee Y *et al* (2004) https://www.ncbi.nlm.nih.gov/pubmed/15634795?dopt=AbstractPlus

[125] Lemmon MA *et al* (2010) https://www.ncbi.nlm.nih.gov/pubmed/20602996?dopt=AbstractPlus

[126] Li D *et al* (2008) https://www.ncbi.nlm.nih.gov/pubmed/18408761?dopt=AbstractPlus

[127] Li HY *et al* (2006) https://www.ncbi.nlm.nih.gov/pubmed/16539403?dopt=AbstractPlus

[128] Li MO *et al* (2008) https://www.ncbi.nlm.nih.gov/pubmed/18692464?dopt=AbstractPlus

[129] Liang G *et al* (2012) https://www.ncbi.nlm.nih.gov/pubmed/22884522?dopt=AbstractPlus

[130] Lin Kc *et al* (1999) https://www.ncbi.nlm.nih.gov/pubmed/10072689?dopt=AbstractPlus

[131] Ling Q *et al* (2008) https://www.ncbi.nlm.nih.gov/pubmed/18579388?dopt=AbstractPlus

[132] Liu G *et al* (2012) https://www.ncbi.nlm.nih.gov/pubmed/24900421?dopt=AbstractPlus

[133] Liu G *et al* (2000) https://www.ncbi.nlm.nih.gov/pubmed/11052808?dopt=AbstractPlus

[134] Liu M *et al* (2009) Patent number: US7598350.

[135] Liu X *et al* (2011) https://www.ncbi.nlm.nih.gov/pubmed/21918175?dopt=AbstractPlus

[136] Lorget F *et al* (2012) https://www.ncbi.nlm.nih.gov/pubmed/23200862?dopt=AbstractPlus

[137] Lu D *et al* (2003) https://www.ncbi.nlm.nih.gov/pubmed/12917408?dopt=AbstractPlus

[138] Maack T *et al* (1987) https://www.ncbi.nlm.nih.gov/pubmed/2823385?dopt=AbstractPlus

[139] MacDonald RG *et al* (1988) https://www.ncbi.nlm.nih.gov/pubmed/2964083?dopt=AbstractPlus

[140] Manthey CL *et al* (2009) https://www.ncbi.nlm.nih.gov/pubmed/19887542?dopt=AbstractPlus

[141] Marathe P *et al* (2010) https://www.ncbi.nlm.nih.gov/pubmed/20166197?dopt=AbstractPlus

[142] Marcinkiewicz C *et al* (2003) https://www.ncbi.nlm.nih.gov/pubmed/12727812?dopt=AbstractPlus

[143] Marsilje TH *et al* (2008) https://www.ncbi.nlm.nih.gov/pubmed/18783949?dopt=AbstractPlus

[144] Marsilje TH *et al* (2013) https://www.ncbi.nlm.nih.gov/pubmed/23742252?dopt=AbstractPlus

[145] Martin FL *et al* (2012) https://www.ncbi.nlm.nih.gov/pubmed/23272242?dopt=AbstractPlus

[146] Martin JH *et al* (2009) Patent number: US7608693.

[147] Martinon F *et al* (2006) https://www.ncbi.nlm.nih.gov/pubmed/16407889?dopt=AbstractPlus

[148] Massa SM *et al* (2010) https://www.ncbi.nlm.nih.gov/pubmed/20407211?dopt=AbstractPlus

[149] Matsuno H *et al* (1994) https://www.ncbi.nlm.nih.gov/pubmed/7955174?dopt=AbstractPlus

[150] McKie PM *et al* (2009) https://www.ncbi.nlm.nih.gov/pubmed/19729120?dopt=AbstractPlus

[151] Melisi D *et al* (2008) https://www.ncbi.nlm.nih.gov/pubmed/18413796?dopt=AbstractPlus

[152] Mendel DB *et al* (2003) https://www.ncbi.nlm.nih.gov/pubmed/12538485?dopt=AbstractPlus

[153] Meng K *et al* (2000) https://www.ncbi.nlm.nih.gov/pubmed/10706604?dopt=AbstractPlus

[154] Miller MW *et al* (2009) https://www.ncbi.nlm.nih.gov/pubmed/19141632?dopt=AbstractPlus

[155] Moffatt P *et al* (2007) https://www.ncbi.nlm.nih.gov/pubmed/17951249?dopt=AbstractPlus

[156] Mollard A *et al* (2011) https://www.ncbi.nlm.nih.gov/pubmed/22247788?dopt=AbstractPlus

[157] Mologni L *et al* (2006) https://www.ncbi.nlm.nih.gov/pubmed/17032739?dopt=AbstractPlus

[158] Morishita Y *et al* (1991) https://www.ncbi.nlm.nih.gov/pubmed/1674870?dopt=AbstractPlus

[159] Morokata T *et al* (2002) https://www.ncbi.nlm.nih.gov/pubmed/12469943?dopt=AbstractPlus

[160] Moustakas A *et al* (2009) https://www.ncbi.nlm.nih.gov/pubmed/19855013?dopt=AbstractPlus

[161] Murthy KS *et al* (1999) https://www.ncbi.nlm.nih.gov/pubmed/10364194?dopt=AbstractPlus

[162] Musumeci F *et al* (2012) https://www.ncbi.nlm.nih.gov/pubmed/23098265?dopt=AbstractPlus

[163] Nagata K *et al* (1996) https://www.ncbi.nlm.nih.gov/pubmed/8939948?dopt=AbstractPlus

[164] Nam HJ *et al* (2011) https://www.ncbi.nlm.nih.gov/pubmed/21306821?dopt=AbstractPlus

[165] Nanda N *et al* (2017) Patent number: WO2017075107A1.

[166] No authors listed (2004) https://www.ncbi.nlm.nih.gov/pubmed/15293871?dopt=AbstractPlus

[167] Ohashi K *et al* (2000) https://www.ncbi.nlm.nih.gov/pubmed/10623794?dopt=AbstractPlus

[168] Ohmichi M *et al* (1993) https://www.ncbi.nlm.nih.gov/pubmed/7683492?dopt=AbstractPlus

[169] Ohno H *et al* (2006) https://www.ncbi.nlm.nih.gov/pubmed/17121910?dopt=AbstractPlus

[170] Olesen SP *et al* (1998) https://www.ncbi.nlm.nih.gov/pubmed/9489619?dopt=AbstractPlus

[171] Olmos-Alonso A *et al* (2016) https://www.ncbi.nlm.nih.gov/pubmed/26747862?dopt=AbstractPlus

[172] Olson LJ *et al* (1996) https://www.ncbi.nlm.nih.gov/pubmed/8700153?dopt=AbstractPlus

[173] Oosting M *et al* (2014) https://www.ncbi.nlm.nih.gov/pubmed/25288745?dopt=AbstractPlus

[174] Ornitz DM *et al* (1996) https://www.ncbi.nlm.nih.gov/pubmed/8663044?dopt=AbstractPlus

[175] Patyna S *et al* (2006) https://www.ncbi.nlm.nih.gov/pubmed/16891463?dopt=AbstractPlus

[176] Pearson MA *et al* (2004) https://www.ncbi.nlm.nih.gov/pubmed/15606337?dopt=AbstractPlus

[177] Pollard JR *et al* (2009) https://www.ncbi.nlm.nih.gov/pubmed/19320489?dopt=AbstractPlus

[178] Poltorak A *et al* (1998) https://www.ncbi.nlm.nih.gov/pubmed/9851930?dopt=AbstractPlus

[179] Ponath PD *et al* (2006) Humanized immunoglobulin reactive with α4β7 integrin. Patent number: US7147851 B1.

[180] Puppo F *et al* (2011) https://www.ncbi.nlm.nih.gov/pubmed/21132015?dopt=AbstractPlus

[181] Párrizas M *et al* (1997) https://www.ncbi.nlm.nih.gov/pubmed/9075698?dopt=AbstractPlus

[182] Queen CL *et al* (1997) Patent number: US5693761.

[183] Raghunath M *et al* (2000) https://www.ncbi.nlm.nih.gov/pubmed/10837911?dopt=AbstractPlus

[184] Roberts WG *et al* (2005) https://www.ncbi.nlm.nih.gov/pubmed/15705896?dopt=AbstractPlus

[185] Rose-John S *et al* (1991) https://www.ncbi.nlm.nih.gov/pubmed/1995637?dopt=AbstractPlus

[186] Russwurm M *et al* (1998) https://www.ncbi.nlm.nih.gov/pubmed/9742221?dopt=AbstractPlus

[187] Sabbah A *et al* (2009) https://www.ncbi.nlm.nih.gov/pubmed/19701189?dopt=AbstractPlus

[188] Sabbatini P *et al* (2009) https://www.ncbi.nlm.nih.gov/pubmed/19825801?dopt=AbstractPlus

[189] Sattler M *et al* (2003) https://www.ncbi.nlm.nih.gov/pubmed/14500382?dopt=AbstractPlus

[190] Scarborough RM *et al* (2000) https://www.ncbi.nlm.nih.gov/pubmed/10999999?dopt=AbstractPlus

[191] Schroder K *et al* (2010) https://www.ncbi.nlm.nih.gov/pubmed/20303873?dopt=AbstractPlus

[192] Schroeder GM *et al* (2009) https://www.ncbi.nlm.nih.gov/pubmed/19260711?dopt=AbstractPlus

[193] Schwandner R *et al* (1999) https://www.ncbi.nlm.nih.gov/pubmed/10364168?dopt=AbstractPlus

[194] Sedger LM *et al* (2014) https://www.ncbi.nlm.nih.gov/pubmed/25169849?dopt=AbstractPlus

[195] Shailubhai K *et al* (2013) https://www.ncbi.nlm.nih.gov/pubmed/23625291?dopt=AbstractPlus

[196] Shen Y *et al* (2009) https://www.ncbi.nlm.nih.gov/pubmed/19833921?dopt=AbstractPlus

[197] Sheriff S *et al* (2011) https://www.ncbi.nlm.nih.gov/pubmed/21882820?dopt=AbstractPlus

[198] Shi Y *et al* (2003) https://www.ncbi.nlm.nih.gov/pubmed/12809600?dopt=AbstractPlus

[199] Singh G *et al* (2006) https://www.ncbi.nlm.nih.gov/pubmed/16778132?dopt=AbstractPlus

[200] Skaper SD *et al* (2000) https://www.ncbi.nlm.nih.gov/pubmed/10987832?dopt=AbstractPlus

[201] Song Y *et al* (2014) https://www.ncbi.nlm.nih.gov/pubmed/24930776?dopt=AbstractPlus

[202] Stasch JP *et al* (2001) https://www.ncbi.nlm.nih.gov/pubmed/11242081?dopt=AbstractPlus

[203] Stasch JP *et al* (2009) https://www.ncbi.nlm.nih.gov/pubmed/19089334?dopt=AbstractPlus

[204] Stasch JP *et al* (2002) https://www.ncbi.nlm.nih.gov/pubmed/12086987?dopt=AbstractPlus

[205] Stevens S *et al* (2009) Patent number: US7582298.

[206] Stitt TN *et al* (1995) https://www.ncbi.nlm.nih.gov/pubmed/7867073?dopt=AbstractPlus

[207] Suga S *et al* (1992) https://www.ncbi.nlm.nih.gov/pubmed/1309330?dopt=AbstractPlus

[208] Takeuchi O *et al* (2010) https://www.ncbi.nlm.nih.gov/pubmed/20303872?dopt=AbstractPlus

[209] Takeuchi O *et al* (2001) https://www.ncbi.nlm.nih.gov/pubmed/11431423?dopt=AbstractPlus

[210] Takeuchi O *et al* (2002) https://www.ncbi.nlm.nih.gov/pubmed/12077222?dopt=AbstractPlus

[211] Tilley JW *et al* J Am Chem Soc 119: 7589-7590

[212] Ting JP *et al* (2008) https://www.ncbi.nlm.nih.gov/pubmed/18341998?dopt=AbstractPlus

[213] Tocker J *et al* (2010) Patent number: US7767206.

[214] Trainer PJ *et al* (2000) https://www.ncbi.nlm.nih.gov/pubmed/10770982?dopt=AbstractPlus

[215] Treanor JJ *et al* (1996) https://www.ncbi.nlm.nih.gov/pubmed/8657309?dopt=AbstractPlus

[216] Trstenjak U *et al* (2013) https://www.ncbi.nlm.nih.gov/pubmed/23644213?dopt=AbstractPlus

[217] Turner AM *et al* (1995) https://www.ncbi.nlm.nih.gov/pubmed/7536489?dopt=AbstractPlus

[218] Uguccioni M *et al* (1997) https://www.ncbi.nlm.nih.gov/pubmed/9276730?dopt=AbstractPlus

[219] Ullrich A *et al* (1990) https://www.ncbi.nlm.nih.gov/pubmed/2158859?dopt=AbstractPlus

[220] Van Roy M *et al* (2015) https://www.ncbi.nlm.nih.gov/pubmed/25994180?dopt=AbstractPlus

[221] Veale CA *et al* (2000) https://www.ncbi.nlm.nih.gov/pubmed/10987424?dopt=AbstractPlus

[222] Verkerke H *et al* (2014) https://www.ncbi.nlm.nih.gov/pubmed/24743494?dopt=AbstractPlus

[223] Vogt J *et al* (2011) https://www.ncbi.nlm.nih.gov/pubmed/21740966?dopt=AbstractPlus

[224] Walzel H *et al* (1999) https://www.ncbi.nlm.nih.gov/pubmed/10369126?dopt=AbstractPlus

[225] Wang EC. (2012) https://www.ncbi.nlm.nih.gov/pubmed/22612445?dopt=AbstractPlus

[226] Wang T *et al* (2012) https://www.ncbi.nlm.nih.gov/pubmed/24900538?dopt=AbstractPlus

[227] Ward AC *et al* (2000) https://www.ncbi.nlm.nih.gov/pubmed/10607680?dopt=AbstractPlus

[228] Webb GJ *et al* (2016) https://www.ncbi.nlm.nih.gov/pubmed/26215166?dopt=AbstractPlus

[229] Weber W *et al* (1991) https://www.ncbi.nlm.nih.gov/pubmed/1849131?dopt=AbstractPlus

[230] Wesche J *et al* (2011) https://www.ncbi.nlm.nih.gov/pubmed/21711248?dopt=AbstractPlus

[231] Whittles CE *et al* (2002) https://www.ncbi.nlm.nih.gov/pubmed/12483548?dopt=AbstractPlus

[232] Wilde MI *et al* (1998) https://www.ncbi.nlm.nih.gov/pubmed/18020592?dopt=AbstractPlus

[233] Wilhelm SM *et al* (2004) https://www.ncbi.nlm.nih.gov/pubmed/15466206?dopt=AbstractPlus

[234] Wittman M *et al* (2005) https://www.ncbi.nlm.nih.gov/pubmed/16134929?dopt=AbstractPlus

[235] Wittman MD *et al* (2009) https://www.ncbi.nlm.nih.gov/pubmed/19778024?dopt=AbstractPlus

[236] Wood ER *et al* (2004) https://www.ncbi.nlm.nih.gov/pubmed/15013000?dopt=AbstractPlus

[237] Wu H *et al* (2010) Patent number: US7659374.

[238] Wyss DF *et al* (1991) https://www.ncbi.nlm.nih.gov/pubmed/1826288?dopt=AbstractPlus

[239] Yakes FM *et al* (2011) https://www.ncbi.nlm.nih.gov/pubmed/21926191?dopt=AbstractPlus

[240] Yan M *et al* (2000) https://www.ncbi.nlm.nih.gov/pubmed/11039935?dopt=AbstractPlus

[241] Yao N *et al* (2009) https://www.ncbi.nlm.nih.gov/pubmed/19055415?dopt=AbstractPlus

[242] Yasuda T *et al* (1993) https://www.ncbi.nlm.nih.gov/pubmed/8485125?dopt=AbstractPlus

[243] Yeh BK *et al* (2003) https://www.ncbi.nlm.nih.gov/pubmed/12591959?dopt=AbstractPlus

[244] Yoshimura A *et al* (1999) https://www.ncbi.nlm.nih.gov/pubmed/10384090?dopt=AbstractPlus

[245] Youm YH *et al* (2015) https://www.ncbi.nlm.nih.gov/pubmed/25686106?dopt=AbstractPlus

[246] Zabel U *et al* (1998) https://www.ncbi.nlm.nih.gov/pubmed/9742212?dopt=AbstractPlus

[247] Zhang G *et al* (2018) https://www.ncbi.nlm.nih.gov/pubmed/29311663?dopt=AbstractPlus

[248] Zhao G *et al* (2011) https://www.ncbi.nlm.nih.gov/pubmed/21900693?dopt=AbstractPlus

[249] Zhong M *et al* (2012) https://www.ncbi.nlm.nih.gov/pubmed/24900456?dopt=AbstractPlus

[250] Zhou G *et al* (2001) https://www.ncbi.nlm.nih.gov/pubmed/11602624?dopt=AbstractPlus

[251] Zhou T *et al* (2001) Patent number: WO2001083560.

[252] Zhou W *et al* (2010) https://www.ncbi.nlm.nih.gov/pubmed/20338520?dopt=AbstractPlus

[253] Zhu X *et al* (2009) https://www.ncbi.nlm.nih.gov/pubmed/19710453?dopt=AbstractPlus

